# Simulated Mass Casualty Incident Triage Exercise for Training Medical Personnel

**DOI:** 10.21980/J82H1R

**Published:** 2020-10-15

**Authors:** Alaina Brinley Rajagopal, Nathan Jasperse, Megan Boysen Osborn

**Affiliations:** * University of California, Irvine, Department of Emergency Medicine, Orange, CA

## Abstract

**Audience:**

The target audience is any medical professional who requires training in mass casualty incident (MCI) triage. This could apply to pre-hospital specialists, nurses, medical students, residents, and physicians.

**Introduction:**

Emergency medicine specialists must be able to triage patients quickly, especially in an MCI scenario. The simple triage and rapid treatment (START) system allows providers to categorize patients according to the urgency with which patients must access limited resources. Providers should be comfortable utilizing the START triage system before an MCI or disaster so that they can be prepared to implement it if necessary. This exercise uses simulation and gamification as instructional strategies to encourage knowledge of and comfort with the START triage system for emergency providers.

**Educational Objectives:**

By the end of this exercise, learners should be able to (1) recite the basic START patient categories (2) discuss the physical exam signs associated with each START category, (3) assign roles to medical providers in a mass casualty scenario, (4) accurately categorize patients into triage categories: green, yellow, red, and black, and (5) manage limited resources when demand exceeds availability.

**Educational Methods:**

Gamification is the use of elements of game design in non-game contexts.^1^ Gamification was implemented in this scenario by assigning participants to roles and teams, while creating an engaging, fun, and competitive environment. The exercise also uses low fidelity simulation (without simulation equipment) to encourage learners to practice using the START triage system in a low stakes environment.^2^ It is possible for the learners to be divided into two groups that each have the same patients, resources, and objectives. The team that finishes triaging all patients first would be declared the winner. However, in our implementation, we completed the exercise as a single group of learners and patients.

**Research Methods:**

Learners were given a survey at the end of implementation and also given the opportunity to discuss feedback with the instructors in a group discussion after completing the exercise. There was no formal assessment completed after the exercise.

**Results:**

Informal feedback was collected at the end of the exercise. Residents and medical students all enjoyed the experience. The feedback was overwhelmingly positive. All participants providing feedback stated they would enjoy participating in the exercise again and suggested that it is implemented annually for review of triage topics. We also received informal feedback for suggested changes which we will discuss in this article. An optional, anonymous survey was given to participants at the end of the exercise. There were six responses. Of those surveyed, 100% of participants stated the effectiveness and value of the exercise was outstanding (a rating of five on a scale of one to five). Regarding the quality of the exercise, and whether the participants felt engaged, 100% of responses gave a rating of five. When asked to consider the relevance of the session, 100% of participants selected a score of five (“I loved this session”). Regarding whether the content was applicable to practice of emergency medicine, 80% of respondents stated the session was highly relevant and 20% of responses selected a score of mostly relevant. One question asked for points of improvement for the session to which there were no responses.

**Discussion:**

Learners were assigned roles in the exercise by the incident commander, fulfilling objective three. The START categories were discussed at the beginning of the exercise by the lead proctor (using PowerPoint) and then utilized throughout the exercise, thus accomplishing objectives one and two. The residents/students filling the triage roles were primarily responsible for fulfilling objective four; however, all participants assisted in categorization of patients throughout the exercise. Finally, objective five was addressed through the various social situations and complications that can be implemented during the exercise. We chose not to implement the additional “radiation contamination” scenario (details available in the article text) due to time constraints; however, this is an additional option to address objective five. The implementation was effective based on informal feedback from participants and proctors as well as evidenced by the responses to the anonymous survey. Learners found the aspects of resource management, review of START triage, repetition of the START triage system, and medical management of various types of trauma informative and meaningful. We received valuable feedback from both learners and proctors, which we will discuss in this article.

**Topics:**

Mass casualty incident, disaster, START, gamification, simulation, emergency medicine, triage, triage category, contamination, teamwork, trauma, projectile trauma, penetrating injury, blunt trauma, intracranial hemorrhage, fracture, trauma in pregnancy, active shooter, radiation, radio communication.

## USER GUIDE

**Table:** 

List of Resources:	
Abstract	1
User Guide	3
Small Group Materials	10
Appendix A: Disaster Triage Exercise Lecture	10
Appendix B: Small Group Application Exercise Cases for Learners	11
Appendix C: Instructions for Proctors	69
Appendix D: Cases, Labs and Imaging for Proctors	76
Appendix E: Roles and Descriptions, Medications for Pharmacist	120
Appendix F: Small Group Application Exercise Answers. Cases, Labs and Imaging Answers for Proctors	128
Pre and Post Exercise Assessment	228
Pre and Post Exercise Assessment Key	230

**Learner Audience:**

Medical Students, Interns, Junior Residents, Senior Residents, Nursing or emergency medical services (EMS) personnel

**Time Required for Implementation:**

The exercise was implemented over a period of two hours during residency conference; however, it could be appropriate for shorter or longer time periods depending on the number of mock patients learners must triage (for example, 30 mock patients for a shorter exercise or 90 mock patients for a longer exercise). The time for implementation is also dependent upon time allotted for debriefing.

**Recommended Number of Learners per Instructor:**

The recommended number of learners per instructor is variable depending on triage scenario role. Some learner positions require an instructor to be present at all times while other learner positions have little to no instructor feedback. For example, the instructors leading the green, yellow, red zones of the activity will need to ask the learners questions and provide feedback and teaching points. For these instructors, 4–6 learners would be recommended. Other positions, such as the incident commander, receive little to no feedback from the instructors in the red, yellow, green hospital zones; however, the incident commander may get some guidance from the instructor proctoring the entire activity as needed or requested by the incident commander. It is recommended to have at least one instructor for each of the positions: red zone, yellow zone, green zone, imaging/labs, admissions, for a minimum of five instructors

**Topics:**

Mass casualty incident, disaster, START, gamification, simulation, emergency medicine, triage, triage category, contamination, teamwork, trauma, projectile trauma, penetrating injury, blunt trauma, intracranial hemorrhage, fracture, trauma in pregnancy, active shooter, radiation, radio communication.

**Objectives:**

By the end of this exercise, learners should be able to:

1. Recite the basic START patient categories
2. Discuss the physical exam signs associated with each START category
3. Assign roles to medical providers in a mass casualty scenario
4. Accurately categorize patients into triage categories: green, yellow, red, and black
5. Manage limited resources when demand exceeds availability.

### Linked objectives and methods

The overall goal for this activity was to teach learners how to manage a large number of patients from an MCI and simultaneously teach triage methods. While it is helpful to review the START triage system in a lecture format, implementing the triage system through practice will reinforce the system in learners’ knowledge scripts.^7^,^8^ Additionally, creating a simulation environment provides a sensation of urgency. The placement of the triage zones (green, yellow, red, black) in geographic regions distant from others teaches learners about resource management and complications with communication in disaster situations. In all, the physicality of acting out an MCI allows learners to begin to approach what a real-life scenario might be like and how they would implement their own systems if they are ever faced with a similar incident.^2^

Objective one: Recite basic START patient categories. For this objective, an instructor utilized a lecture-based platform to introduce the START patient categories to the group of learners. We have included a pre- and post-exercise assessment to determine if the learning methods were effective.

Objective two: Discuss the physical exam signs associated with each START category. For this objective, the instructor again utilized the same lecture-based platform to discuss the vital signs and physical exam signs associated with each triage category. This was also assessed with the pre- and post-exercise assessment.

Objective three: Assign roles to medical providers in a mass casualty scenario. This objective was satisfied by the incident commander position. This position was filled by a senior learner who then selected the roles for the additional learners. It would be reasonable to have a proctor reassign the roles during the exercise so more learners have the opportunity to participate in this objective; however, in our implementation, we had a single incident commander who was a senior resident. Throughout the exercise, learners needed to be re-assigned due to various developments associated with the patients and with resource management. Learners needed to contact the incident commander for these re-assignments which gave more learners the opportunity to assist with role assignment. This objective was assessed informally by all proctors during the exercise. This objective was also assessed by the formal pre- and post-exercise assessment.

Objective four: Accurately categorize patients into triage categories: green, yellow, red, and black. This objective was completed primarily by the learners in the triage area; however, there were patient cases that required re-triage during the exercise due to changing vital signs. This allowed the opportunity for more learners to participate in learning the START categories. This objective was assessed by all proctors during the exercise. Initial triage was assessed by the triage proctor. This objective was also assessed by the formal pre- and post-exercise assessment.

Objective five: Manage limited resources when demand exceeds availability. The exercise intentionally has many more patients than can be immediately triaged, evaluated in the zones, and dispositioned quickly. The triage area can assign these patients fairly quickly which creates an initial backlog of patients in each of the zones. Additionally, in our residency, there were more learner roles than learners; therefore, the incident commander was constantly being pulled in different directions to re-assign learners to different roles, as needed. There were several cases that featured patients with mental illness or agitated states. Resources had to be redirected to manage these scenarios. There was also an additional “radiation contamination” scenario where participants would be notified of radiation contamination (demarcated by an asterisk on the patient sheet) in the middle of the exercise. The learners would then have to isolate, but still treat these patients and develop a plan. There were other scenarios requiring reassignments such as obstetric emergencies which required that a learner would be reassigned to act as an OB/gyn. Overall, there was ample opportunity for making learners feel the stresses of when demand exceeds available resources. This objective was assessed during the debrief portion of the exercise where participants were encouraged to discuss difficulties, successes, patient management, and resource allocation as well as by proctors throughout the exercise.

### Recommended pre-reading for facilitator

At minimum, the authors recommend reviewing the first reference, but would suggest review of all the listed sources.

1. Coti, P. Mass casualty incident triage. WikEM. https://www.wikem.org/wiki/Mass_casualty_incident_triage. Accessed 07/23/2019.
2. Culley JM, Svendsen E. A review of the literature on the validity of mass casualty triage systems with a focus on chemical exposures. *Am J Disaster Med*. 2014;9(2):137–150. doi:10.5055/ajdm.2014.0150.
3. Model Uniform Core Criteria for Mass Casualty Triage. *Disaster Medicine and Public Health Preparedness*. 2011; *5*(2):125–128. doi:10.1001/dmp.2011.41.
4. Lerner EB, Schwartz RB, Coule PL, et al. Mass Casualty Triage: An Evaluation of the Data and Development of a Proposed National Guideline. *Disaster Med Public Health Prep*. 2008;2 (Suppl. 1):S25–S34.

### Learner responsible content (LRC)

Learners should read at least the first reference; however, all references are recommended for pre-reading.

1. Coti, P. Mass casualty incident triage. WikEM. Accessed 07/23/2019. https://www.wikem.org/wiki/Mass_casualty_incident_triage.
2. Culley JM, Svendsen E. A review of the literature on the validity of mass casualty triage systems with a focus on chemical exposures. *American Journal of Disaster Medicine*. 2014;9(2):137–150. doi:10.5055/ajdm.2014.0150.
3. Model uniform core criteria for mass casualty triage. Disaster Med Public Health Prep. 2011;5(2):125–8.

### Associated Materials

- Appendix A: Disaster Triage Exercise Lecture
- Appendix B: Small Group Application Exercise Cases for Learners
- Appendix C: Instructions for Proctors
- Appendix D: Cases, Labs and Imaging for Proctors
- Appendix E: Roles and Descriptions, Medications for Pharmacist
- Appendix F: Small Group Application Exercise Answers. Cases, Labs and Imaging Answers for Proctors

### Methods for game play

1. The exercise begins with a PowerPoint lecture on START triage, which addresses the first learning objective.
  - Learners are told they are going to have a lecture about MCIs and how to manage them.
  - Each triage category is reviewed by a PowerPoint lecture (Appendix A).
2. After a review the triage categories, a staff member runs into the room and shouts, “There was an MCI at ‘insert name’ stadium! Hurry, we need your help!” Learners then have a sense of urgency to begin the exercise.
3. The exercise is now outlined by the lead proctor, again using PowerPoint (Appendix A).
  - The main roles are discussed and the “incident commander” is selected by the lead instructor.
  - The “incident commander” assigns roles by handing out a piece of paper with each role name and description on it. This addresses objective three.
  - The lead proctor presents a brief discussion on radio communication.
4. Next, all learners are escorted outside to set up their triage stations.
  - The “incident commander” passes out handheld radios to the individuals he believes are likely to need them.
5. The “triage lead” (which is a different role from “incident commander”) is in charge of the initial triage area and must categorize all patients coming to the treatment area. This addresses objectives one, two, and four.
  - The “triage lead” also records the patients using a system determined by the “incident commander” and “triage lead.”
  - This position has a designated faculty proctor to guide learners through the process.
6. The patients are then transferred from the triage area to the red/yellow/green/black zone by individuals who have been given the “transporter” role.
7. The triage team and the teams for the red, yellow, and green zones then categorize, diagnose, and treat their patients according to the previously reviewed guidelines. This part of the exercise addresses objective four.
8. Objective five is addressed by roles of “pharmacist,” “hospitalist,” “surgeon,” and by various social scenarios that are presented during the course of the exercise.
  - For example, the role of “pharmacist” requires that all medications are physically brought to a patient in need by one “pharmacist.”
    - If additional “pharmacists” are needed, the “incident commander” must reassign roles; however, the stock of medications remains the same.
  - Another part of the scenario that incorporates limited resources includes that the “hospitalist” must receive a sign out from the red/yellow/green team before a patient can be transported to the hospital.
    - If additional “hospitalists” are needed, roles must be reassigned by the “incident commander.”
  - The role of “surgeon” can initially be assigned to up to three people.
    - Each “surgeon” must complete an entire game of the board game Operation^®^ without activating the associated buzzer for each patient requiring surgery.^9^
    - One additional operating room (OR), which is represented by one Operation^®^ board game, can be added later in the game by the lead proctor, *if* deemed necessary by the “incident commander.”
9. Finally, there are various complicating scenarios that can occur as implemented by the lead proctor.
  - For example, all patients with an asterisk on their patient card can be categorized as radiation-contaminated if the proctor would like to add this complication and teach about radiation and decontamination techniques.
    - In order to do this, the lead proctor must add an asterisk to the corner of each patient card prior to the start of the exercise.
  - Additional complicating scenarios which require reassignment of personnel include an active shooter or an agitated/aggressive patient.

### Results and tips for successful implementation

This exercise was presented during residency conference to a group of approximately 24 emergency medicine residents and medical students. It is best implemented in a group setting with between one and six learners per group; however, all roles must be assigned by a resident “incident commander.” We did not obtain a direct assessment of learner acquisition of knowledge. We observed high learner engagement and enjoyment.

There were several modifications suggested by learners and faculty after implementation. First, we used a size 12 font on our “patient” worksheets with scratch-off stickers. It was suggested that we use larger font and have greater separation between each question and answer. It was observed that with the close spacing of the questions and answers, learners accidentally scratched off more than the answer for the question on which they were working. We have adjusted this in the published version of the exercise (Appendix B). Another suggestion was that we provide learners with a hard copy of the START triage system so learners could reference the sheet/card during the exercise. We have included this suggested handout as a part of this article (See “Pearls” section below). Next, it was suggested that some sort of simulation of a patient’s body should be used instead of a sheet of paper. For example, a brick, a bag of flour, a bag of sand, a stuffed animal, or a mannequin. Use of any of these as weighted simulated patients would require significant resources and personnel to organize and maintain. All patients were carried by hand by the individuals in the “transporter” roles. If additional weight were applied to the patients, wheelchairs or gurneys might be needed to assist “transporters.” It may also be helpful to provide coins or some sort of implement for scratching off the scratch-off stickers since learners were not prepared for this and needed to improvise. That said, improvisation may be considered a part of the learning process. Finally, ensure the Operation games have batteries. One of our games did not have batteries and, thus, did not “buzz” when mistakes were made by the surgeon.

### Pearls

See exercise handout.

## Supplementary Information



## Figures and Tables

**Figure:**
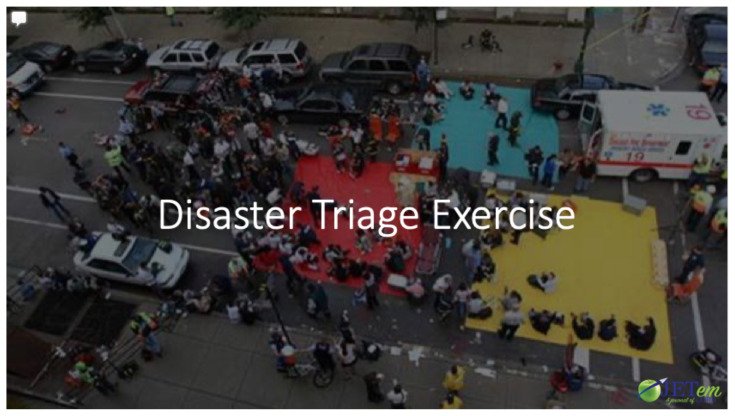
Please see associated PowerPoint file

**Figure 1:**
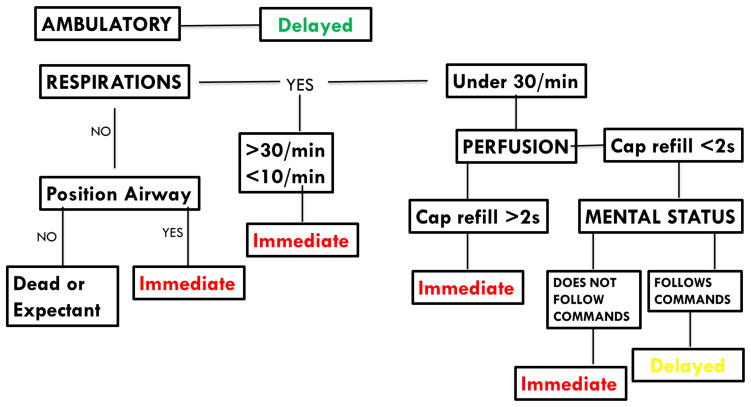
START triage handout. To be distributed to learners prior to start of exercise

**Figure:**
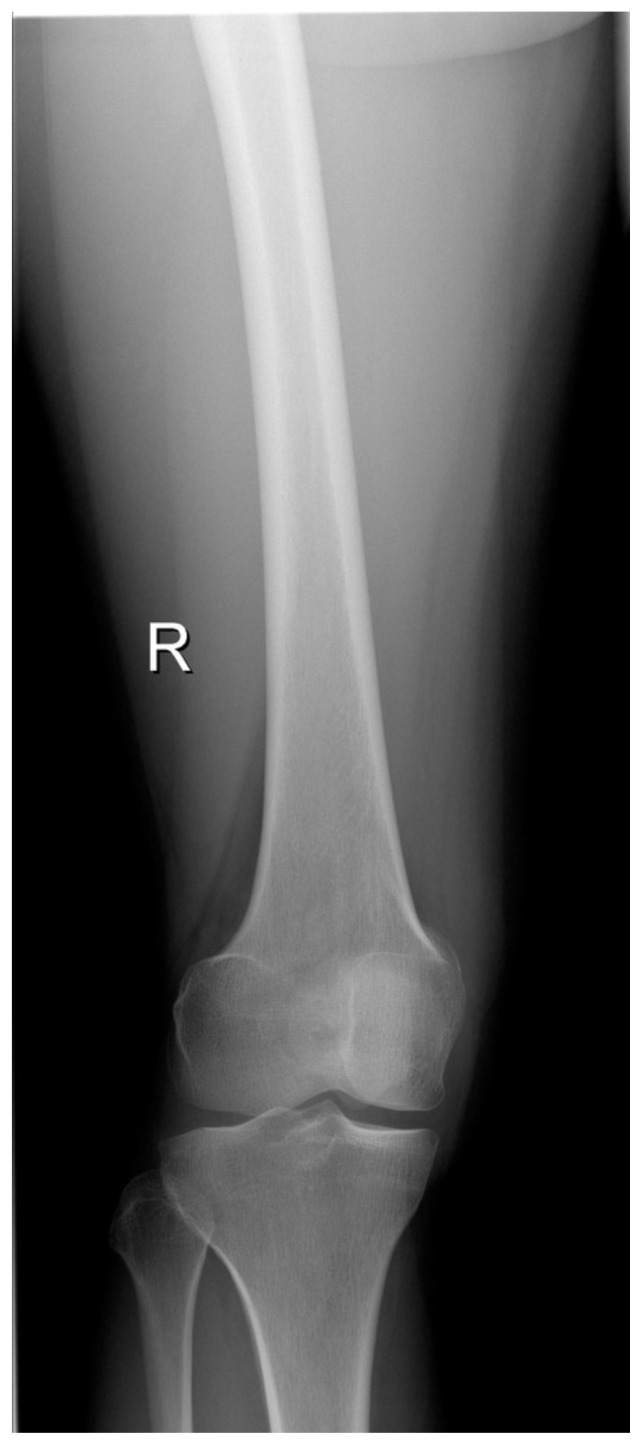
Gołofit P. Normal femur radiographs. Radiopaedia.org. https://radiopaedia.org/cases/normal-femur-radiographs-2?lang=us. Published 2019. Accessed August 13, 2019. CC BY-NC-SA 3.0.

**Figure:**
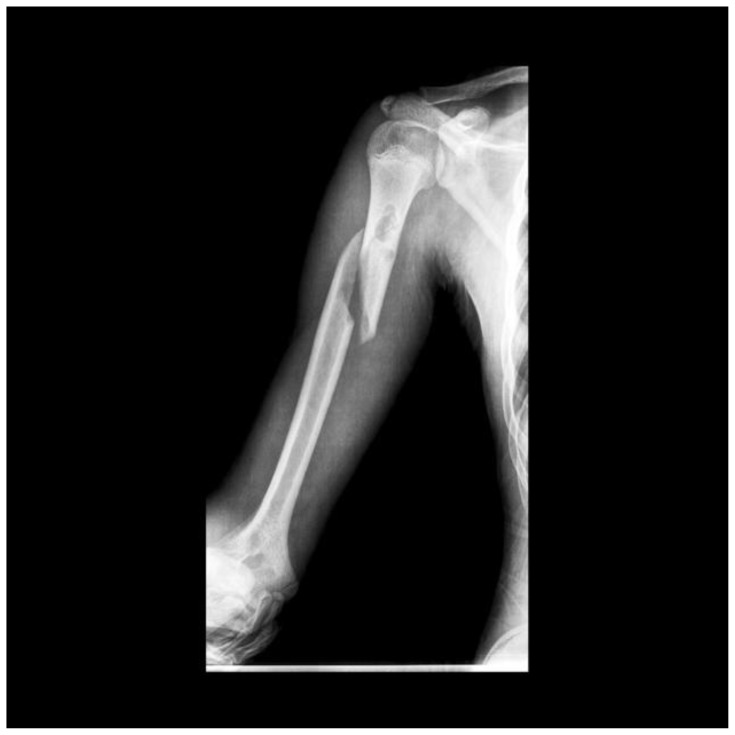
Samir B. Humeral shaft fracture. Radiopaedia.org. rID: 22063. https://radiopaedia.org/articles/humeral-shaft-fracture-1?lang=us#image_list_item_3012469. Published 2020. Accessed June 3, 2020. CC BY-NC-SA 3.0.

**Figure:**
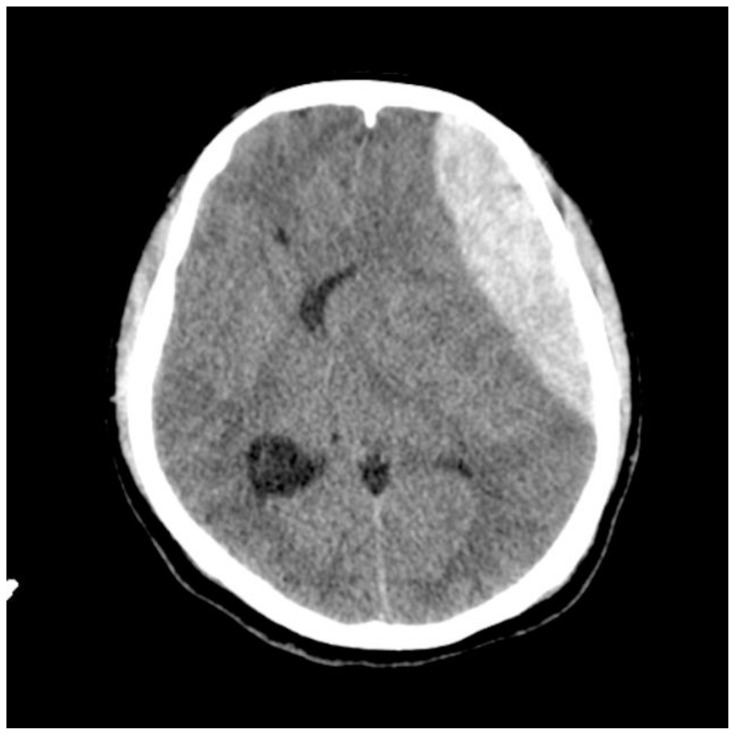
Cuete D. Epidural hematoma.Radiopaedia.org. Radiopaedia.org. rID: 29440. https://radiopaedia.org/cases/epidural-haematoma-4. Published 2019. Accessed August 28, 2019. CC BY-NC-SA 3.0.

**Figure:**
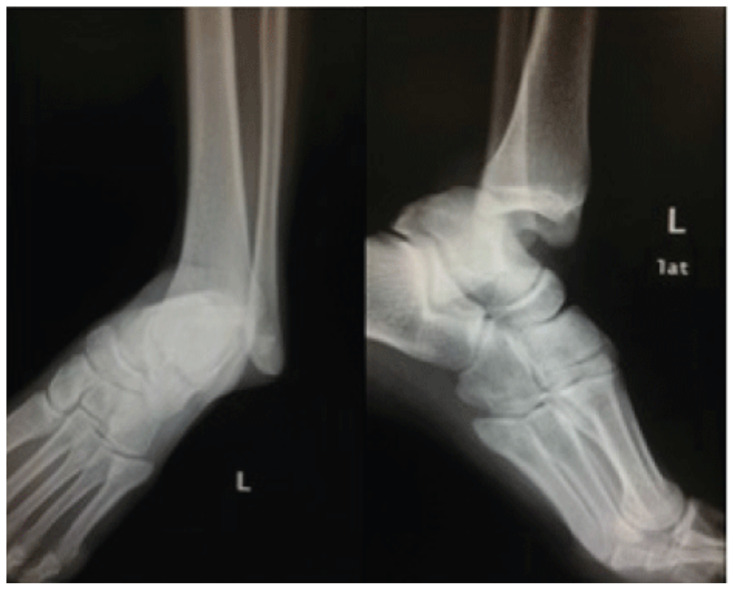
Orapin J, Sa-ngasoongsong P, Thamyongkit S. Case Report: Closed Posteromedial Dislocation of the Ankle without Medial Malleolar Fracture. *Clinical Research on Foot & Ankle*. 2016;4(3). doi:10.4172/2329-910x.1000197. Open access creative commons license.

**Figure:**
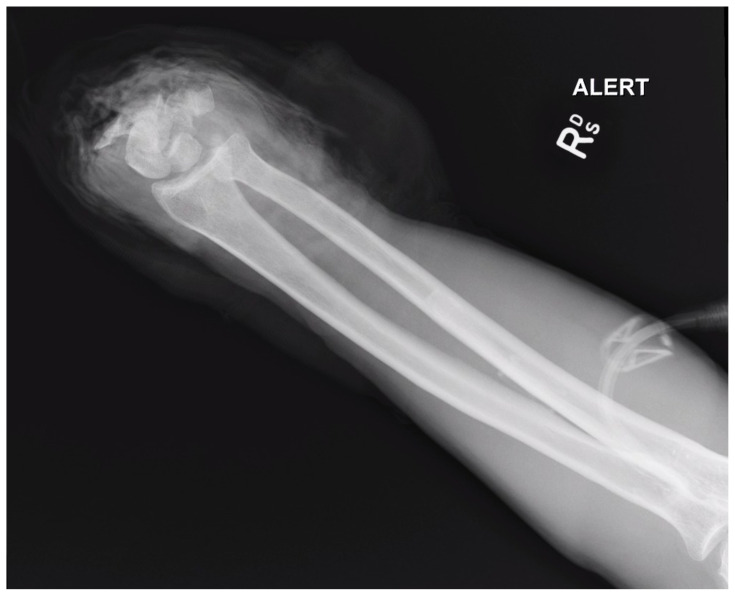
Cheng, J. Bilateral hand amputation. Radiopaedia.org. rID: 53544. https://radiopaedia.org/cases/bilateral-hand-amputation?lang=us. Published 2020. Accessed June 3, 2020. CC BY-NC-SA 3.0.

**Figure:**
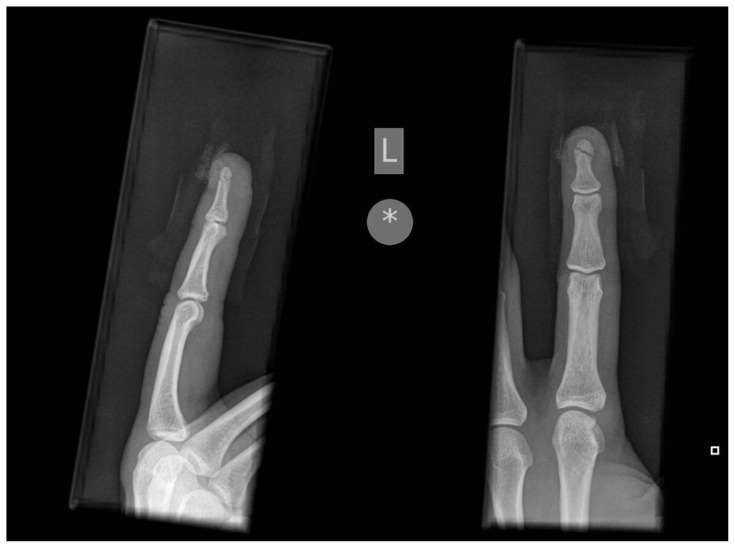
Jones J. Middle finger tuft fracture. Radiopaedia.org, rID: 6429. https://radiopaedia.org/cases/middle-finger-tuft-fracture. Published 2019. Accessed August 13, 2019. CC BY-NC-SA 3.0.

**Figure:**
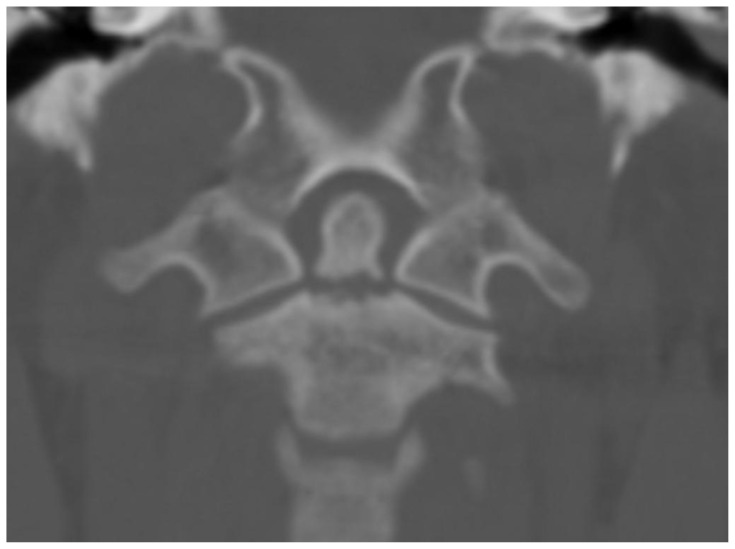
Jones J. Odontoid fracture. Radiopaedia.org. rID: 9137. https://radiopaedia.org/articles/odontoid-fracture. Published 2019. Accessed August 13, 2019. CC BY-NC-SA 3.0.

**Figure:**
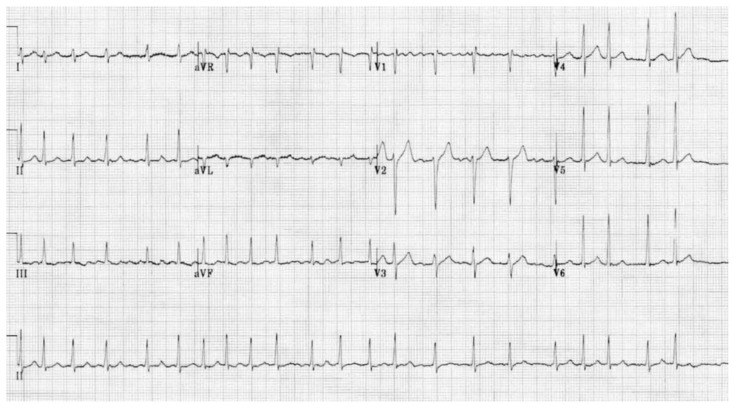
Burns E. Atrial Fibrillation. Life in the Fast Lane. https://litfl.com/atrial-fibrillation-ecg-library/. Published 2019. Accessed August 28, 2019. CC BY-NC-SA 4.0.

**Figure:**
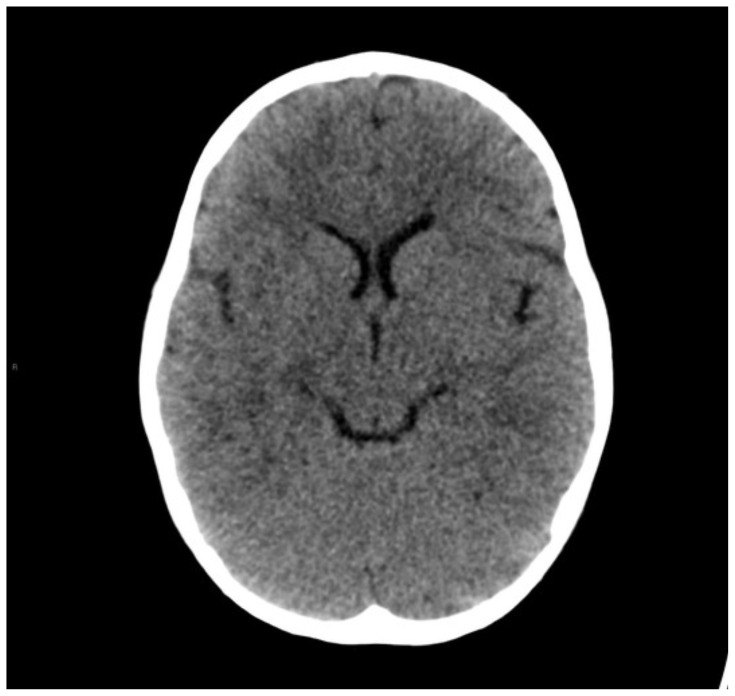
Smith D. Normal CT head (3 years old). Radiopaedia.org. rID: 53696. https://radiopaedia.org/cases/normal-ct-head-3-years-old?lang=us. Published 2019. Accessed August 13, 2019. CC BY-NC-SA 3.0.

**Figure:**
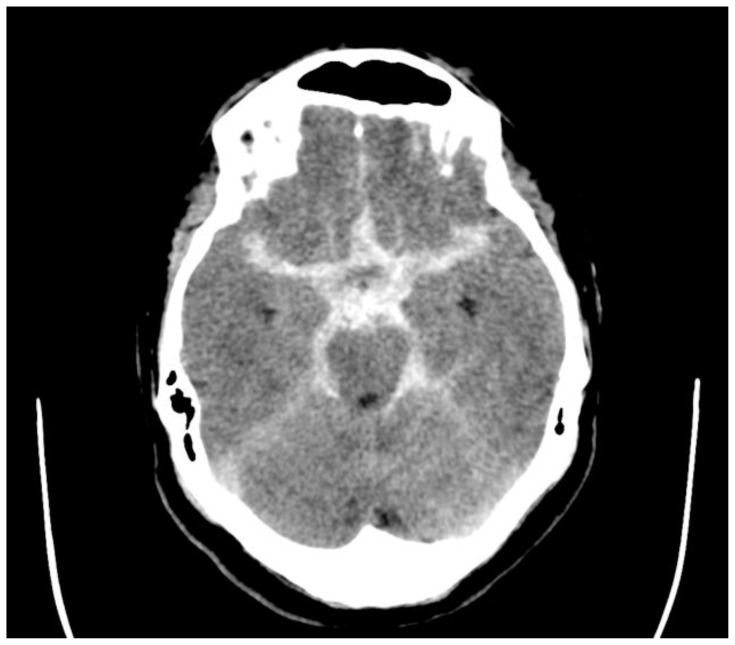
Puyo D. Subarachnoid hemorrhage. Radiopaedia.org. rID: 22377. https://radiopaedia.org/cases/subarachnoid-haemorrhage-4. Published 2019. Accessed August 28, 2019. CC BY-NC-SA 3.0.

**Figure:**
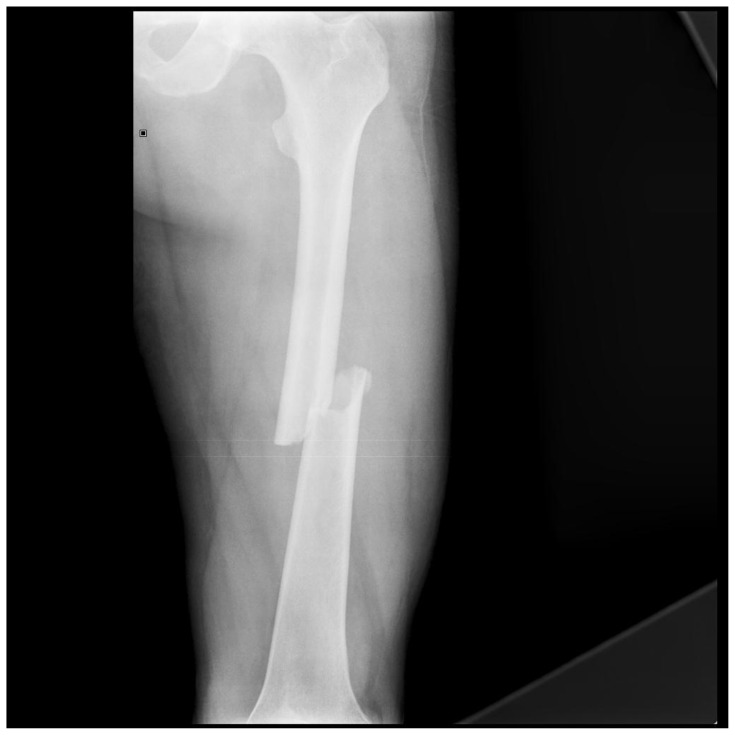
Samir, B. Femoral shaft fracture. Radiopaedia.org. rID: 22120. https://radiopaedia.org/cases/femoral-shaft-fracture-2?lang=us. Published 2020. Accessed June 3, 2020. CC BY-NC-SA 3.0.

**Figure:**
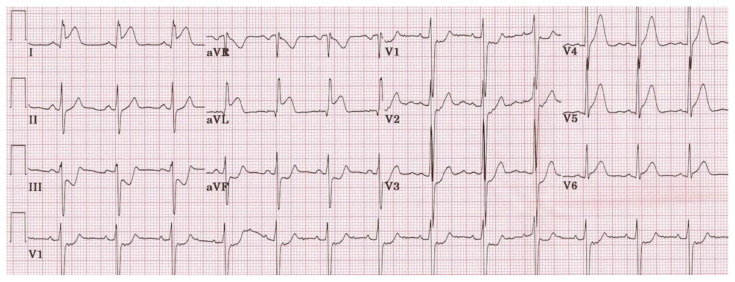
Burns E. Lateral STEMI ECG changes. Life in the Fast Lane. https://litfl.com/lateral-stemi-ecg-library/. Published 2019. Accessed August 28, 2019. CC BY-NC-SA 4.0

**Figure:**
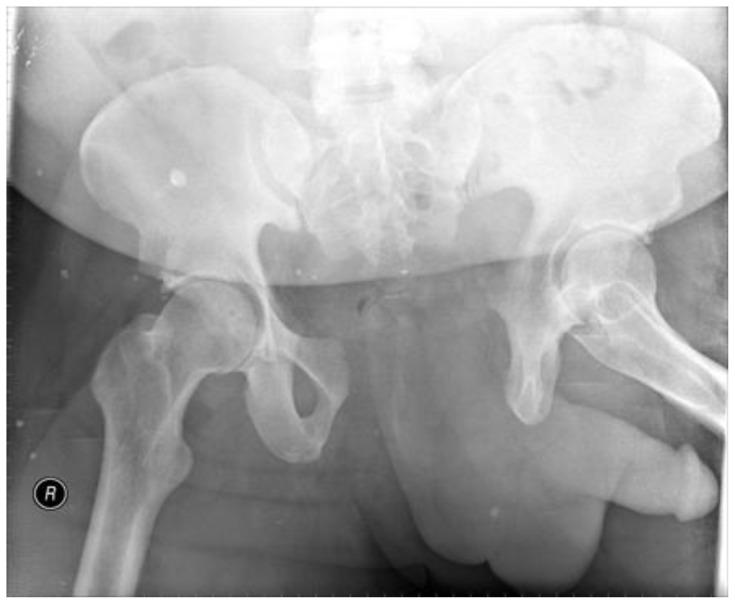
Harvey H. Pelvic fractures. Radiopaedia.org. rID: 6611. https://radiopaedia.org/articles/pelvic-fractures. Published 2019. Accessed August 13, 2019. CC BY-NC-SA 3.0.

**Figure:**
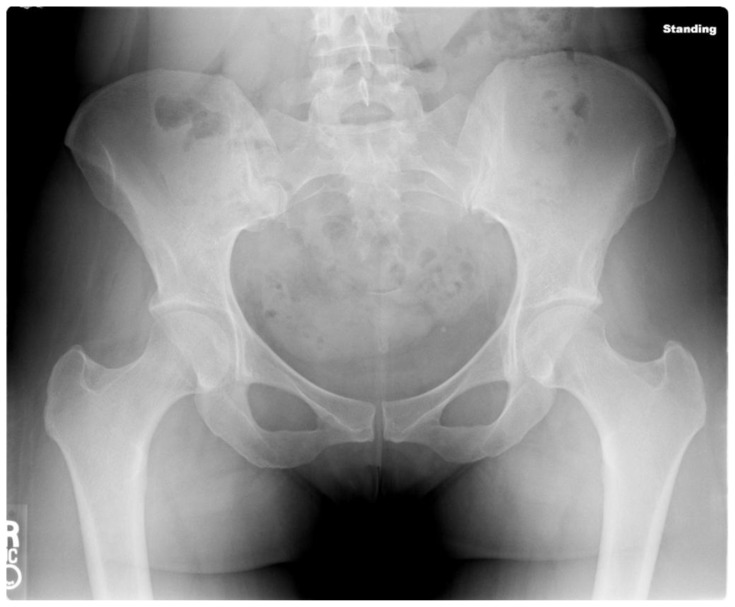
Jones J. Normal pelvis x-ray AP. Radiopaedia.org. rID: 36147. https://radiopaedia.org/cases/normal-pelvis-x-ray-ap. Published 2019. Accessed August 13, 2019. CC BY-NC-SA 3.0.

**Figure:**
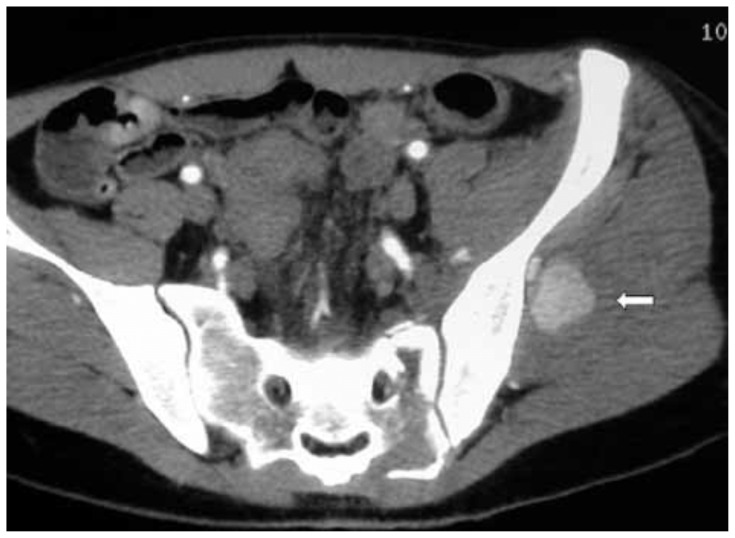
Maled I, Velez R, Lopez R, Batalla L. Pseudoaneurysm of the superior gluteal artery during iliosacral screw fixation. Acta Orthopaedica Belgica. 2007;73(4):544–547.

**Figure:**
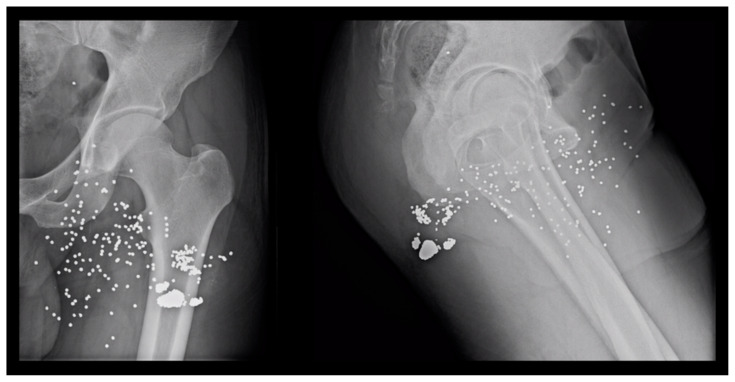
Pinotti E, Santurro L, Famularo S, Uggeri F. Surgical removal of radiolucent lodged bullet fragment 5 years after a shotgun injury. *Open Journal of Clinical and Medical Case Reports*. 2017;3. CC BY 4.0.

**Figure:**
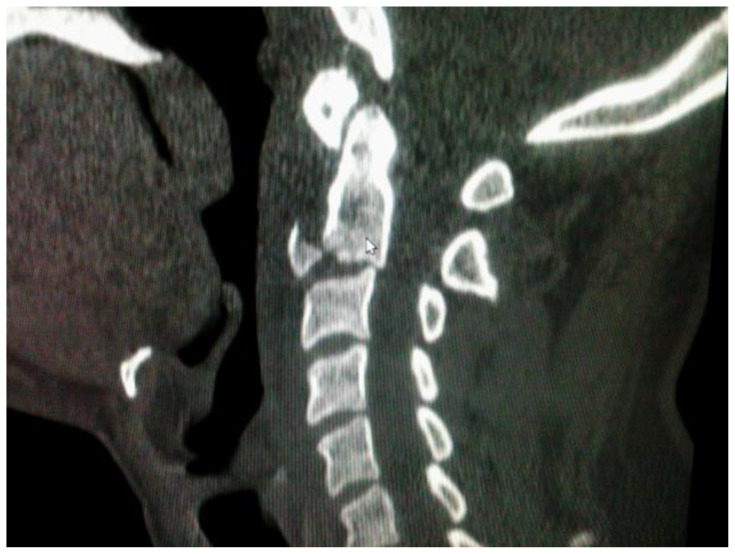
Venkatesh M.. Extension teardrop fracture. Radiopaedia.org. rID: 26327. https://radiopaedia.org/articles/extension-teardrop-fracture-1?lang=us. Published 2019. Accessed August 13, 2019. CC BY-NC-SA 3.0.

**Figure:**
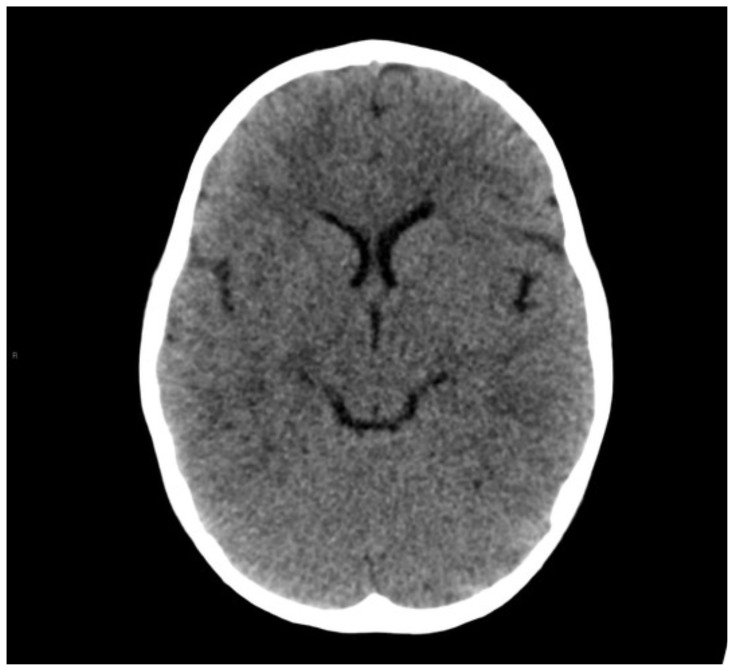
Smith D. Normal CT head (3 years old). Radiopaedia.org. rID: 53696. https://radiopaedia.org/cases/normal-ct-head-3-years-old?lang=us. Published 2019. Accessed August 13, 2019. CC BY-NC-SA 3.0.

**Figure:**
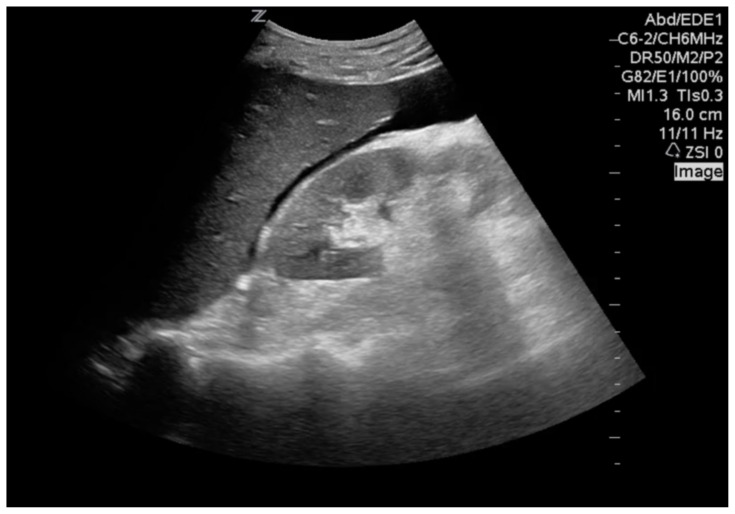
Brick C. Clinical Ultrasonography 101: Where Right Upper Quadrant Scans Go Wrong - CanadiEM. [online] CanadiEM. Available at: https://canadiem.org/right-upper-quadrant-scans-go-wrong/. Published 2019. Accessed August 13, 2019. CC BY-NC-SA 4.0.

**Figure:**
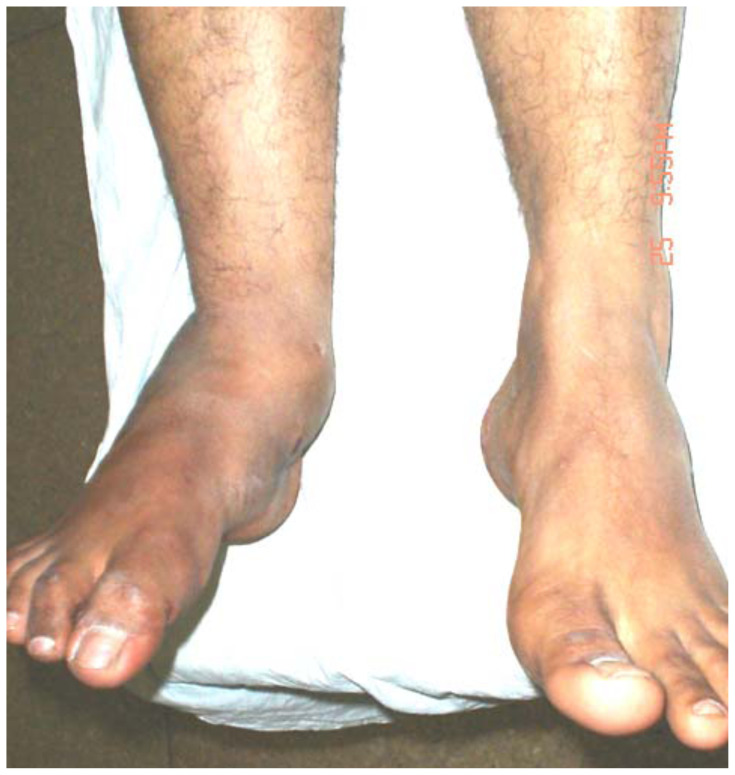
Jerome JTJ, MNAMS, Varghese M, Sankaran B, Thirumagal K. Lateral subtalar dislocation of the foot: a case report. *The Foot and Ankle Journal*. 2008;12(2). *doi: 10.3827/*faoj.2008.0112.0002. CC BY.

**Figure:**
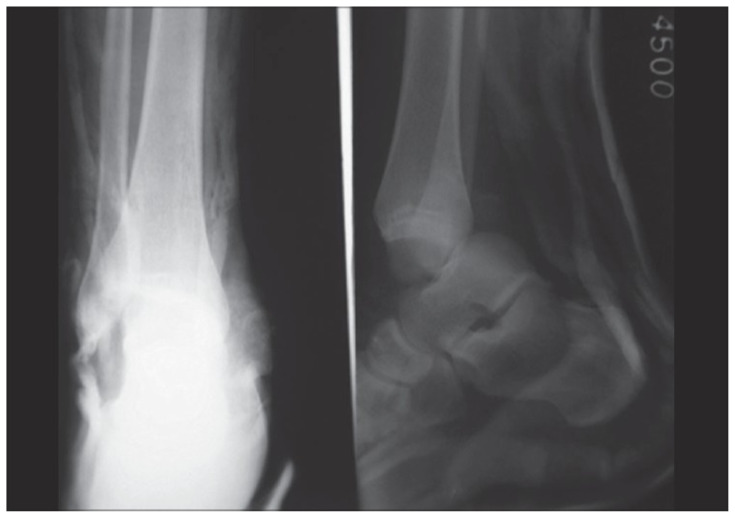
Agrawal AC, Raza HK, Haq RU. Closed posterior dislocation of the ankle without fracture. *Indian J Orthop*. 2008;42(3):360–362. doi:10.4103/0019-5413.41864. CC-BY.

**Figure:**
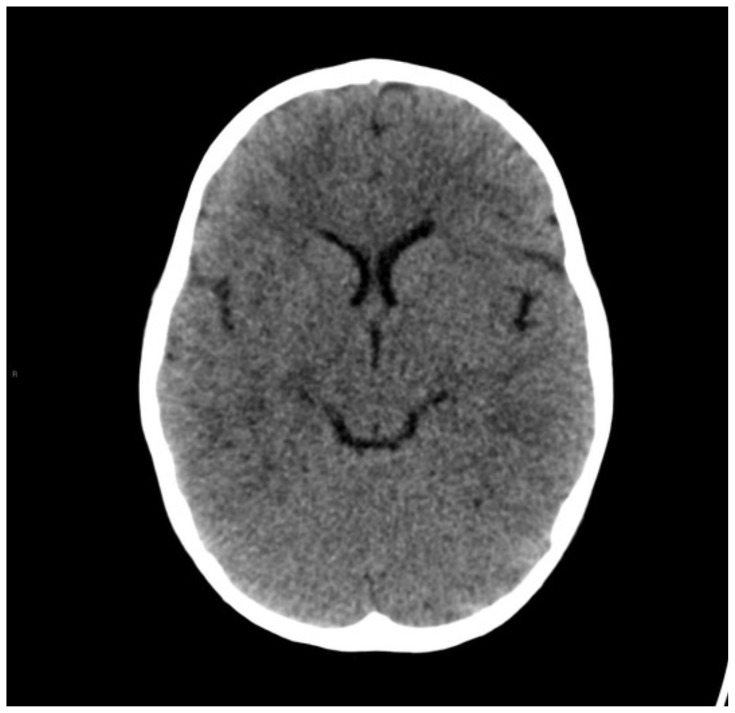
Smith D. Normal CT head (3 years old). Radiopaedia.org. rID: 53696. https://radiopaedia.org/cases/normal-ct-head-3-years-old?lang=us. Published 2019. Accessed August 13, 2019. CC BY-NC-SA 3.0.

**Figure:**
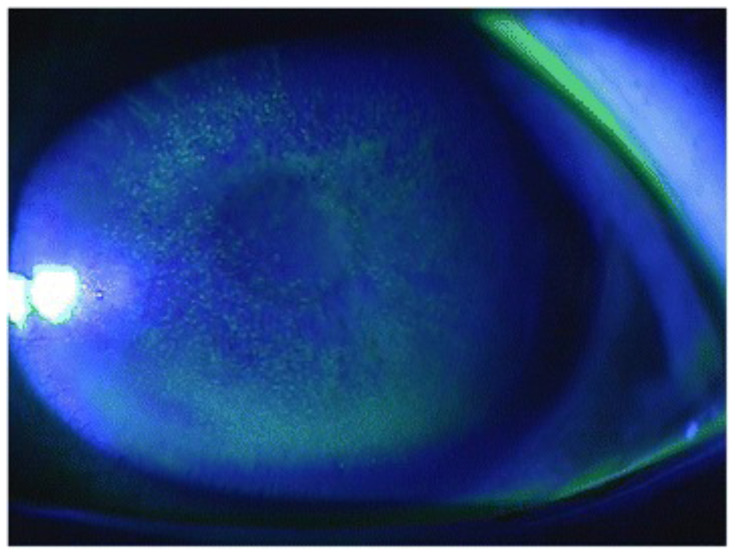
Kwon DH, Moon JD, Park WJ, et al. Case series of keratitis in poultry abattoir workers induced by exposure to the ultraviolet disinfection lamp. *Ann Occup Environ Med*. 2016; 28:3. Published 2016 Jan 15. doi:10.1186/s40557-015-0087-7. CC BY 4.0.

**Figure:**
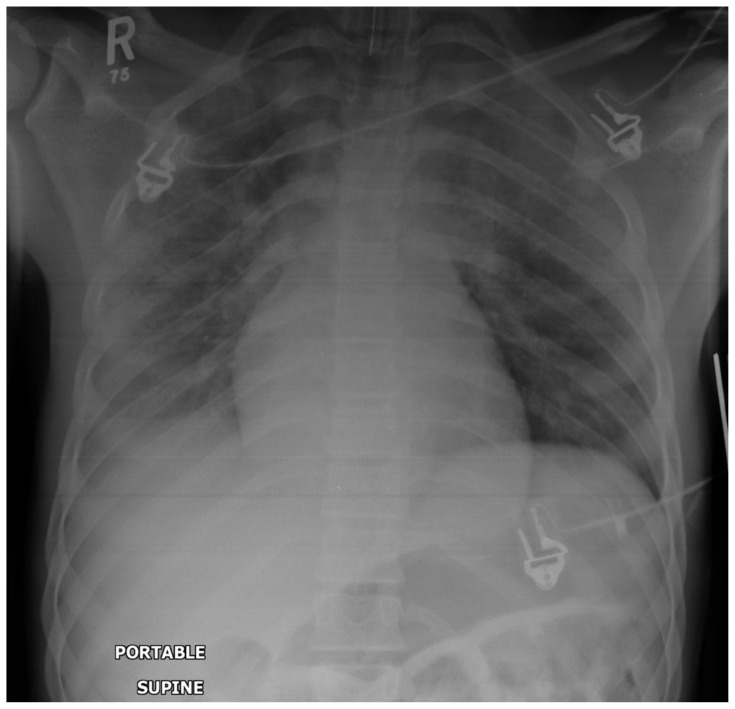
Salam, H. Pulmonary contusion. Radiopaedia.org. rID: 13270. https://radiopaedia.org/cases/pulmonary-contusion-2?lang=us. Published 2020. Accessed June 4, 2020. CC BY-NC-SA 3.0.

**Figure:**
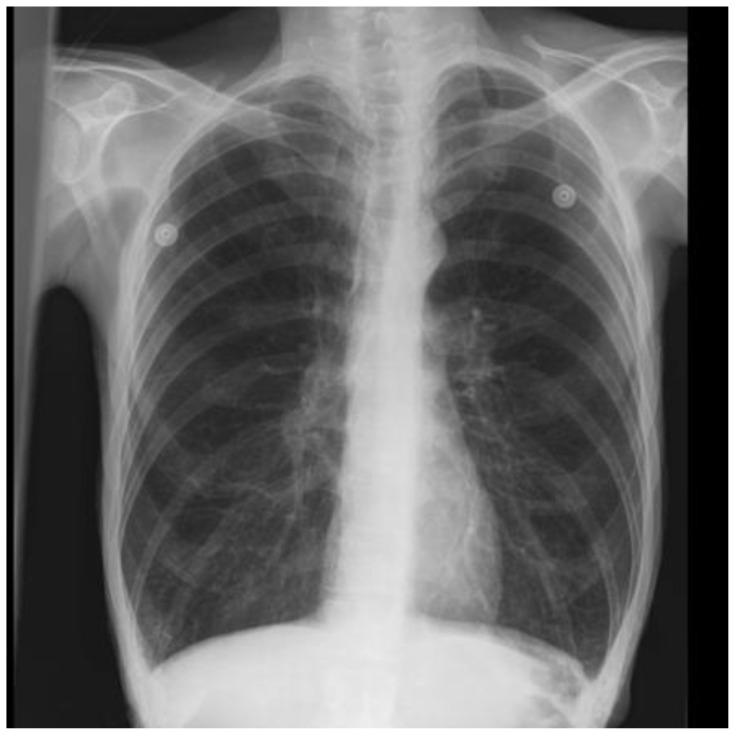
Gaillard F. Lung hyperinflation. Radiopaedia.org. rID: 10550. https://radiopaedia.org/articles/lung-hyperinflation-1?lang=us. Published 2019. Accessed August 13, 2019. CC BY-NC-SA 3.0.

**Figure:**
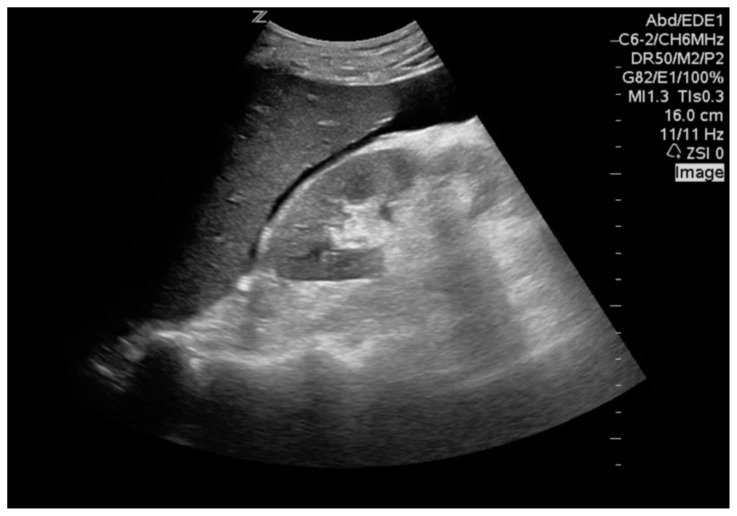
Brick C. Clinical Ultrasonography 101: Where Right Upper Quadrant Scans Go Wrong - CanadiEM. [online] CanadiEM. Available at: https://canadiem.org/right-upper-quadrant-scans-go-wrong/. Published 2019. Accessed August 13, 2019. CC BY-NC-SA 4.0.

**Figure:**
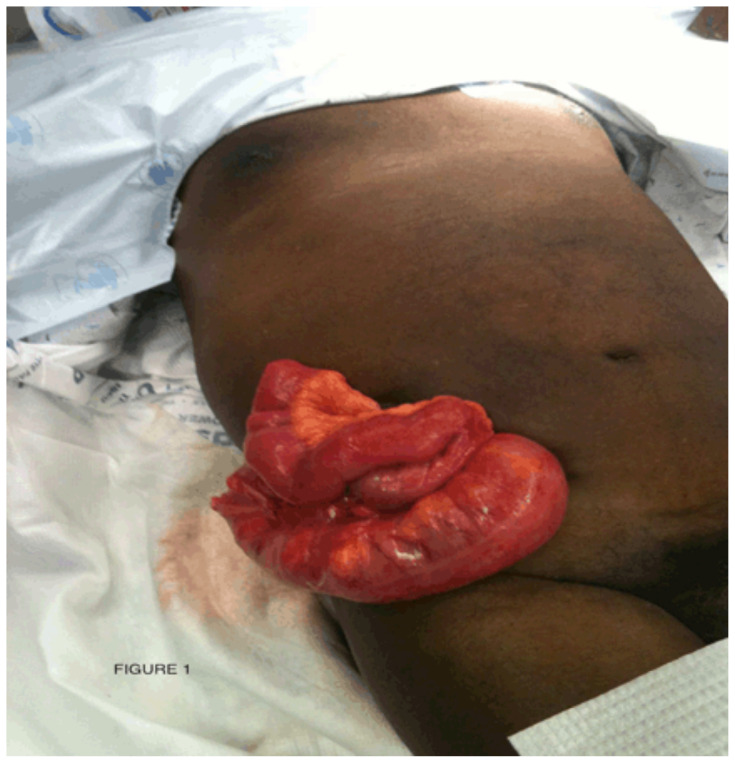
Gundlapalli VS. Recurrent evisceration from Mcburney’s incision. International Journal of Case Reports and Images 2012;3(6):54–56. CC BY 4.0.

**Figure:**
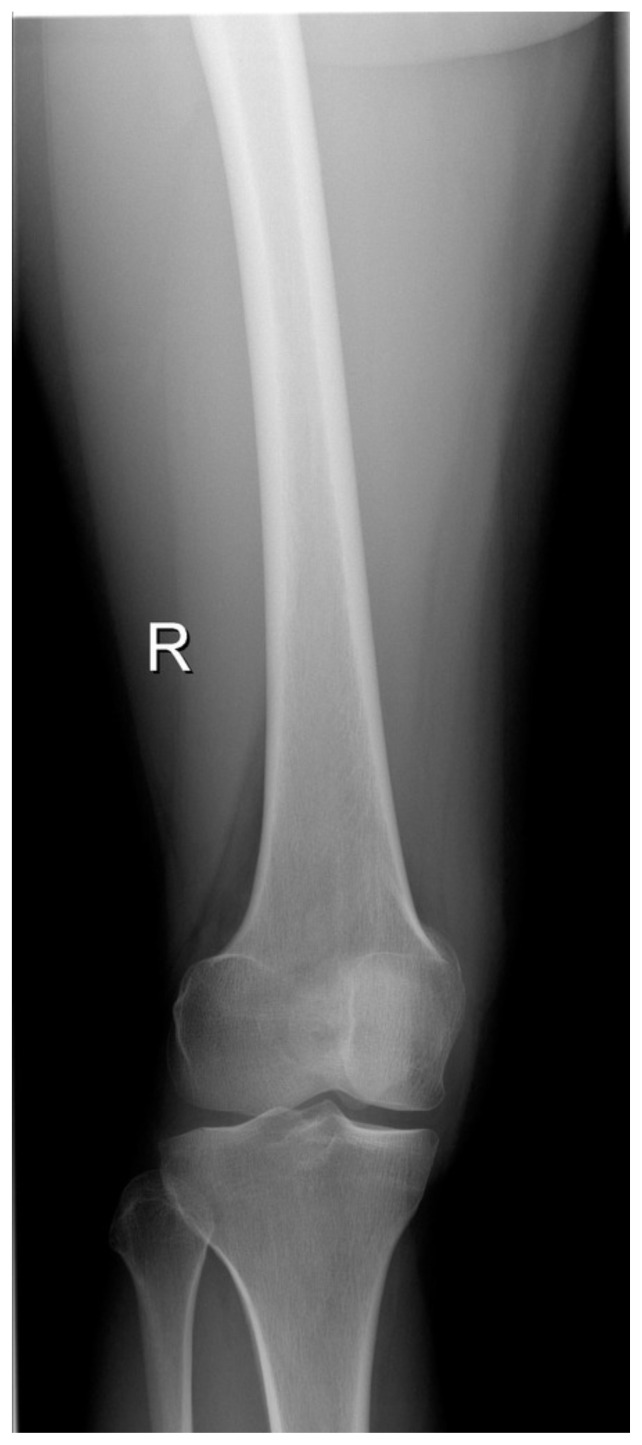
Gołofit P. Normal ://radiopaedia.org/cases/normal-femur-radiographs-2?lang=us. Published 2019. Accessed August 13, 2019. CC BY-NC-SA 3.0.

**Figure:**
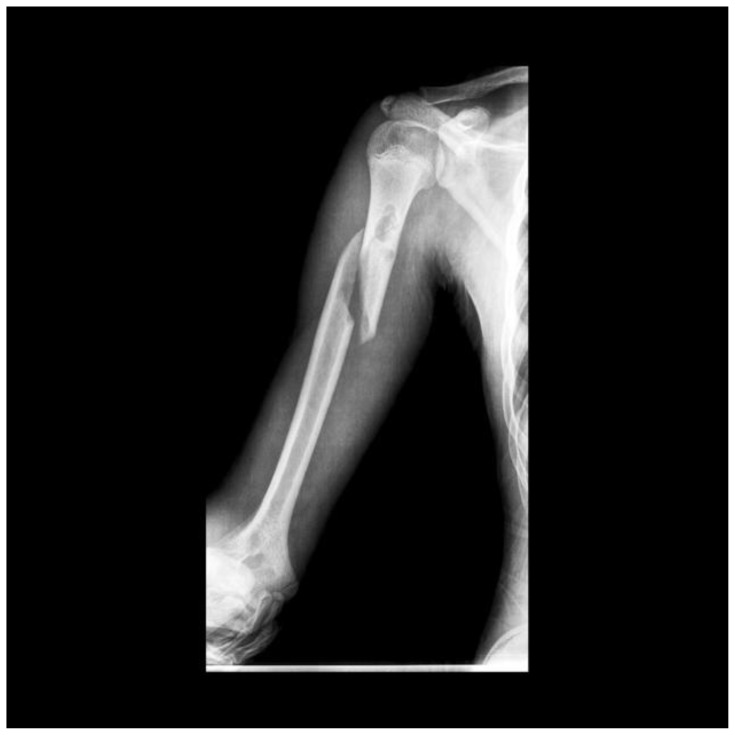
Samir B. Humeral shaft fracture. Radiopaedia.org. rID: 22063. https://radiopaedia.org/articles/humeral-shaft-fracture-1?lang=us#image_list_item_3012469. Published 2020. Accessed June 3, 2020. CC BY-NC-SA 3.0.

**Figure:**
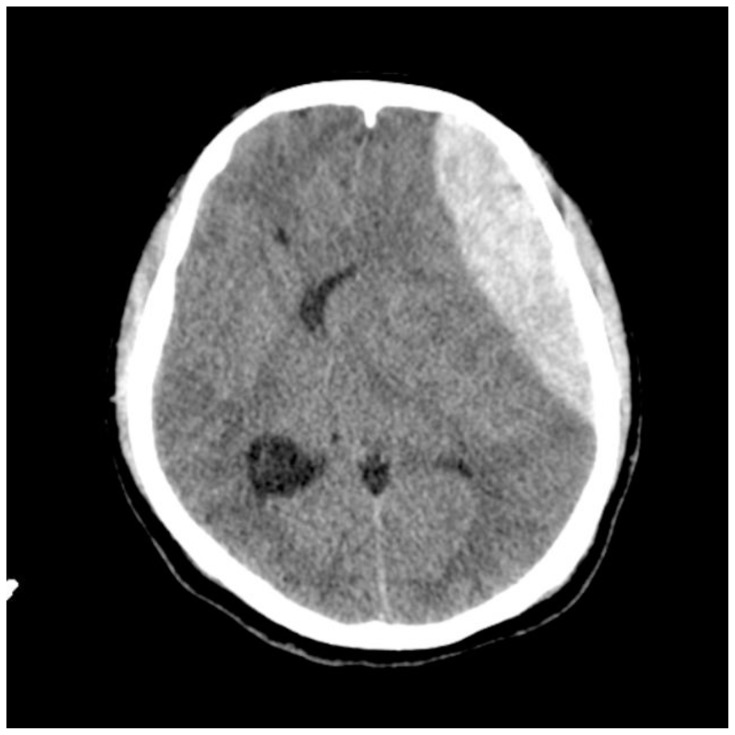
Cuete D. Epidural hematoma.Radiopaedia.org. Radiopaedia.org. rID: 29440. https://radiopaedia.org/cases/epidural-haematoma-4. Published 2019. Accessed August 28, 2019. CC BY-NC-SA 3.0.

**Figure:**
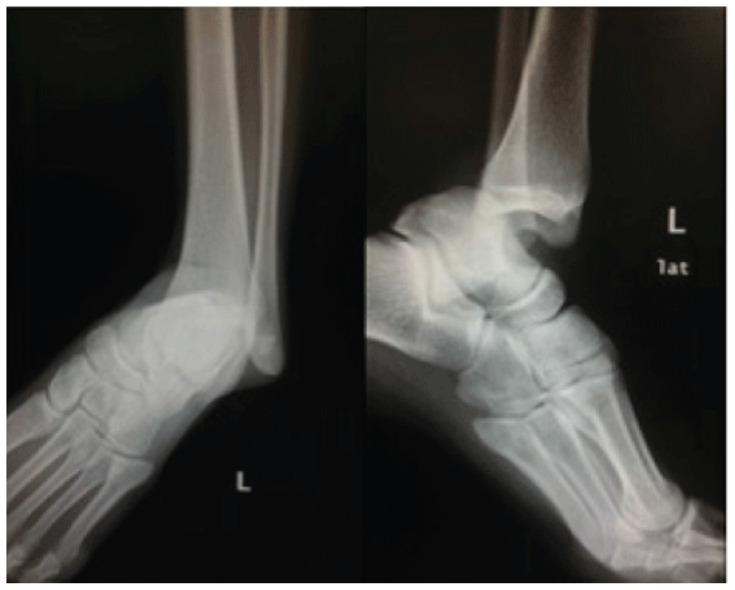
Orapin J, Sa-ngasoongsong P, Thamyongkit S. Case Report: Closed Posteromedial Dislocation of the Ankle without Medial Malleolar Fracture. *Clinical Research on Foot & Ankle*. 2016;4(3). doi:10.4172/2329-910x.1000197. Open access creative commons license.

**Figure:**
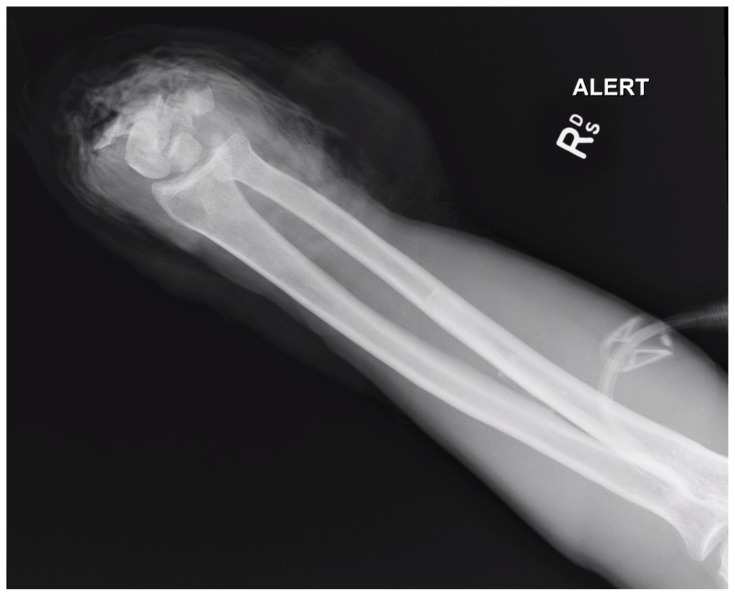
Cheng, J. Bilateral hand amputation. Radiopaedia.org. rID: 53544. https://radiopaedia.org/cases/bilateral-hand-amputation?lang=us. Published 2020. Accessed June 3, 2020. CC BY-NC-SA 3.0.

**Figure:**
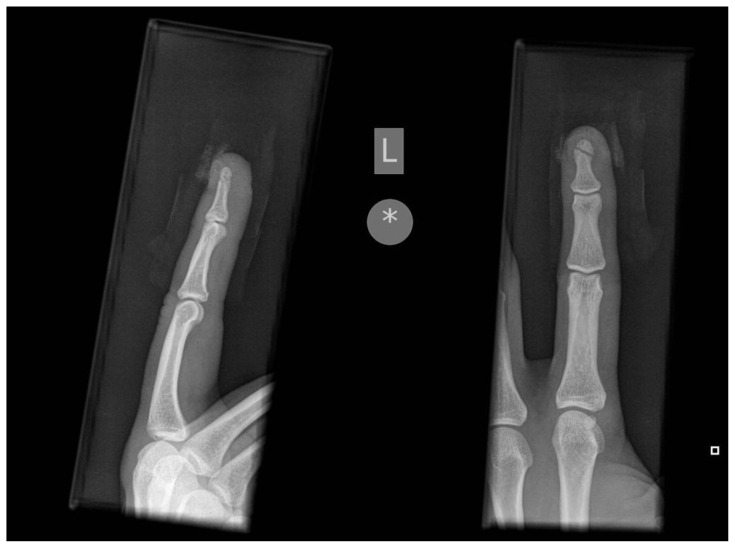
Jones J. Middle finger tuft fracture. Radiopaedia.org, rID: 6429. https://radiopaedia.org/cases/middle-finger-tuft-fracture. Published 2019. Accessed August 13, 2019. CC BY-NC-SA 3.0.

**Figure:**
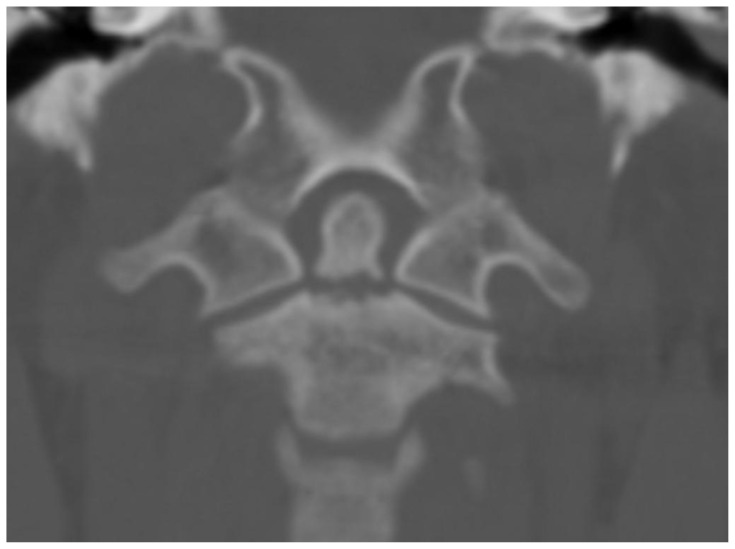
Jones J. Odontoid fracture. Radiopaedia.org. rID: 9137. https://radiopaedia.org/articles/odontoid-fracture. Published 2019. Accessed August 13, 2019. CC BY-NC-SA 3.0.

**Figure:**
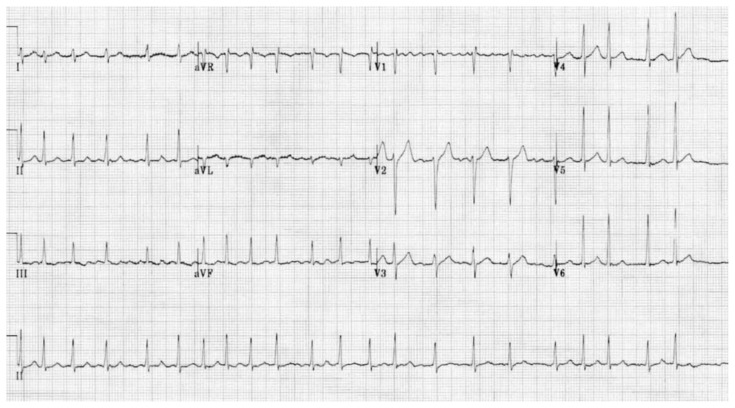
Burns E. Atrial Fibrillation. Life in the Fast Lane. https://litfl.com/atrial-fibrillation-ecg-library/. Published 2019. Accessed August 28, 2019. CC BY-NC-SA 4.0.

**Figure:**
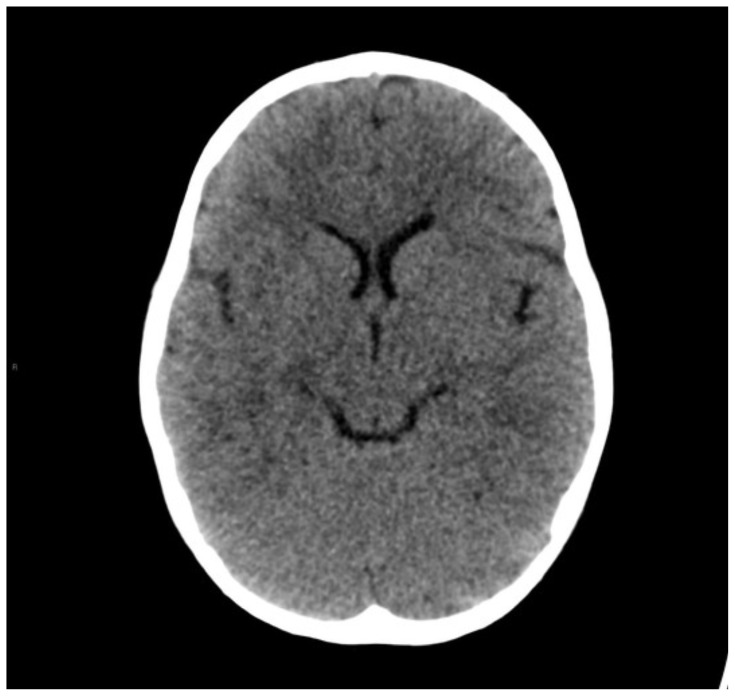
Smith D. Normal CT head (3 years old). Radiopaedia.org. rID: 53696. https://radiopaedia.org/cases/normal-ct-head-3-years-old?lang=us. Published 2019. Accessed August 13, 2019. CC BY-NC-SA 3.0.

**Figure:**
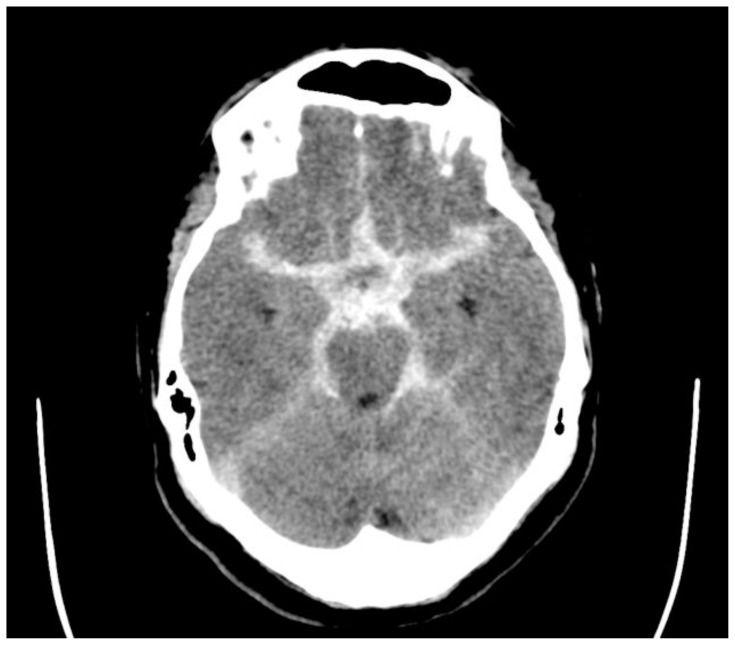
Puyo D. Subarachnoid hemorrhage. Radiopaedia.org. rID: 22377. https://radiopaedia.org/cases/subarachnoid-haemorrhage-4. Published 2019. Accessed August 28, 2019. CC BY-NC-SA 3.0.

**Figure:**
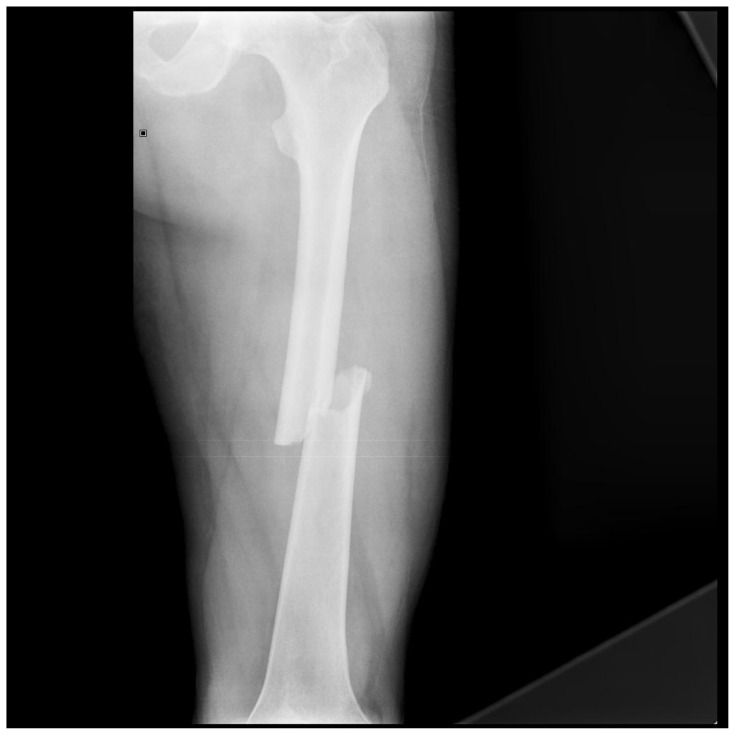
Samir, B. Femoral shaft fracture. Radiopaedia.org. rID: 22120. https://radiopaedia.org/cases/femoral-shaft-fracture-2?lang=us. Published 2020. Accessed June 3, 2020. CC BY-NC-SA 3.0.

**Figure:**
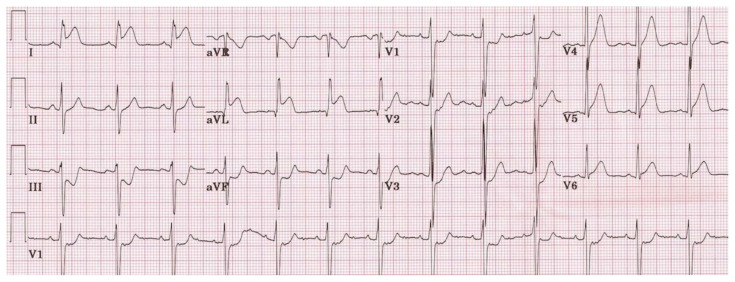
Burns E. Lateral STEMI ECG changes. Life in the Fast Lane. https://litfl.com/lateral-stemi-ecg-library/. Published 2019. Accessed August 28, 2019. CC BY-NC-SA 4.0

**Figure:**
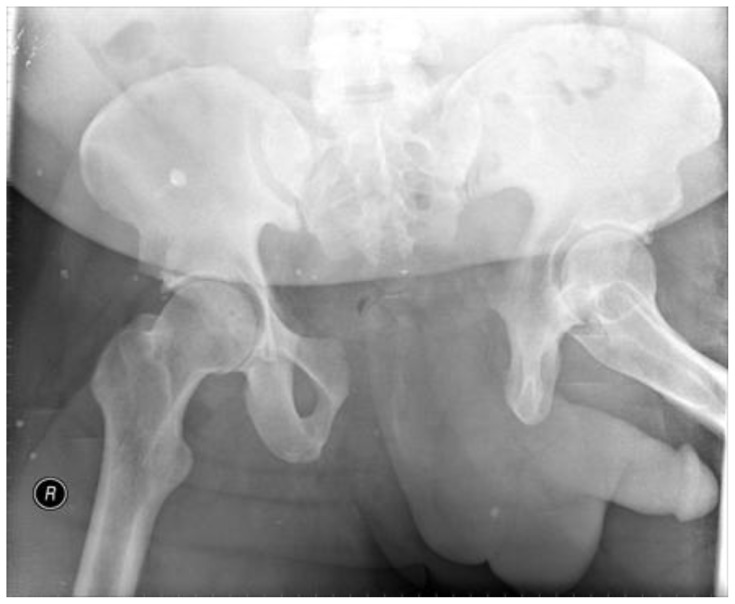
Harvey H. Pelvic fractures. Radiopaedia.org. rID: 6611. https://radiopaedia.org/articles/pelvic-fractures. Published 2019. Accessed August 13, 2019. CC BY-NC-SA 3.0.

**Figure:**
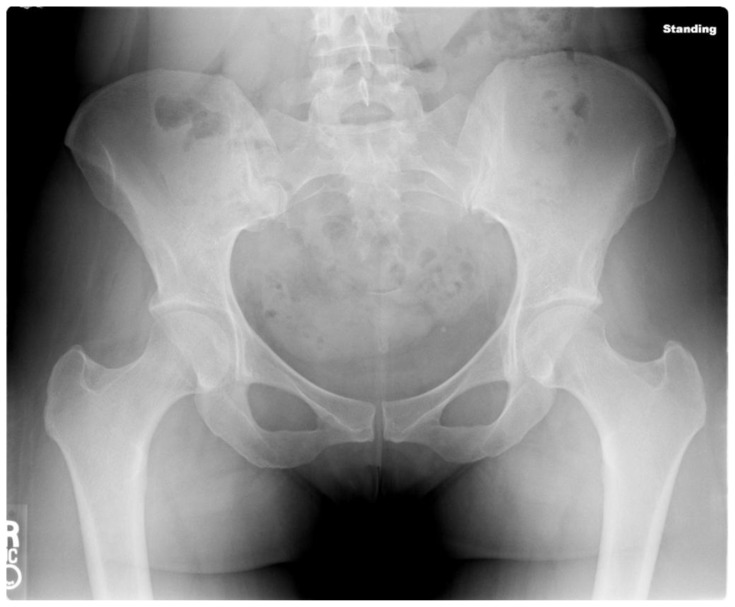
Jones J. Normal pelvis x-ray AP. Radiopaedia.org. rID: 36147. https://radiopaedia.org/cases/normal-pelvis-x-ray-ap. Published 2019. Accessed August 13, 2019. CC BY-NC-SA 3.0.

**Figure:**
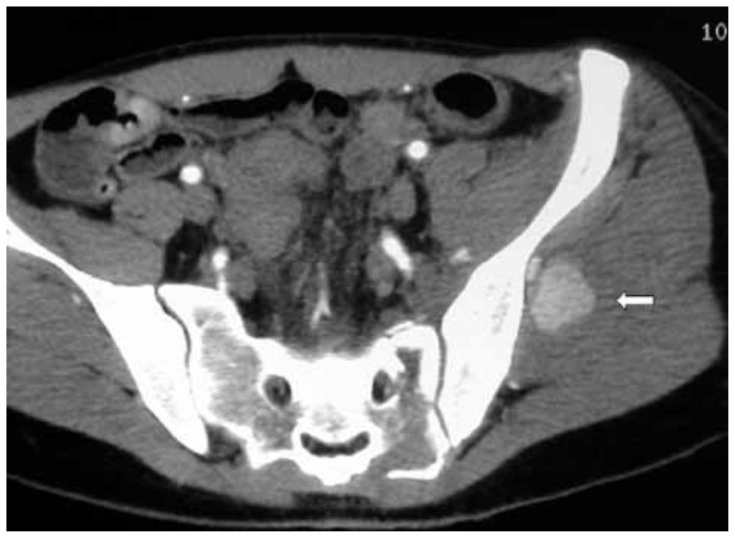
Maled I, Velez R, Lopez R, Batalla L. Pseudoaneurysm of the superior gluteal artery during iliosacral screw fixation. Acta Orthopaedica Belgica. 2007;73(4):544–547.

**Figure:**
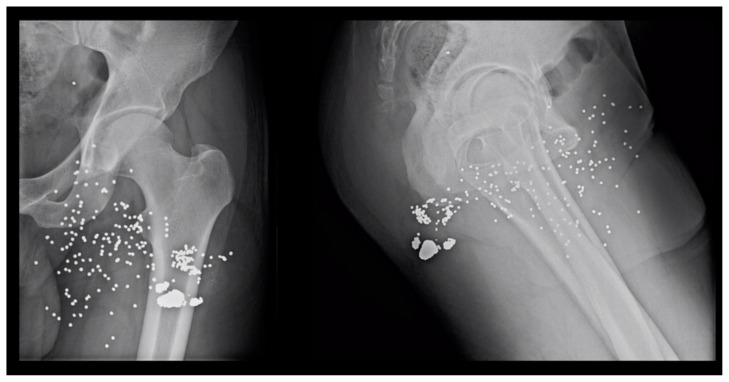
Pinotti E, Santurro L, Famularo S, Uggeri F. Surgical removal of radiolucent lodged bullet fragment 5 years after a shotgun injury. *Open Journal of Clinical and Medical Case Reports*. 2017;3. CC BY 4.0.

**Figure:**
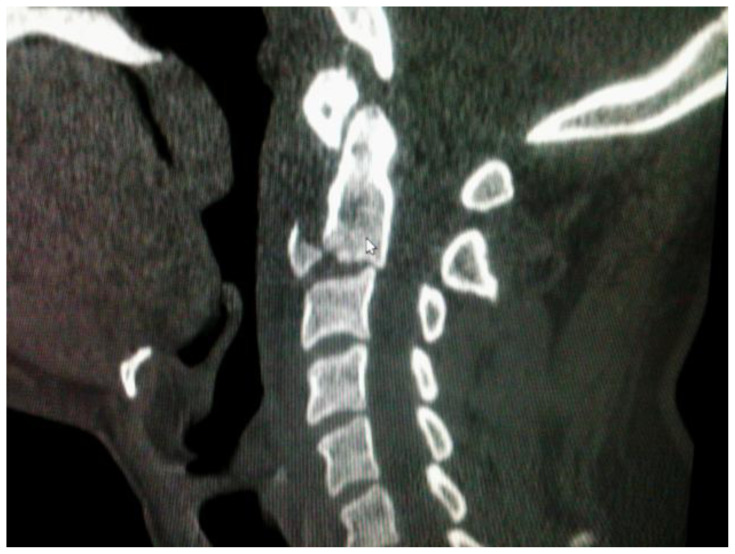
Venkatesh M.. Extension teardrop fracture. Radiopaedia.org. rID: 26327. https://radiopaedia.org/articles/extension-teardrop-fracture-1?lang=us. Published 2019. Accessed August 13, 2019. CC BY-NC-SA 3.0.

**Figure:**
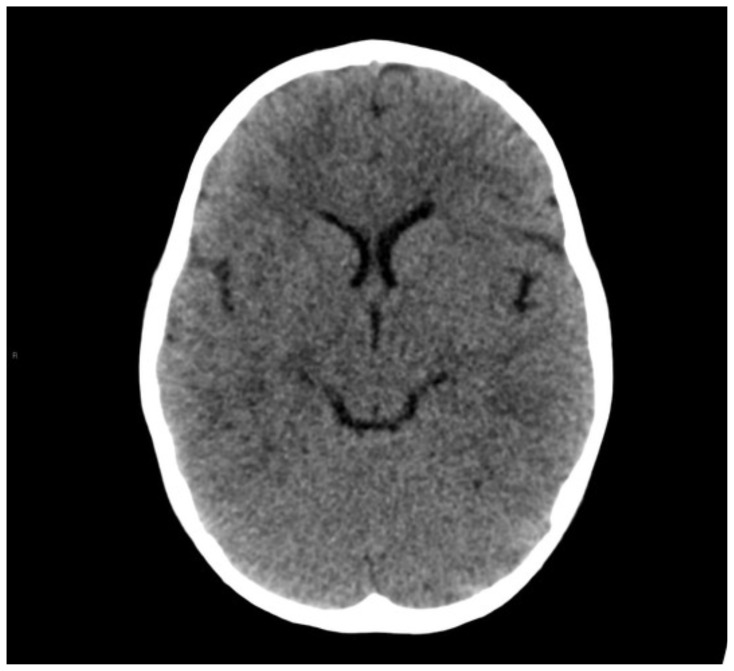
Smith D. Normal CT head (3 years old). Radiopaedia.org. rID: 53696. https://radiopaedia.org/cases/normal-ct-head-3-years-old?lang=us. Published 2019. Accessed August 13, 2019. CC BY-NC-SA 3.0.

**Figure:**
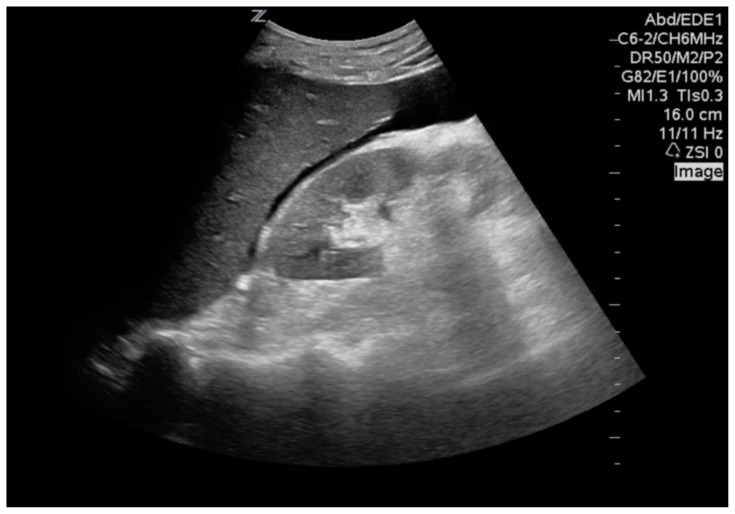
Brick C. Clinical Ultrasonography 101: Where Right Upper Quadrant Scans Go Wrong - CanadiEM. [online] CanadiEM. Available at: https://canadiem.org/right-upper-quadrant-scans-go-wrong/. Published 2019. Accessed August 13, 2019. CC BY-NC-SA 4.0.

**Figure:**
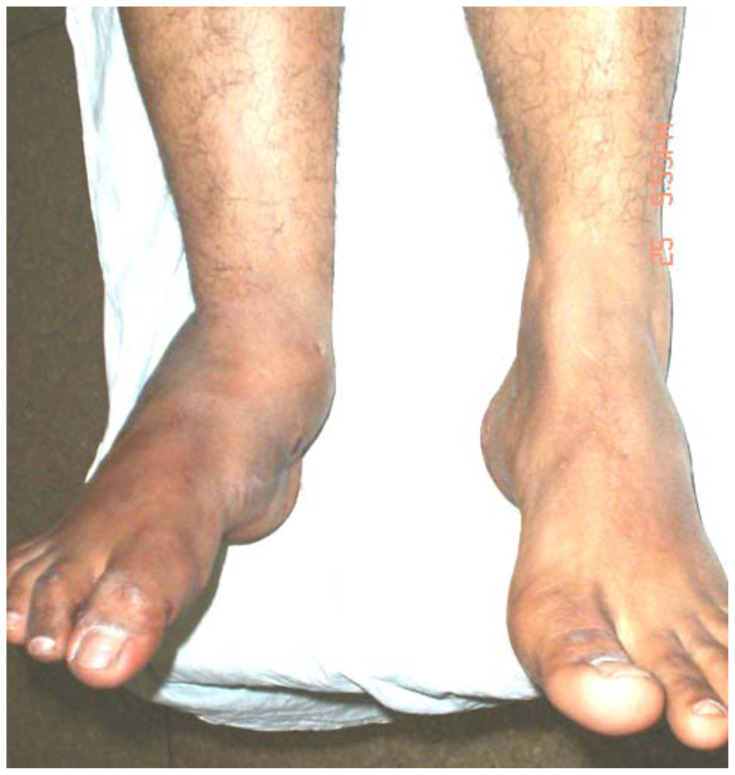
Jerome JTJ, MNAMS, Varghese M, Sankaran B, Thirumagal K. Lateral subtalar dislocation of the foot: a case report. *The Foot and Ankle Journal*. 2008;12(2). *doi:10.3827/*faoj.2008.0112.0002.CCBY.

**Figure:**
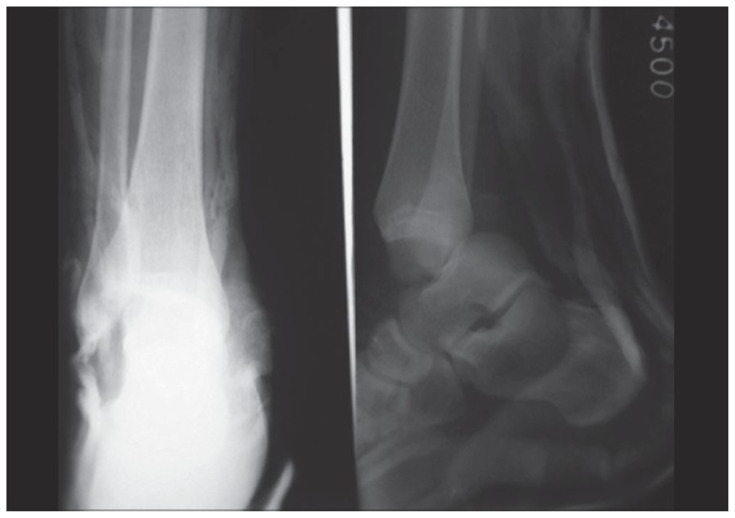
Agrawal AC, Raza HK, Haq RU. Closed posterior dislocation of the ankle without fracture. *Indian J Orthop*. 2008;42(3):360–362. doi:10.4103/0019-5413.41864.CC-BY.

**Figure:**
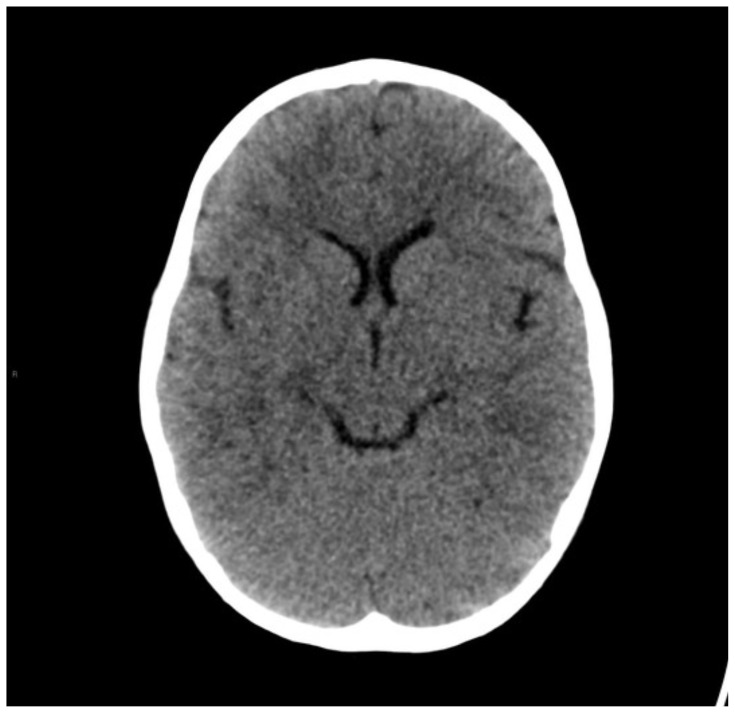
Smith D. Normal CT head (3 years old). Radiopaedia.org. rID: 53696. https://radiopaedia.org/cases/normal-ct-head-3-years-old?lang=us. Published 2019. Accessed August 13, 2019. CC BY-NC-SA 3.0.

**Figure:**
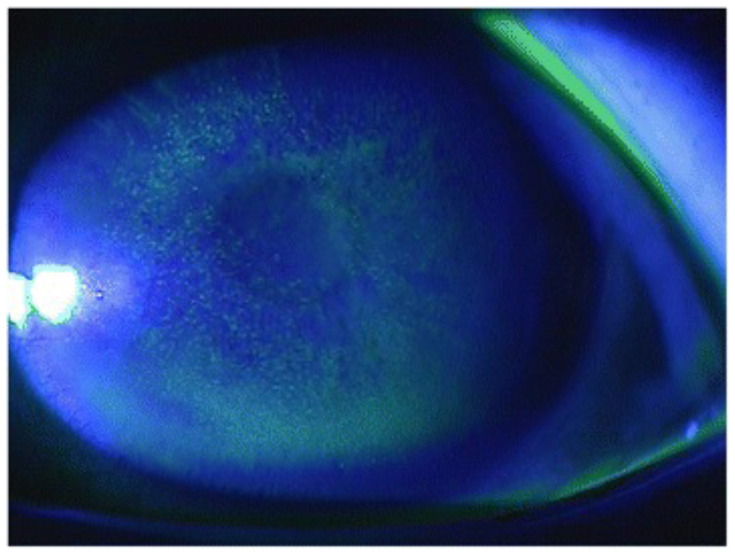
Kwon DH, Moon JD, Park WJ, et al. Case series of keratitis in poultry abattoir workers induced by exposure to the ultraviolet disinfection lamp. *Ann Occup Environ Med*. 2016; 28:3. Published 2016 Jan 15. doi:10.1186/s40557-015-0087-7. CC BY 4.0.

**Figure:**
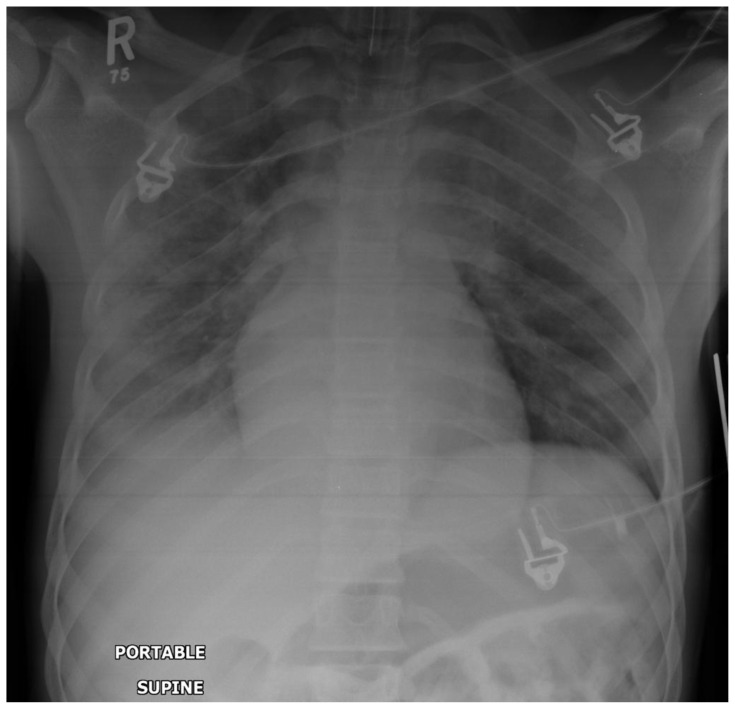
Salam, H. Pulmonary contusion. Radiopaedia.org. rID: 13270. https://radiopaedia.org/cases/pulmonary-contusion-2?lang=us. Published 2020. Accessed June 4, 2020. CC BY-NC-SA 3.0.

**Figure:**
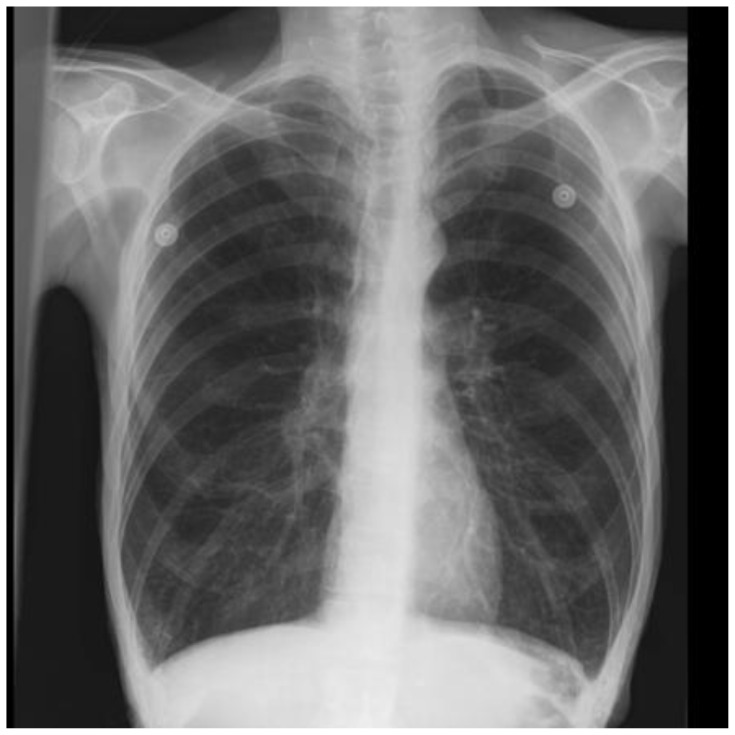
Gaillard F. Lung hyperinflation. Radiopaedia.org. rID: 10550. https://radiopaedia.org/articles/lung-hyperinflation-1?lang=us. Published 2019. Accessed August 13, 2019. CC BY-NC-SA 3.0.

**Figure:**
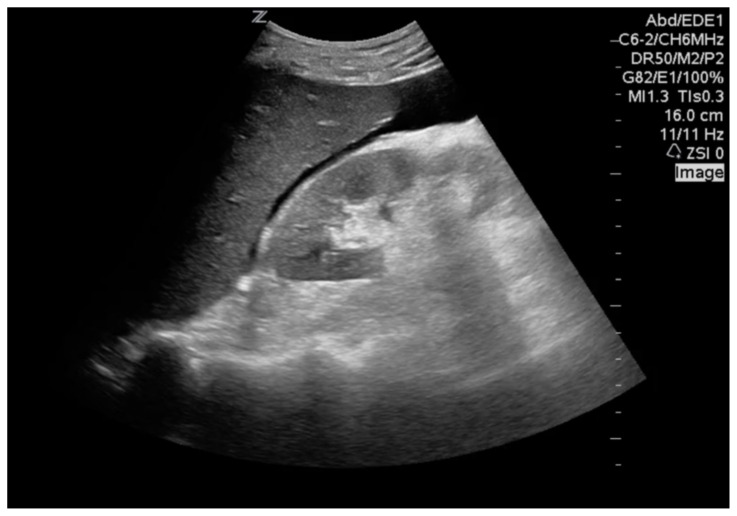
Brick C. Clinical Ultrasonography 101: Where Right Upper Quadrant Scans Go Wrong - CanadiEM. [online] CanadiEM. Available at: https://canadiem.org/right-upper-quadrant-scans-go-wrong/. Published 2019. Accessed August 13, 2019. CC BY-NC-SA 4.0.

**Figure:**
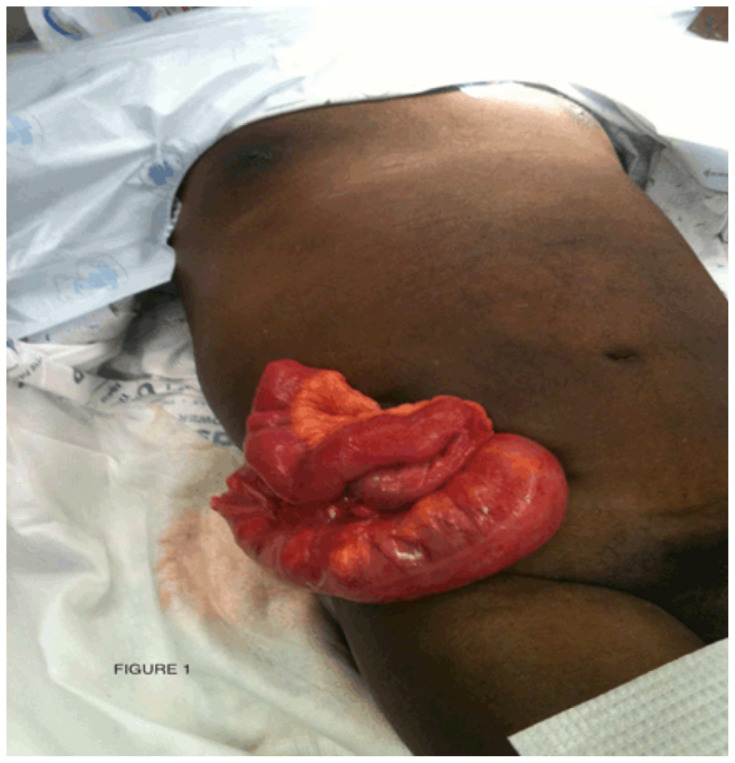
Gundlapalli VS. Recurrent evisceration from Mcburney’s incision. International Journal of Case Reports and Images 2012;3(6):54–56. CC BY 4.0.

## SMALL GROUP MATERIALS

### Appendix B: Small Group Application Exercise (sGAE) Cases for Learners

#### Learner responsible content (LRC)

Learners should read at least the first reference; however, all references are recommended for pre-reading.

1. Coti, P. Mass casualty incident triage. WikEM. Accessed 07/23/2019. https://www.wikem.org/wiki/Mass_casualty_incident_triage.
2. Culley JM, Svendsen E. A review of the literature on the validity of mass casualty triage systems with a focus on chemical exposures. *American Journal of Disaster Medicine*. 2014;9(2):137–150. doi:10.5055/ajdm.2014.0150.
3. Model uniform core criteria for mass casualty triage. Disaster Med Public Health Prep. 2011;5(2):125–8.

#### Cases with Answers

The following cases were placed on an individual page. The questions (left half of the page) were visible for the learners to view while the answers (right half of the page) were covered with scratch-off stickers. We used bulk scratch-off stickers purchased online.^10^ The learners and proctor should read the question and then learners state their expected answer. The proctor can guide the learner if needed. The learner then scratches off the sticker to reveal the answer. They can then move on to the next question below the previous question. If imaging is needed, the learners then have to transport the patient to the imaging/lab station, request the needed images/labs, and finally return all of the materials to their station to continue answering the questions associated with that patient. Once diagnosis and treatment are determined, the patient can be discharged, admitted, or sent to the OR. The proctors should be given a copy of all of the cases with uncovered answers for reference (Appendix F). The instructions and timeline should also be given to all proctors (Appendix C). Depending on time for the exercise, all of the cases could be reviewed with the learners after the exercise.

#### Instructions for Use

1. Print the case document (Appendix B) starting on page 12
2. Remove the cases from the files. Each case page starts with the number corresponding to the case followed by the prompt, then the questions and answers. The answers will be covered by the “scratch-off” stickers.
3. Place all of the imaging/laboratory pages into a separate pile.
4. Optional: use paperclips to separate the image/lab papers for each case (ie, put a paperclip around the lab and imaging results for case one). Reserve the lab/image files for the proctor in charge of labs/imaging.
5. Place the “scratch-off” stickers over the answers on the cases. Each page represents one patient. These should be mixed up and reserved for the proctor in charge of the initial triage area.

#### Start Triage Handout

- **START**: simple triage and rapid treatment
  - Ability to follow directions
  - Respiratory effort
  - Pulses/perfusion
  - Mental status
- **Classes**
  - **Red** patients: immediate, critical
  - **Yellow**: delayed, serious
  - **Green**: ambulatory, minor injuries
  - **Black**: deceased/expectant
- **Use a System**
  - Walk in a clockwise or counterclockwise manner in the disaster area
  - Tag as you go
  - Use what resources you have
  - Assess how to get more resources/call for help
  - Green -> tag and tell where to go
  - Yellow/red -> tag and assist/obtain resources to assist
  - Black -> isolate from media/other patients/onlookers. Accessible to coroner and staff

#### Abbreviations Key

Abx: Antibiotics
ANC: Absolute neutrophil count
BL: Bilateral
BUN: Blood urea nitrogen
Cath: Catheter
CBC: Complete blood count
C-collar: Cervical collar
CK: Creatinine kinase
cm: Centimeter
CMP: Complete metabolic panel
CO: Carbon monoxide
CO2: Carbon dioxide
Creat: Creatinine
C-spine: Cervical spine
CT: Computed tomography
CTA: Computed tomography angiography
CTH: Computed tomography head
CXR: Chest X-ray
D&C: Dilation and curettage
Dispo: Disposition
DM: Diabetes mellitus
DP: Dorsalis pedis
EKG: Electrocardiogram
EMS: Emergency medical services
FAST: Focused assessment with sonography in trauma
GCS: Glasgow coma scale
GFR: Glomerular filtration rate
H&H: Hemoglobin and hematocrit
Hgb: Hemoglobin
HLD: Hyperlipidemia
HR: Heart rate
HTN: Hypertension
IM: Intramuscular
IR: Interventional radiology
IVF: Intravenous fluids
LOC: Loss of consciousness
LUE: Left upper extremity
MCH: Mean corpuscular hemoglobin
MCHC: Mean corpuscular hemoglobin concentration
MCV: Mean corpuscular volume
MICU: Medical intensive care unit
MPV: Mean platelet volume
Nitro: Nitroglycerin
O2: Oxygen
OB: Obstetrics
Ophtho: Ophthalmology
OR: Operating room
Ortho: Orthopedics
PLT: Platelet
POC: Point of care
PT: Posterior tibial
RBC: Red blood cells
RDW-CV: Red blood cell distribution width – coefficient of variation
RR: Respiratory rate
RUE: Right upper extremity
SICU: Surgical intensive care unit
SOB: Shortness of breath
XR: X-ray

1. 29-year-old female with foreign body in leg. She is able to ambulate with assistance from a friend. She has a tourniquet in place. It is still bleeding a lot, but her capillary refill is less than two seconds. She is breathing at 18/minute. The wound looks like it is pretty deep.
  **Table:**
  Triage zone?	Green
  Tests?	XR, CTA, Hgb
  Treatment?	Stabilize, OR
2. 30-year-old male with full body burns with large areas that appear white. Patient complaining of moderate, but not severe pain. He has soot in his oropharynx and nares and has a RR of 40.
  **Table:**
  Triage zone?	Red
  Tests?	CBC, CMP, lactate
  Treatment?	Wound care, IVF consider intubation
  Dispo?	Admit to burn
3. 44-year-old male with sharp trauma to neck. Capillary refill of four seconds and the patient is not following simple commands. The patient is dripping blood everywhere. You cannot see if it is pulsatile under the bandages.
  **Table:**
  Triage zone?	Red
  Tests?	None - surgery to OR
  Treatment?	Apply pressure, surgery
  Dispo?	Admit
4. 21-year-old male, inebriated. He is shouting “my arm hurts!” There is an obvious deformity.
  **Table:**
  Triage zone?	Green
  Tests?	XR
  Treatment?	Reduce, splint
  Dispo?	Discharge, ortho follow up
5. 64-year-old female, breathing, not responding to commands, eyes closed, nonverbal. Appears to have a large parietal hematoma.
  **Table:**
  Triage zone?	Red
  Tests?	CTH
  Treatment?	Neurosurgery
  Dispo?	Admit to SICU
6. 32-year-old male, not ambulatory, bleeding from proximal right thigh. He has a tourniquet in place. When the tourniquet is removed, bleeding is pulsatile.
  **Table:**
  Triage zone?	Yellow
  Immediate action?	Replace tourniquet
  Tests?	CTA
  Treatment?	OR
  Dispo?	Admit
7. 19-year-old male with obvious deformity of left ankle. Talking, not ambulatory. Left dorsalis pedis pulse not present. Posterior tibial pulse present. Good cap refill.
  **Table:**
  Triage zone?	Yellow
  Tests?	XR
  Treatment:	Reduce No DP pulse after reduction
  Test?	CTA
  Consults?	Vascular/orthopedics
  Dispo?	OR, Admit
8. 4-year-old female with severe facial trauma. She is not breathing and has a heart rate of 40. Unable to obtain blood pressure.
  **Table:**
  Triage zone?	Red or black
  Next steps?	Attempt to open airway → does not start breathing
  Next steps?	Black zone/morgue
9. 56-year-old male with burns to his entire body. He is unable to ambulate. You can’t assess capillary refill due to burns. Soot in nares with circumferential extremity burns. Complaining of extreme pain and begging for pain medication. Asking if he is going to die.
  **Table:**
  Triage zone?	Red
  Event:	15 minutes later, he can’t feel his legs.
  Next steps?	Fasciotomy
  Tests?	XR for fractures
  Dispo?	Admit to burn
10. 44-year-old female missing the distal aspect of her right upper extremity. She has the mangled, dirty extremity in a bag. Appears to be detached distal to the elbow. She has no tourniquet in place and is bleeding everywhere. She appears unsteady. Capillary refill is five seconds.
  **Table:**
  Triage zone?	Red
  Next steps?	Apply tourniquet
  Imaging?	XR
  Consults?	Ortho, vascular
  Dispo?	OR, admit
11. 13-year-old male with a bleeding finger. He is ambulatory. He states his finger hurts. No obvious deformity.
  **Table:**
  Triage zone?	Green
  Tests?	XR → open tufts fracture
  Treatments?	Splint, +/− antibiotics, outpatient follow up
  Dispo?	Home
12. 8-year-old female is refusing to move. When you attempt to move her, she screams. She is holding her neck very still.
  **Table:**
  Triage zone:	Yellow
  Next Steps?	Put in c-collar
  Tests?	CT c-spine
  Treatment?	Spine consult, OR
  Disposition?	Admit
13. 10-year-old male confused and crying but directable and answering questions. Denies LOC, no signs of head trauma.
  **Table:**
  Triage zone:	Green
  Physical exam:	Normal neuro exam
  Next Steps?	Give parents return precautions, concussion precautions
  Tests?	None
  Dispo?	Home
14. 20-year-old male with metal rod sticking out of his lateral thigh. Pulses intact, no active bleeding. Patient reports 7/10 pain.
  **Table:**
  Triage zone?	Yellow
  Physical exam?	Pulses intact, sensation intact, motor intact but limited by pain
  Next Steps?	Stabilize
  Tests?	XR, consider CTA
  Treatment?	Remove rod, wash out, abx
  Dispo?	Home
15. 89-year-old female complaining of vision loss and pain to right eye. Reports feeling fluid from her eye when she rubbed it.
  **Table:**
  Triage zone?	Green
  Physical exam:	+ Seidel’s sign
  Next Steps?	Ophtho, abx, Zofran, shield
  Tests?	CTH, CT orbits
  Treatment?	OR with ophthalmology
16. 62-year-old male with skin avulsions to both arms. No signs of broken bones, full range of motion of bilateral upper extremities, sensation intact, no active bleeding.
  **Table:**
  Triage zone?	Green
  Next Steps?	Local wound care
  Tests?	None
  Dispo?	Home
17. 45-year-old male reports he got shocked working on the low voltage electrical lines when the accident happened. + LOC. Feels like his heart is racing. HR 156. He is not walking.
  **Table:**
  Triage zone?	Yellow
  Test?	EKG
  Tests?	CBC, CMP
  Treatment?	None, usually self-resolving
  Dispo?	Admit
18. 33-year-old female with large piece of building on her right leg. Pulses intact, sensation intact. Able to limp after block removed.
  **Table:**
  Triage zone?	Green
  Physical exam:	Contusion to her right leg, no other injury
  Tests?	Labs, CK x 2–3
  Treatment?	Fluids, pain control
  Dispo?	Observation vs discharge
19. 24-year-old male with large anterior forearm laceration with active pulsatile bleeding. He is attempting to cover his arm but the blood oozes around him holding pressure. Capillary refill > four seconds and he appears diaphoretic.
  **Table:**
  Triage zone?	Red
  Next Steps?	Tourniquet
  Tests?	CTA
  Treatment?	OR with vascular
20. Unknown age young male, severe burns and multiple bleeding wounds. Unable to palpate pulse. Has stridorous, agonal breathing.
  **Table:**
  Triage zone	Black
  Next steps?	Reposition airway
  Event:	No change in airway
  Dispo?	Black zone/morgue
21. 82-year-old female with large, bleeding head wound overlying the anterior forehead. She is able to ambulate but feels dizzy when she does so. She takes Coumadin.
  **Table:**
  Triage zone?	Green
  Tests?	CTH
  Treatment?	Laceration repair
  Dispo?	Discharge
22. 92-year-old male on Coumadin with large, bleeding posterior head wound. Wound measures 10cm. He seems a little confused but is answering questions.
  **Table:**
  Triage zone?	Yellow
  Tests?	CTH
  Treatment?	Call neurosurgery
  Dispo?	OR, admit
23. 19-year-old female with family, appears limp, has a slow pulse, not answering questions, squeezes your fingers when asked. Has labored, tachypneic breathing >30 breaths/minute.
  **Table:**
  Triage zone?	Red
  Next steps?	Jaw thrust
  Event:	No breathing/pulse
  Next steps?	Black zone
  Dispo?	Morgue
24. 33-year-old female with a deformity of her right thigh. She is not able to walk. She is screaming in pain.
  **Table:**
  Triage zone?	Yellow
  Tests?	XR femur
  Treatment?	Pain meds, reduce, splint, call ortho
  Dispo?	Dispo per ortho
25. 82-year-old male, tachypneic to the 40s, complaining of SOB.
  **Table:**
  Triage zone?	Red
  Physical exam:	Decreased breath sounds on right
  Immediate action?	Needle thoracostomy
  Definitive treatment:	Chest tube
  Dispo?	Admit
26. 55-year-old male with history of HTN, HLD, DM who presents with severe chest pain. His wife just died on scene. He is tachycardic to the 100s and tachypneic to the low 30s.
  **Table:**
  Triage zone?	Red
  Physical exam:	Diaphoretic, pale, ill appearing, minor abrasions
  Tests?	CBC, BMP, EKG, troponin.
  Treatment?	Aspirin, nitro, activate cath lab
  Dispo?	Admit
27. 44-year-old female, who was found with her left leg and hip crushed under debris, presents after extraction. She is tachycardic and hypotensive. She appears pale and diaphoretic. Capillary refill is five seconds.
  **Table:**
  Triage zone?	Red
  Physical exam?	Abrasions and minor lacerations to left hip and thigh. Pelvis unstable.
  Immediate next step?	Pelvic binder
  Tests?	CBC, CMP, CK, XR pelvis
  Treatment?	Start IVF, transfuse blood, OR with trauma, ortho, or IR
28. 13-year-old female with a foreign body lodged in her buttock. She has good capillary refill, is mentating well, but not able to walk.
  **Table:**
  Triage zone?	Yellow
  Physical exam?	Wound hemostatic but large hematoma
  Tests?	XR pelvis, CTA pelvis
  Treatment?	OR with vascular/IR
  Dispo?	Admit
29. 39-year-old male with multiple superficial foreign bodies in thigh. Not able to walk. Distal pulses intact.
  **Table:**
  Triage zone?	Yellow
  Physical exam?	Foreign bodies appear to be superficial pieces of broken material
  Tests?	XR femur
  Treatment?	Remove foreign bodies, wash, repair lacerations as needed
  Dispo?	Discharge
30. 42-year-old male brought in on a stretcher. EMS had not noticed he stopped breathing. You palpate an agonal pulse.
  **Table:**
  Triage zone?	Black
  Tests?	None
  Treatment?	None
  Dispo?	Morgue
31. 30-year-old female with shortness of breath. Patient is breathing at 35 breaths/minute but has good capillary refill and is awake and alert.
  **Table:**
  Triage zone?	Red
  Physical exam?	Decreased breath sounds on right.
  Tests?	None (clinical diagnosis)
  Treatment?	Needle decompression → chest tube
  Dispo?	Admit
32. 36-year-old female with shortness of breath. She is awake, alert, with good capillary refill but in obvious respiratory distress.
  **Table:**
  Triage zone?	Red
  Physical exam?	Open sucking chest wound over right lateral chest wall, frothy air noted with expirations.
  Tests?	None
  Treatment?	Occlusive/3-sided dressing
  Dispo?	Trauma surgery/admit
33. 22-year-old male with right knee pain. He is hobbling with a limp. He has intact distal pulses.
  **Table:**
  Triage zone?	Green
  Physical exam?	Right knee tender to touch, right patella displaced laterally.
  Tests?	None (post-reduction knee x-ray)
  Treatment?	Closed reduction, splint
  Dispo?	Discharge
34. 75-year-old female with bilateral hand numbness. Distal pulses intact with capillary refill less than two seconds. She is awake, alert.
  **Table:**
  Triage zone?	Green
  Physical exam?	Midline cervical spine tenderness. BL upper extremities with numbness, tingling, 4/5 strength. BL lower extremities 5/5 strength.
  Tests?	C-spine CT
  Treatment?	C-collar, spine consultation
  Dispo?	Admit
35. 45-year-old male with headache after fall from 10 feet. He is awake, alert, but disoriented and not following simple commands. His capillary refill is less than two seconds.
  **Table:**
  Triage zone?	Red
  Physical exam?	Small hematoma over right forehead,
  Event:	Repeats, “Am I going to be okay?”
  Tests?	CTH / C-spine
  Treatment?	Counsel on concussion
36. 33-year-old male with “abdominal pain” after metal fragment punctured abdomen. He has a capillary refill of three seconds, but is still alert and oriented.
  **Table:**
  Triage zone?	Red
  Physical exam?	Tachycardic to 120. Puncture wound with large metal fragment, no exit wound.
  Tests?	FAST (go to imaging for result)
  Treatment?	To OR
  Dispo?	Admit
37. 20-year-old female with ankle pain. Awake, alert, but unable to walk. No distal pulses appreciated. Capillary refill is three seconds on distal foot.
  **Table:**
  Triage zone?	Red
  Physical exam?	Obvious ankle deformity with no pulses in DP/PT
  Tests?	XR
  Treatment?	Closed reduction → pulses back, splint
  Dispo?	Discharge
38. 45-year-old female with “seizure” after smoke inhalation. Patient is post-ictal, breathing at 14 respirations/minute, and is only oriented to herself.
  **Table:**
  Triage zone?	Yellow
  Physical exam?	Skin is “cherry red” color, patient smells of bitter almonds.
  Tests?	CO, CBC, BMP, lactic acid, CK
  Treatment?	Hydroxocobalamin (or cyanokit: amyl nitrate and sodium thiosulfate) Oxygen, hyperbaric if possible
  Dispo?	Admit
39. 60-year-old male with altered mental status, was found down in a pool of water. He has agonal breathing.
  **Table:**
  Triage zone?	Red
  Physical exam?	Patient is rolled over and is GCS = 3, not following simple commands, slow respirations noted.
  Tests?	None
  Treatment?	Head tilt/chin lift → no improvement
  Dispo?	Black zone/morgue
40. 16-year-old female with “left leg pain,” screaming and grabbing at her leg, which is bleeding. She has distal capillary refill of five seconds.
  **Table:**
  Triage zone?	Red
  Physical exam?	Partial amputation noted to left tibia/fibula with active bleeding.
  Next steps?	Tourniquet
  Tests?	None, consider POC Hgb if admit delay
  Dispo?	OR/admit
41. 3-year-old male brought to you seizing. He has minor abrasions to his forehead and extremities. He is not breathing well. All of his extremities are shaking.
  **Table:**
  Triage zone?	Red
  Physical exam?	He is still shaking, now appears blue
  Next steps?	Versed IM
  Event:	Stops seizing
  Tests?	CBC, BMP, CTH
  Event:	Mental status improved, back at baseline
  Dispo?	Discharge
42. Patient is a 22-year-old male who was staring straight at the blast but was some distance away. He is complaining of eye pain. He is ambulatory and providing his own history, but his eyes are closed and he is tearful.
  **Table:**
  Triage zone?	Green
  Physical exam?	BL conjunctival injection, visual acuity 20/20 bilaterally when you get him to open his eyes
  Tests?	Fluorescein (must obtain image)
  Treatment?	Erythromycin ointment
  Dispo?	Discharge
43. Patient is a 9-month-old male, crying inconsolably, no obvious trauma.
  **Table:**
  Triage zone?	Yellow
  Physical exam?	No obvious trauma, no abrasions. Mom is holding him and also has no signs of trauma.
  Next steps?	Observation
  Event:	After two hours, patient is calm, happy, laughing and interacting appropriately.
  Dispo?	Discharge to home
44. Patient is a 92-year-old female who looks age 70. She has skin tears on her upper extremities. She states, “I’m fine dear. Take care of these other people.” She is mentating well, has good capillary refill, is ambulatory.
  **Table:**
  Triage zone?	Green
  Physical exam?	One 10 cm skin tear to RUE, two 5 cm skin tears to LUE
  Treatment?	Wash out, steri-strip
  Dispo?	Discharge
45. Patient is a 32-year-old female, G6P5, 37 weeks pregnant, who was at the game. She states, “my water broke.” No sign of trauma. She is ambulatory with no bleeding and good capillary refill.
  **Table:**
  Triage zone?	Green
  Physical exam?	Pants soaked with clear fluid
  Pelvic exam?	Patient’s cervix is 10 cm and you feel a baby’s head
  Treatment?	Call OB, prepare to deliver baby
  Event:	Congratulations! You delivered a healthy baby. OB delivered the placenta
  Dispo?	Admit to OB
46. Patient is a 22-year-old female, G1P0, 10 weeks pregnant, here with her husband. She states she has vaginal bleeding but otherwise looks okay.
  **Table:**
  Triage zone?	Green
  Event:	Patient waited one hour between triage and seeing you. She now has a capillary refill of three seconds, appears diaphoretic.
  Physical exam?	You can see bleeding through the patient’s pants. She is tachycardic and hypotensive
  Pelvic exam?	Large clots the size of a hand and significant bleeding of bright red blood obscures the cervix.
  Labs?	H&H, Type/Rh
  Treatment?	Call OB. Patient likely needs emergency D&C
  Dispo?	Admit to OB
47. Patient is a 32-year-old male with history of schizophrenia. He is shouting, “I am the devil and I am coming for you!!!!!!!!!!!!” He lunges at one of the triage nurses with a broken bit of plastic. Security is nowhere to be seen.
  **Table:**
  What do you do?	Attempt to de-escalate the situation while calling for help
  Next steps?	Security arrives and restrains the patient
  Treatment?	Sedative medications. Restraints. Isolation. Police if necessary
  Dispo?	Admit to psychiatry
48. A man wearing a large puffy coat approaches the triage desk. When he arrives, he opens his coat to reveal explosives.
  **Table:**
  What do you do?	Attempt to de-escalate the situation.
  Next steps?	Isolate patients as much as possible
  Event:	“I bombed the stadium, now I’m going to finish the job!”
  Next steps?	Police arrive and call bomb squad.
  Conclusion?	Patient is isolated by bomb squad. Disarmed.
  Dispo?	Jail.
49. Patient is a 32-year-old male screaming he wants to see his son. He is covered in blood. He tries to push through the barriers to get through security. You can’t tell where the blood is coming from.
  **Table:**
  Triage zone?	Green
  Next steps?	De-escalate the situation. Evaluate the patient
  Physical exam?	Patient has multiple abrasions on extremities. Has a large 10 cm piece of shrapnel in his right flank/lateral abdomen. Appears to have penetrated the peritoneum
  Consults?	Trauma surgery
  Dispo?	OR, admit
50. Patient is a 25-year-old female who presents with minor abrasions to her upper extremities.
  **Table:**
  Triage zone?	Green
  Event:	Patient is stung by a bee. Her face swells and she cannot breathe.
  Next steps?	Epinephrine, Benadryl, cetirizine/famotidine, steroids
  Event:	She still can’t breathe. She looks blue.
  Next steps?	Try to intubate
  Event:	Can’t intubate.
  Next steps?	Cricothyrotomy
  Event:	Patient ventilated appropriately. You saved her!
  Dispo?	Admit to MICU
51. Patient is a 27-year-old female who states she can’t hear. She is ambulatory. She has good capillary refill, no respiratory distress.
  **Table:**
  Triage zone?	Green
  Physical exam?	Otoscope examination reveals a perforated tympanic membrane.
  Treatment?	Clear foreign debris, otic drops, no swimming, keep dry
  Dispo?	Home
52. Patient is a 45-year-old male who is complaining of SOB. He is breathing at a rate of 45/minute.
  **Table:**
  Triage zone?	Red
  Tests?	CXR
  Treatment?	Supplemental O2, intubate if needed
  Dispo?	Admit
53. Patient is a 56-year-old male with history of COPD who presents with respiratory distress. He states the smoke is really bothering him. He is breathing 25 breaths/minute. He is ambulatory.
  **Table:**
  Triage?	Yellow
  Tests?	CXR
  Treatment?	DuoNeb, supplemental respiratory support as needed, steroids
  Event:	His breathing improves. He feels much better
  Dispo?	Home
54. Patient is a 20-something female with obvious evisceration injury to her abdomen. Her eyes are open. Her capillary refill is > four seconds. Her respirations are 8/min.
  **Table:**
  Triage zone?	Red
  Next Steps?	FAST, emergent OR
  Dispo?	Admit after OR
55. Patient is a 76-year-old male who was found face down in a pool of water. The right side of his body has mixed thickness burns. He is not breathing spontaneously. EMS tried to intubate him prior to arrival.
  **Table:**
  Triage zone?	Red
  Physical exam?	Listen for breath sounds → no breath sounds or chest rise
  Next steps?	Check tube position → not in the trachea
  Next steps?	Reposition tube
  Event:	Patient does not have pulses
  Next steps?	Black zone
  Tests?	None
  Dispo?	Morgue

### Appendix C: Instructions for Proctors

#### Sample Exercise Schedule

**Table:**

0900:	Interrupt conference
0905:	Everyone downstairs for exercise
0905–0925:	Divide into groups and assign tasks, determine triage system
0925–1015:	Triage and transport patients
0950:	All patients with a purple “*” on the corner of their card are found to be contaminated with radiation. This is communicated to the incident commander by the exercise lead. Learners must isolate and decontaminate these patients.
1015–1020:	Everyone back to classroom/lecture hall
1020–1045:	Wrap up
1045–1130:	Case review

#### Notes for proctors/Game rules

- *Divide into roles*: learners will be given a card with role and description of role. Learners must determine who will fill each role. The “incident commander” will volunteer or be appointed first. It is best if the incident commander role is filled by a senior resident. All other roles must be assigned by the “incident commander.” There will likely be more roles than residents. Residents may be reassigned by the “incident commander” after the start of the exercise, if needed.
- Required roles: “Incident commander,” initial “triage nurse/MD,” “surgeon” x3, “transporter” x3, “red zone MD,” “yellow zone MD,” “green zone MD,” “pharmacist,” and “hospitalist.” Additional roles assigned for extras as the need arises.
- The OR is considered full after each “surgeon” gets one case. An additional OR will be released as “surgeons” become available (on call “surgeons” reach the hospital - if there are medical students or extra people without roles, we can hold them until specified times - ie, one arrives at 0930, one at 0955, two at 1005).
- Each patient will be represented by a piece of paper which will have questions on it. This card will have initial patient prompt (ie, 33-year-old male with shrapnel injury). The initial question will ask, “triage zone?” The “triage doctor” (resident) will have to tell the proctor which zone (red/yellow/green/black) the patient should go to based on the prompt. The “triage doctor” can then scratch off the answer and send the patient to that zone. A “transporter” (resident or medical student) will have to take the patient to the appropriate zone.
- In each zone (red/yellow/green/black), the “resident MD/nurse” will then have additional prompts. The card may have “physical exam,” “next steps,” “tests,” “event,” “treatment,” “dispo,” or other prompt listed (see below for examples). If the prompt says “physical exam” or “event” the resident may scratch off the answer for that prompt *without* answering anything first. ALL OTHER PROMPTS, the resident must tell the proctor what they think the answer is before scratching off the answer. If the answer requires further action such as transport for labs/imaging, OR, or admission, the “transporter” must be called to transport the patient. If a learner must be reassigned to transport a patient, the “incident commander” must be called first. Each zone is responsible for seeing, treating, and disposition of all of their patients. There will be a proctor monitoring each zone (red/yellow/green zones, triage zone, labs and imaging zone, and admission zone/OR). That is six total required proctor roles.
- All medications must be obtained from the ED “pharmacist” (resident). The “pharmacist” will have a list of medications pre-printed. If other medications are needed, the “pharmacist” will have the ability to write-in those needed medications. Each zone will have to call the “pharmacist” when they need medications.

#### Proctor Roles

- We would like to have one proctor monitoring each of the zones (red/yellow/green/triage/labs and imaging/admission and OR). The red/yellow/green/triage zone proctors (total of three proctors) will have an answer key to ALL patients. Each patient will have a number which matches the patient to the proctor key. The residents in that zone must answer the questions on the patient prompts by telling the proctor their answer before they can scratch off the answer. The proctor can ask more questions or give further information as needed to guide residents to the answers.
- The labs/imaging proctor will be responsible for giving the imaging “transporter” (resident) the imaging information. The “transporter” will give the patient number to the proctor (who will have the number on the answer key) and tell the proctor which tests were ordered. The proctor will then have a packet of images, imaging reads, or labs for that patient number (Appendix D). The proctor can give the entire packet of images/labs to the “transporter” to take back to the patient’s zone if all of the tests were ordered. The imaging proctor may also ask the “transporter” questions about the tests for educational purposes, if desired.
- For example, case #1 has shrapnel in the leg (Appendix B). The patient is transported to the imaging proctor who will find the packet labeled case #1 (Appendix D) which will include an x-ray (XR), computed tomography (CT), CT angiogram (CTA), and hemoglobin. If the imaging tech or “transporter” requests the XR alone, only the XR is provided. If the “transporter” requests the CTA, XR, and hemoglobin, all are provided. If the zone requests further tests after the first trip to imaging/laboratory, the patient will have to be transported back to the lab/imaging proctor. If the proctor does not have a test that the “transporter” requests, the imaging proctor can make up a reason why it is not available (for example, the patient’s sample clotted, the XR is down, etc.). The patient card (seen by the residents) will not have the answers to which tests are available (Appendix B). Only the proctor answer key (Appendix C) will list which tests are available.
- The OR/admission proctor will be responsible for ensuring that the “surgeons” do not cheat on completing their Operation^®^ game before moving to the next patient. An example of cheating would be if the “surgeon” activates the buzzer on the Operation^®^ game and does not replace all the pieces and start over. The OR/admission proctor will also be responsible for suggesting appropriate triage of critical vs not critical OR cases, and ensuring proper admission sign-outs are given to the “admission physician(s)” or “hospitalist.”

#### Description of Game Play

- The “incident commander” will be a senior resident (third or fourth year). The “incident commander” will assign all roles. She is responsible for rearranging or reassigning roles as needed.
- *Required* resident roles: “Incident commander,” “triage lead,” “red zone lead,” “yellow zone lead,” “green zone lead,” at least one “transporter,” “pharmacist,” “hospitalist,” and at least one “surgeon.”
- Initial “triage doctor/nurse” will triage patients to zones.
- Each of the “doctors/nurses” in each zone (red/yellow/green) will determine what tests/treatments need to take place. The resident may scratch off the covering after answering the question with the proctor.
- “Transporters” must transport patients to imaging zones, OR, and admission. The imaging/laboratory proctor must provide the “transporter” with the image that corresponds to that patient (based on the patient number). The laboratory/imaging results and the patient are transported back to the zone from which they came. The designated zone team then decides if the patient must be admitted, go to the OR, or can be discharged. Transport must take the patient to the OR or to the hospital. Once the OR is full, nursing and MD staff must take charge of those patients until the OR is available (these roles could be filled by extra learners; however, ALL roles must be assigned by the “incident commander”).
- There will be three possible ORs (ie, three Operation^®^ games). The “surgeons” must remove all pieces from the operation without the buzzer going off before they can move to the next patient. If the buzzer goes off, the “surgeon” must replace the pieces and start over.
- The “incident commander” will have to determine what to do with deceased patients. There is a possible “coroner” role who can remove the patients from the public permanently; however, this role must be assigned. If there is no learner available to be “coroner,” the “incident commander” must devise a plan for placement of deceased patients.
- An “admissions physician” is required. This role will be fulfilled by any level resident. The “admissions physician” must take sign out on the patients and determine where to put them. There will be six available ED beds and 10 available beds in the hospital. Beyond this, the “admissions physician” must organize the patients and determine where to put them, who goes upstairs to the hospital floor, who goes to the ED, who stays in hallways, in chairs, outside, etc. If more physicians are assigned to be “admissions physicians,” more beds can become available. The number of beds per “admissions physician” or “surgeon” is listed on the “role card” which is distributed to the resident participants at the beginning of the exercise (Appendix E); also see Notes and Additional Materials. The “incident commander” retains the cards for unfilled roles and can redistribute them as needed during the exercise.

##### Notes and Additional Materials

The following are descriptions of roles that should be given to learners. In our implementation, the “incident commander” was given all of the roles and told to assign them appropriately. Each of the below descriptions were cut apart so the role and description could be given to the assigned learner. Learners were then given a stick-on name tag labeled with their assigned role so all other learners could easily identify the role each learner was playing. The “pharmacist” had additional materials (a medication list) which is attached below. The “pharmacist” was given the list of medications and then had to give the medication to the physicians in each zone to use. The “pharmacist” was the only person who had the power to request additional medications from the “incident commander” who would then tell the “pharmacist” whether the requested medications would be available or not. The available roles are below:

INCIDENT COMMANDER (required): assign all roles, reassign roles as needed, obtain resources.

TRIAGE LEAD (required): triage all patients. Determine whether patient goes to red, yellow, green, or black zones.

TRIAGE ASSIST: additional triage person to assist as needed (triage all patients; determine whether patient goes to red, yellow, green, or black zones).

GREEN LEAD/MD (required): complete physical exam, determine which tests need to be ordered, interpret tests, provide care, determine disposition, request resources from incident commander as needed.

YELLOW LEAD/MD (required): complete physical exam, determine which tests need to be ordered, interpret tests, provide care, determine disposition, request resources from incident commander as needed.

RED LEAD/MD (required): complete physical exam, determine which tests need to be ordered, interpret tests, provide care, determine disposition, request resources from incident commander as needed.

IMAGING TECH (optional): can assist imaging proctor as needed in giving test/imaging results to transporters to take back to zones.

TRANSPORT (required): transport patients from triage to appropriate zone, zone to testing areas, testing areas back to zone with test results, zone to OR, zone to admission, OR to admission.

TRANSPORT (optional): transport patients from triage to appropriate zone, zone to testing areas, testing areas back to zone with test results, zone to OR, zone to admission, OR to admission.

TRANSPORT (optional): transport patients from triage to appropriate zone, zone to testing areas, testing areas back to zone with test results, zone to OR, zone to admission, OR to admission.

SURGEON (required): must complete the full game of Operation^®^ for each patient WITHOUT the buzzer going off. If the buzzer goes off, the pieces must be replaced and start over. After OR case is over, surgeon must determine admit vs discharge. If admit, must sign out to admit doctor. If discharge, can discharge patient.

SURGEON (optional): must complete the full game of Operation^®^ for each patient WITHOUT the buzzer going off. If the buzzer goes off, the pieces must be replaced and start over. After OR case is over, surgeon must determine admit vs discharge. If admit, must sign out to admit doctor. If discharge, can discharge patient.

SURGEON (optional): must complete the full game of Operation^®^ for each patient WITHOUT the buzzer going off. If the buzzer goes off, the pieces must be replaced and start over. After OR case is over, surgeon must determine admit vs discharge. If admit, must sign out to admit doctor. If discharge, can discharge patient.

GREEN NURSE (optional): assist green lead with physical exam, tests, orders, medications, transport, and disposition of patients.

YELLOW NURSE (optional): assist yellow lead with physical exam, tests, orders, medications, transport, and disposition of patients.

RED NURSE (optional): assist red lead with physical exam, tests, orders, medications, transport, and disposition of patients.

OR TRANSPORT (optional): transport patients to the OR and from the OR. Can transport patients from OR to admit. Can assist OR with other tasks as designated by the surgeons.

MEDICINE ADMIT (required): must take sign out from surgeons and zone personnel. Must determine appropriate level of care for patients. Must determine which patients get beds first. Initially, you have six ED beds and 10 beds in the hospital. After that, you must determine what to do with these patients.

MEDICINE ADMIT (optional): must take sign out from surgeons and zone personnel. Must determine appropriate level of care for patients. Must determine which patients get beds first. Initially, you have six ED beds and 10 beds in the hospital. With the assignment of this optional hospitalist role, you get six more hospital beds. After that, you must determine what to do with these patients.

MICU ADMIT (optional): responsible for MICU admits (take sign out) and managing these patients. With this position filled, you have five additional MICU beds in addition to the six ED beds and 10 hospital beds.

SICU ADMIT (optional): responsible for SICU admits (take sign out) and managing these patients. With this position filled, you have five additional SICU beds in addition to the six ED beds and 10 hospital beds.

OR TECH (optional): assist surgeons in OR as needed. Surgeon to assign tasks.

OR TECH (optional): assist surgeons in OR as needed. Surgeon to assign tasks.

OR TECH (optional): assist surgeons in OR as needed. Surgeon to assign tasks.

PHARMACY (required): must provide all medications to triage patients in triage areas. If a medication you need is not listed, you may write in this medication. You may not administer medications. You must give medication to a nurse or doctor to administer.

CORONER (optional): responsible for removing bodies from the triage areas to safe, non-public locations.

OBSTETRICS AND GYNECOLOGY (OB/GYN) (optional): responsible for responding to OB/GYN emergencies. Will take sign out on patients and admit as needed. There are five available OB beds.

SECURITY (optional): responsible for responding to security issues and protecting personnel. Can restrain patients as needed.

POLICE DEPARTMENT (optional): responsible for responding to high level security threats and protecting triage personnel. Can arrest patients as needed.

ORTHOPEDICS (optional): can assist with reductions and OR. With specific orthopedic personnel, you have one extra OR in addition to the other three.

VASCULAR (optional): can assist with vascular repairs and consults. With specific vascular personnel, you have one extra OR in addition to the other three.

A suggested list of medications to give the “pharmacist” for use is as follows: Ativan, Benadryl, Haldol, Blood transfusion, Intravenous fluids, Morphine, Fentanyl, Dilaudid, Propofol, Versed, Etomidate, Succinylcholine, Rocuronium, Zofran, Reglan, Compazine, Platelets, Ancef, Unasyn, Ceftriaxone, Epinephrine, Levophed drip, Epinephrine drip, Dopamine drip, Dobutamine drip, Decadron, Cetirizine, and Famotidine.

#### Stations

There are several stations required for this activity. There should be one proctor per station. Depending on space, each station can be set up based on the “incident commander’s” preference or the lead proctor’s preference. We completed the event outside around a tall office building; however, one could also complete the activity inside in different offices, hallways, or a large event space. There should be three triage zones: red (immediate), yellow (delayed), and green (ambulatory), an initial triage area or entry, a space for admitted patients and surgical patients, and an imaging/laboratory station. The red, yellow, and green stations each had a tarp of the associated color to label their station. Learners would sit with the proctor at their assigned station. We chose to use hand-held radio communication to enhance the activity; therefore, we encouraged each station to set up in areas that were far from one another; however, this exercise could also be completed in close proximity without radios. The initial triage area was stationed on the opposite side of the building. This made patient transport and resource management more difficult and is a more realistic scenario. Our hospital/surgery area was located nearby but still out of talking distance from the red and yellow stations; therefore, radio communication was needed. The hospital admission/surgery areas had one proctor who oversaw the admissions and surgery teams and was stationed nearby, but also out of earshot of the other stations. The “surgeons” and “hospitalists” were based at the same station and thus able to communicate without use of a radio. The final station was the imaging/laboratory station. Learners were required to take their patients to the laboratory/imaging suite to obtain the images from the imaging/laboratory proctor. The proctor here used guiding questions to direct participants but there was no required set of questions for this proctor to ask.

The following is an example of how one patient may progress through the stations. Patient X arrives at initial triage and is seen by the “triage lead” learner and proctor. The initial prompt is given to the learner by the proctor. The “triage lead” determines whether patient X should be tagged red, yellow, green, or black. In this example, assume the patient was assigned to the yellow zone. Once the patient receives his designation, the patient is then transported by a learner “transporter” to the yellow zone. In the yellow zone, the zone proctor asks the “Yellow lead MD” and other MD/nursing staff questions on the sheet, guiding them as needed. After answering correctly, the proctor allows the learners to scratch off the answer. If it is determined imaging is needed, the patient must be transported by a “transporter” to the imaging/laboratory. The “transporter” requests the tests that the MD/nursing staff ordered. The imaging/laboratory proctor may ask questions as needed to guide learners if tests were not ordered that are available. The patient is then transported back to the yellow zone with the imaging/laboratory results. The remaining questions are answered and disposition is determined. If the disposition is surgery, the patient must be transported to the surgery area and the patient must be signed out to the “surgeon” either in person or on the radio. The “surgeon” can then discharge patients or admit them by signing them out to a hospitalist. If the patient does not need surgery but needs admission, then the yellow team must sign out to the “hospitalist” either in person or by radio. If the patient does not require surgery or admission, then the patient should be discharged and the zone should have record of these patients.

### Appendix D: Cases, Labs and Imaging for Proctors

#### Instructions for Use

1. Print this document
2. Provide one copy to each proctor to provide to learners as needed/requested

#### Case 1

**CTA**: Vascular structures normal. Foreign body embedded in anterior proximal thigh. No evidence of vascular injury.

**CBC w/o Differential**

White Blood Cell Count 8.5
RBC 5.12
Hgb 11.2
Hematocrit 36.4
MCV 88.1
MCH 28.7
MCHC 32.6
RDW-CV 14.0
PLT Count 182
MPV 8.2

#### Case 2

**Basic Metabolic Panel**

Sodium 136
Potassium 5.1
Chloride 102
CO2 26
Electrolyte Balance 8
Glucose 120
BUN 16
Creat 2.1
GFR Non-African Am… >60
GFR African Am… >60
Calcium 9.1

**Lactic Acid** = 4.1

**CBC w/o Differential**

White Blood Cell Count 12.5
RBC 5.12
Hgb 10.1
Hematocrit 34.2
MCV 88.1
MCH 28.7
MCHC 32.6
RDW-CV 14.0
PLT Count 145
MPV 8.2

#### Case 6

**CTA**: Bone structures normal. Lateral circumflex femoral artery extravasation. Recommend vascular surgery consultation.

#### Case 7

**CTA**: Bone structures intact. Edema surrounding medial and lateral malleoli. Extravasation from dorsalis pedis artery.

#### Case 9

**XR**: Multiple extremity x-rays of the bilateral humerus, bilateral forearm, bilateral wrist, bilateral hand, CXR, bilateral femur, bilateral tibia/fibula, bilateral ankle negative for acute fracture.

#### Case 12

Type II odontoid fracture

#### Case 14

**XR:** No fracture, foreign body in soft tissue

**CTA**: Vascular structures normal. Foreign body embedded in lateral thigh. No evidence of vascular injury. Soft tissue edema

#### Case 15

**CT**: Globe rupture of right eye. No other intracranial injury.

#### Case 18

**Basic Metabolic Panel**

Sodium 136
Potassium 4.1
Chloride 102
CO2 26
Electrolyte Balance 8
Glucose 120
BUN 16
Creat 0.7
GFR Non-African Am… >60
GFR African Am… >60
Calcium 9.1

**CBC w/Differential**

White Blood Cell Count 8.5
RBC 5.12
Hgb 14.7
Hematocrit 45.1
MCV 88.1
MCH 28.7
MCHC 32.6
RDW-CV 14.0
PLT Count 182
MPV 8.2
Neutrophils % (A) 65.9
ANC automated 5.6
Lymphocytes % 21.3
Lymphocytes Absolute 1.8
Monocytes % 9.5
Monocytes Absolute 0.8
Eosinophils % 2.9
Eosinophils Absolute 0.2
Basophils % 0.4
Basophils Absolute 0.0

#### Case 18

**Table:**

**CK**:	1500

#### Case 19

**CTA**: Radial artery injury, no bony injury

#### Case 26

**Basic Metabolic Panel**

Sodium 136
Potassium 4.1
Chloride 102
CO2 26
Electrolyte Balance 8
Glucose 120
BUN 16
Creat 0.7
GFR Non-African Am… >60
GFR African Am… >60
Calcium 9.1

**CBC w/Differential**

White Blood Cell Count 8.5
RBC 5.12
Hgb 14.7
Hematocrit 45.1
MCV 88.1
MCH 28.7
MCHC 32.6
RDW-CV 14.0
PLT Count 182
MPV 8.2
Neutrophils % (A) 65.9
ANC automated 5.6
Lymphocytes % 21.3
Lymphocytes Absolute 1.8
Monocytes % 9.5
Monocytes Absolute 0.8
Eosinophils % 2.9
Eosinophils Absolute 0.2
Basophils % 0.4
Basophils Absolute 0.0

#### Case 26

**Table:**

**EKG:**	ST elevations

**Table:**

**Troponin:**	0.11

#### Case 27

**Basic Metabolic Panel**

Sodium 136
Potassium 4.1
Chloride 102
CO2 26
Electrolyte Balance 8
Glucose 120
BUN 16
Creat 0.7
GFR Non-African Am… >60
GFR African Am… >60
Calcium 9.1

**CBC w/Differential**

White Blood Cell Count 8.5
RBC 5.12
Hgb 14.7
Hematocrit 45.1
MCV 88.1
MCH 28.7
MCHC 32.6
RDW-CV 14.0
PLT Count 182
MPV 8.2
Neutrophils % (A) 65.9
ANC automated 5.6
Lymphocytes % 21.3
Lymphocytes Absolute 1.8
Monocytes % 9.5
Monocytes Absolute 0.8
Eosinophils % 2.9
Eosinophils Absolute 0.2
Basophils % 0.4
Basophils Absolute 0.0

#### Case 27

**Table:**

**CK**:	3000

**Table:**

**XR**:	Open book pelvis

#### Case 28

**CTA:** No fracture. Arrow indicates arterial bleeding and pseudoaneurysm.

#### Case 34

C2 teardrop fracture

#### Case 35

**CT Head:**

**Table:**

**CT C-spine**:	Negative

#### Case 38

**Basic Metabolic Panel**

Sodium 136
Potassium 2.9
Chloride 102
CO2 26
Electrolyte Balance 8
Glucose 180
BUN 16
Creat 0.7
GFR Non-African Am… >60
GFR African Am… >60
Calcium 9.1

**CBC w/o Differential**

White Blood Cell Count 12.3
RBC 5.12
Hgb 14.7
Hematocrit 45.1
MCV 88.1
MCH 28.7
MCHC 32.6
RDW-CV 14.0
PLT Count 148
MPV 8.2
  **Table:**
  **Carboxyhemoglobin level:**	0.15 or 15%
  **Table:**
  **CK:**	1500
  **Table:**
  **Lactic acid:**	3.7

#### Case 41

**Basic Metabolic Panel**

Sodium 136
Potassium 4.1
Chloride 102
CO2 26
Electrolyte Balance 8
Glucose 120
BUN 16
Creat 0.7
GFR Non-African Am… >60
GFR African Am… >60
Calcium 9.1

**CBC w/Differential**

White Blood Cell Count 8.5
RBC 5.12
Hgb 14.7
Hematocrit 45.1
MCV 88.1
MCH 28.7
MCHC 32.6
RDW-CV 14.0
PLT Count 182
MPV 8.2
Neutrophils % (A) 65.9
ANC automated 5.6
Lymphocytes % 21.3
Lymphocytes Absolute 1.8
Monocytes % 9.5
Monocytes Absolute 0.8
Eosinophils % 2.9
Eosinophils Absolute 0.2
Basophils % 0.4
Basophils Absolute 0.0

#### Case 46

**CBC w/o Differential**

White Blood Cell Count 8.5
RBC 2.99
Hgb 8.1
Hematocrit 28.2
MCV 88.1
MCH 28.7
MCHC 32.6
RDW-CV 14.0
PLT Count 182
MPV 8.2
  **Table:**
  **Blood Type:**	AB+

### Appendix E: Roles and Descriptions Available Medications for Pharmacist

#### Instructions for Use

1. Print this document
2. Cut roles apart
3. Provide the “incident commander” (a senior resident, third or fourth year) the roles.
4. The “incident commander” will assign all roles. She is responsible for rearranging or reassigning roles as needed.

The following are descriptions of roles that should be given to learners. In our implementation, the “incident commander” was given all of the roles and told to assign them appropriately. Each of the below descriptions were cut apart so the role and description could be given to the assigned learner. Learners were then given a stick-on name tag labeled with their assigned role so all other learners could easily identify the role each learner was playing. The “pharmacist” had additional materials (a medication list) which is attached below. The “pharmacist” was given the list of medications and then had to give the medication to the physicians in each zone to use. The “pharmacist” was the only person who had the power to request additional medications from the “incident commander” who would then tell the “pharmacist” whether the requested medications would be available or not. The available roles are below:

**INCIDENT COMMANDER** (required): assign all roles, reassign roles as needed, obtain resources.

**TRIAGE LEAD** (required): triage all patients. Determine whether patient goes to red, yellow, green, or black zones.

**TRIAGE ASSIST**: additional triage person to assist as needed (triage all patients; determine whether patient goes to red, yellow, green, or black zones).

**GREEN LEAD/MD** (required): complete physical exam, determine which tests need to be ordered, interpret tests, provide care, determine disposition, request resources from incident commander as needed.

**YELLOW LEAD/MD** (required): complete physical exam, determine which tests need to be ordered, interpret tests, provide care, determine disposition, request resources from incident commander as needed.

**RED LEAD/MD** (required): complete physical exam, determine which tests need to be ordered, interpret tests, provide care, determine disposition, request resources from incident commander as needed.

**IMAGING TECH** (optional): can assist imaging proctor as needed in giving test/imaging results to transporters to take back to zones.

**TRANSPORT** (required): transport patients from triage to appropriate zone, zone to testing areas, testing areas back to zone with test results, zone to OR, zone to admission, OR to admission.

**TRANSPORT** (optional): transport patients from triage to appropriate zone, zone to testing areas, testing areas back to zone with test results, zone to OR, zone to admission, OR to admission.

**TRANSPORT** (optional): transport patients from triage to appropriate zone, zone to testing areas, testing areas back to zone with test results, zone to OR, zone to admission, OR to admission.

**SURGEON** (required): must complete the full game of Operation^®^ for each patient WITHOUT the buzzer going off. If the buzzer goes off, the pieces must be replaced and start over. After OR case is over, surgeon must determine admit vs discharge. If admit, must sign out to admit doctor. If discharge, can discharge patient.

**SURGEON** (optional): must complete the full game of Operation^®^ for each patient WITHOUT the buzzer going off. If the buzzer goes off, the pieces must be replaced and start over. After OR case is over, surgeon must determine admit vs discharge. If admit, must sign out to admit doctor. If discharge, can discharge patient.

**SURGEON** (optional): must complete the full game of Operation^®^ for each patient WITHOUT the buzzer going off. If the buzzer goes off, the pieces must be replaced and start over. After OR case is over, surgeon must determine admit vs discharge. If admit, must sign out to admit doctor. If discharge, can discharge patient.

**GREEN NURSE** (optional): assist green lead with physical exam, tests, orders, medications, transport, and disposition of patients.

**YELLOW NURSE** (optional): assist yellow lead with physical exam, tests, orders, medications, transport, and disposition of patients.

**RED NURSE** (optional): assist red lead with physical exam, tests, orders, medications, transport, and disposition of patients.

**OR TRANSPORT** (optional): transport patients to the OR and from the OR. Can transport patients from OR to admit. Can assist OR with other tasks as designated by the surgeons.

**MEDICINE ADMIT** (required): must take sign out from surgeons and zone personnel. Must determine appropriate level of care for patients. Must determine which patients get beds first. Initially, you have six ED beds and 10 beds in the hospital. After that, you must determine what to do with these patients.

**MEDICINE ADMIT** (optional): must take sign out from surgeons and zone personnel. Must determine appropriate level of care for patients. Must determine which patients get beds first. Initially, you have six ED beds and 10 beds in the hospital. With the assignment of this optional hospitalist role, you get six more hospital beds. After that, you must determine what to do with these patients.

**MICU ADMIT** (optional): responsible for MICU admits (take sign out) and managing these patients. With this position filled, you have five additional MICU beds in addition to the six ED beds and 10 hospital beds.

**SICU ADMIT** (optional): responsible for SICU admits (take sign out) and managing these patients. With this position filled, you have five additional SICU beds in addition to the six ED beds and 10 hospital beds.

**OR TECH** (optional): assist surgeons in OR as needed. Surgeon to assign tasks.

**OR TECH** (optional): assist surgeons in OR as needed. Surgeon to assign tasks.

**OR TECH** (optional): assist surgeons in OR as needed. Surgeon to assign tasks.

**PHARMACY** (required): must provide all medications to triage patients in triage areas. If a medication you need is not listed, you may write in this medication. You may not administer medications. You must give medication to a nurse or doctor to administer.

**CORONER** (optional): responsible for removing bodies from the triage areas to safe, non-public locations.

**OBSTETRICS AND GYNECOLOGY (OB/GYN)** (optional): responsible for responding to OB/GYN emergencies. Will take sign out on patients and admit as needed. There are five available OB beds.

**SECURITY** (optional): responsible for responding to security issues and protecting personnel. Can restrain patients as needed.

**POLICE DEPARTMENT** (optional): responsible for responding to high level security threats and protecting triage personnel. Can arrest patients as needed.

**ORTHOPEDICS** (optional): can assist with reductions and OR. With specific orthopedic personnel, you have one extra OR in addition to the other three.

**VASCULAR** (optional): can assist with vascular repairs and consults. With specific vascular personnel, you have one extra OR in addition to the other three.

**A suggested list of medications to give the “pharmacist” for use is as follows**:

Ativan
Benadryl
Haldol
Blood transfusion
Intravenous fluids
Morphine
Fentanyl
Dilaudid
Propofol
Versed
Etomidate
Succinylcholine
Rocuronium
Zofran
Reglan
Compazine
Platelets
Ancef
Unasyn
Ceftriaxone
Epinephrine
Levophed drip
Epinephrine drip
Dopamine drip
Dobutamine drip
Decadron
Cetirizine
Famotidine

### Appendix F: Small Group Application Exercise (sGAE) Answers Cases, Labs and Imaging Answers for Proctors

#### Instructions for Use

1. Print this document
2. Provide one copy to each proctor

1. 29-year-old female with foreign body in leg. She is able to ambulate with assistance from a friend. She has a tourniquet in place. It is still bleeding a lot, but her capillary refill is less than two seconds. She is breathing at 18/minute. The wound looks like it is pretty deep.
  **Table:**
  Triage zone?	Green
  Tests?	XR, CTA, Hgb
  Treatment?	Stabilize, OR

Her START triage criteria indicate she should go to the green zone because she has good capillary refill and is breathing at an appropriate rate.^11^ The prompt indicates that the patient has a lot of bleeding and a deep wound. An XR should be ordered to assess for foreign bodies and fractures. The CTA assesses vascular injury. Given the foreign body is embedded in the tissue and in close proximity to major vasculature, removal in the OR is most appropriate.^41^

#### Case 1

**CTA**: Vascular structures normal. Foreign body embedded in anterior proximal thigh. No evidence of vascular injury.

**CBC w/o Differential**

White Blood Cell Count 8.5
RBC 5.12
Hgb 11.2
Hematocrit 36.4
MCV 88.1
MCH 28.7
MCHC 32.6
RDW-CV 14.0
PLT Count 182
MPV 8.2

2. 30-year-old male with full body burns with large areas that appear white. Patient complaining of moderate, but not severe pain. He has soot in his oropharynx and nares and has a RR of 40.
  **Table:**
  Triage zone?	Red
  Tests?	CBC, CMP, lactate
  Treatment?	Wound care, IVF consider intubation
  Dispo?	Admit to burn

The START criteria indicate that this patient should be placed in the red zone because he is spontaneously breathing but has a RR >30.^11^ This patient likely has deep partial burns and requires immediate care. CBC and CMP are important in this critically injured patient to determine a baseline, especially for electrolytes. Lactate is useful to guide in management of shock. IVF should be administered based on Parkland formula. This patient, with soot in oropharynx and nares, likely has thermal injury to his airway and may require emergent intubation. He should be admitted to the burn team and may also require escharotomies for his extensive burns.^42^

#### Case 2

**Basic Metabolic Panel**

Sodium 136
Potassium 5.1
Chloride 102
CO2 26
Electrolyte Balance 8
Glucose 120
BUN 16
Creat 2.1
GFR Non-African Am... >60
GFR African Am... >60
Calcium 9.1

**Lactic Acid** = 4.1

**CBC w/o Differential**

White Blood Cell Count 12.5
RBC 5.12
Hgb 10.1
Hematocrit 34.2
MCV 88.1
MCH 28.7
MCHC 32.6
RDW-CV 14.0
PLT Count 145
MPV 8.2

3. 44-year-old male with sharp trauma to neck. Capillary refill of four seconds and the patient is not following simple commands. The patient is dripping blood everywhere. You cannot see if it is pulsatile under the bandages.
  **Table:**
  Triage zone?	Red
  Tests?	None - surgery to OR
  Treatment?	Apply pressure, surgery
  Dispo?	Admit

This patient is appropriate for the red zone, according to START criteria, because his capillary refill is > 4 seconds.^11^ He has “hard signs”, such as active bleeding and altered mental status, thus bleeding should be minimized with direct pressure and the patient should go directly to the OR.^43^

4. 21-year-old male, inebriated. He is shouting “my arm hurts!” There is an obvious deformity.
  **Table:**
  Triage zone?	Green
  Tests?	XR
  Treatment?	Reduce, splint
  Dispo?	Discharge, ortho follow up

This patient’s START criteria indicate that he should go to the green zone because he is able to walk.^11^ Given the obvious deformity, an XR is needed to assess for fracture/dislocation. As the prompt provides no evidence of neurovascular injury, his humeral fracture should be reduced and splinted, with ortho follow up.^44^

#### Case 4

5. 64-year-old female, breathing, not responding to commands, eyes closed, nonverbal. Appears to have a large parietal hematoma.
  **Table:**
  Triage zone?	Red
  Tests?	CTH
  Treatment?	Neurosurgery
  Dispo?	Admit to SICU

This patient, who is not responding to commands, should be placed in the red zone.^11^ A CTH is required to evaluate for intracranial hemorrhage. The CTH shows large epidural hematoma with midline shift. The patient requires evacuation of hemorrhage by neurosurgery.^45^

#### Case 5

6. 32-year-old male, not ambulatory, bleeding from proximal right thigh. He has a tourniquet in place. When the tourniquet is removed, bleeding is pulsatile.
  **Table:**
  Triage zone?	Yellow
  Immediate action?	Replace tourniquet
  Tests?	CTA
  Treatment?	OR
  Dispo?	Admit

This patient should be in the yellow zone because he is not ambulatory.^11^ He has an arterial injury given the pulsatile bleeding. A CTA should be ordered to better identify the vascular injury and associated injury. Because the CTA shows a vascular injury, the patient should be admitted to surgery for definitive repair.^46^

#### Case 6

**CTA**: Bone structures normal. Lateral circumflex femoral artery extravasation. Recommend vascular surgery consultation.

7. 19-year-old male with obvious deformity of left ankle. Talking, not ambulatory. Left dorsalis pedis pulse not present. Posterior tibial pulse present. Good cap refill.
  **Table:**
  Triage zone?	Yellow
  Tests?	XR
  Treatment:	Reduce No DP pulse after reduction
  Test?	CTA
  Consults?	Vascular/orthopedics
  Dispo?	OR, Admit

This patient is not ambulatory but has good capillary refill and intact mental status and should therefore be assigned to the yellow zone.^11^ XR is necessary to evaluate for fracture or other bony abnormality. He has no DP pulse, making reduction a critical step in his evaluation and treatment. Ankle dislocations should be reduced, with re-evaluation of distal pulses post-reduction. After reduction, this patient still does not have pulses distal to the injury, requiring CTA to assess for vascular injury. The presence of a vascular injury necessitates surgical management.^47^

#### Case 7

**CTA**: Bone structures intact. Edema surrounding medial and lateral malleoli. Extravasation from dorsalis pedis artery.

8. 4-year-old female with severe facial trauma. She is not breathing and has a heart rate of 40. Unable to obtain blood pressure.
  **Table:**
  Triage zone?	Red or black
  Next steps?	Attempt to open airway →does not start breathing
  Next steps?	Black zone/morgue

The first step in this patient who is not breathing is to position the airway to the optimal position to promote an open airway and facilitate breathing.^13^ After repositioning the airway, the patient did not start breathing so she should be assigned to the black zone.^11^

9. 56-year-old male with burns to his entire body. He is unable to ambulate. You can’t assess capillary refill due to burns. Soot in nares with circumferential extremity burns. Complaining of extreme pain and begging for pain medication. Asking if he is going to die.
  **Table:**
  Triage zone?	Red
  Event:	15 minutes later, he can’t feel his legs.
  Next steps?	Fasciotomy
  Tests?	XR for fractures
  Dispo?	Admit to burn

This patient meets criteria for the red zone because he is unable to walk and his capillary refill cannot be assessed.^11^ The development of paresthesias in his lower extremities suggests compartment syndrome, requiring fasciotomy to relieve compartment pressure.^42^ Additionally, extremity pain in the setting of trauma requires XR to rule out fractures as contributing to his pain and neurological deficits.

#### Case 9

**XR**: Multiple extremity x-rays of the bilateral humerus, bilateral forearm, bilateral wrist, bilateral hand, CXR, bilateral femur, bilateral tibia/fibula, bilateral ankle negative for acute fracture.

10. 44-year-old female missing the distal aspect of her right upper extremity. She has the mangled, dirty extremity in a bag. Appears to be detached distal to the elbow. She has no tourniquet in place and is bleeding everywhere. She appears unsteady. Capillary refill is five seconds.
  **Table:**
  Triage zone?	Red
  Next steps?	Apply tourniquet
  Imaging?	XR
  Consults?	Ortho, vascular
  Dispo?	OR, admit

With capillary refill greater than 2 seconds, this patient meets criteria for the red zone.^11^ The patient is actively hemorrhaging so the first step in management is to apply a tourniquet.^48^ The XR assesses the extent of bony damage, as well as for foreign body. This patient needs emergent surgery with orthopedics and vascular to control bleeding and attempt limb salvage.

#### Case 10

11. 13-year-old male with a bleeding finger. He is ambulatory. He states his finger hurts. No obvious deformity.
  **Table:**
  Triage zone?	Green
  Tests?	XR → open tufts fracture
  Treatments?	Splint, +/− antibiotics, outpatient follow up
  Dispo?	Home

This patient has a minor extremity injury and should be assigned to the green zone.^11^ An XR should be ordered to assess for fracture and foreign body. The finger should be splinted with the DIP in extension. There is no clear evidence recommending antibiotic prophylaxis in these wounds. If the physician considers the wound high risk for infection, then antibiotics can be given.^49^ The patient will require follow up with orthopedics on an outpatient basis.

#### Case 11

12. 8-year-old female is refusing to move. When you attempt to move her, she screams. She is holding her neck very still.
  **Table:**
  Triage zone:	Yellow
  Next Steps?	Put in c-collar
  Tests?	CT c-spine
  Treatment?	Spine consult, OR
  Disposition?	Admit

This patient is not ambulatory and should be placed in the yellow zone.^13^ NEXUS criteria can still be used for pediatric patients, though the original study did not enroll many patients less than 8 years old. This patient, with c-spine tenderness, does not meet criteria for c-spine clearance.^50^ CT should be ordered to evaluate for spinal injury. Type II odontoid fracture is an unstable fracture and should be managed operatively by the spine surgeons.

#### Case 12

Type II odontoid fracture

13. 10-year-old male confused and crying but directable and answering questions. Denies LOC, no signs of head trauma.
  **Table:**
  Triage zone:	Green
  Physical exam:	Normal neuro exam
  Next Steps?	Give parents return precautions, concussion precautions
  Tests?	None
  Dispo?	Home

This patient appears to be able to walk and should be placed in the green zone.^13^ On secondary triage, a neurological exam should be performed and, if normal, patient can be discharged home.

14. 20-year-old male with metal rod sticking out of his lateral thigh. Pulses intact, no active bleeding. Patient reports 7/10 pain.
  **Table:**
  Triage zone?	Yellow
  Physical exam?	Pulses intact, sensation intact, motor intact but limited by pain
  Next Steps?	Stabilize
  Tests?	XR, consider CTA
  Treatment?	Remove rod, wash out, abx
  Dispo?	Home

This patient has intact pulses and appears to be mentating well and should be placed in the yellow zone due to inability to ambulate.^11^ In a patient with penetrating extremity injury, it is important to assess for neurovascular injury. XR should be ordered to evaluate for fracture and presence of other foreign bodies. CTA assesses for vascular injury. If no vascular injury, rod can be removed, with appropriate wound care and prophylactic antibiotics.^51^

#### Case 14

**XR:** No fracture, foreign body in soft tissue

**CTA**: Vascular structures normal. Foreign body embedded in lateral thigh. No evidence of vascular injury. Soft tissue edema

15. 89-year-old female complaining of vision loss and pain to right eye. Reports feeling fluid from her eye when she rubbed it.
  **Table:**
  Triage zone?	Green
  Physical exam:	+ Seidel’s sign
  Next Steps?	Ophtho, abx, Zofran, shield
  Tests?	CTH, CT orbits
  Treatment?	OR with ophthalmology

This patient is able to walk and should be assigned to the green zone.^11^ However, her history is suggestive of possible globe rupture. She should be examined with a slit lamp and fluorescein. CT orbit should be ordered to assess for the presence of a foreign body and/or orbital fractures. CTH is to evaluate for other head injury in the setting of trauma. Management of ruptured globe includes emergent ophthalmology consult, systemic antibiotics, an anti-emetic to prevent increase intra-ocular pressure secondary to vomiting, and an eye shield to prevent further injury.^52^

#### Case 15

**CT**: Globe rupture of right eye. No other intracranial injury.

16. 62-year-old male with skin avulsions to both arms. No signs of broken bones, full range of motion of bilateral upper extremities, sensation intact, no active bleeding.
  **Table:**
  Triage zone?	Green
  Next Steps?	Local wound care
  Tests?	None
  Dispo?	Home

With the ability to ambulate and no evidence of serious injury, this patient should be placed in the green zone.^11^ His avulsions can be treated with appropriate local wound care, including washout and debridement if necessary, and he can be discharged home.

17. 45-year-old male reports he got shocked working on the low voltage electrical lines when the accident happened. + LOC. Feels like his heart is racing. HR 156. He is not walking.
  **Table:**
  Triage zone?	Yellow
  Test?	EKG
  Tests?	CBC, CMP
  Treatment?	None, usually self-resolving
  Dispo?	Admit

This patient should be placed in the yellow zone, due to his not walking, elevated heart rate, and potential for arrhythmia given significant tachycardia.^11^ EKG should be ordered to assess for arrythmia. Baseline labs, especially electrolytes, should be ordered in the setting of arrythmia and electrical injury to assess for tissue damage and electrolyte derangements. This patient’s atrial fibrillation, likely induced by electrical shock, will most likely self-resolve over time; however, rate control can be utilized.^24^

#### Case 17

18. 33-year-old female with large piece of building on her right leg. Pulses intact, sensation intact. Able to limp after block removed.
  **Table:**
  Triage zone?	Green
  Physical exam:	Contusion to her right leg, no other injury
  Tests?	Labs, CK x 2–3
  Treatment?	Fluids, pain control
  Dispo?	Observation vs discharge

This patient is able to ambulate and has intact pulses. She is therefore appropriate for the green zone.^11^ Physical exam should assess for any neurovascular injury or evidence of compartment syndrome. Metabolic panel should be ordered, especially to evaluate potassium level and renal function. CBC should also be ordered to assess patient’s hemoglobin level because there is possibility of bleeding and hematoma formation. CK is ordered to evaluate for rhabdomyolysis. This patient’s CK is elevated, suggesting possible rhabdomyolysis. The CK level may continue to rise for hours to days after the injury leading to complications such as renal failure. The first step in treating rhabdomyolysis is to administer IVF.^53^

#### Case 18

**Basic Metabolic Panel**

Sodium 136
Potassium 4.1
Chloride 102
CO2 26
Electrolyte Balance 8
Glucose 120
BUN 16
Creat 0.7
GFR Non-African Am... >60
GFR African Am... >60
Calcium 9.1

**CBC w/ Differential**

White Blood Cell Count 8.5
RBC 5.12
Hgb 14.7
Hematocrit 45.1
MCV 88.1
MCH 28.7
MCHC 32.6
RDW-CV 14.0
PLT Count 182
MPV 8.2
Neutrophils % (A) 65.9
ANC automated 5.6
Lymphocytes % 21.3
Lymphocytes Absolute 1.8
Monocytes % 9.5
Monocytes Absolute 0.8
Eosinophils % 2.9
Eosinophils Absolute 0.2
Basophils % 0.4
Basophils Absolute 0.0

#### Case 18

**Table:**

**CK**:	1500

19. 24-year-old male with large anterior forearm laceration with active pulsatile bleeding. He is attempting to cover his arm but the blood oozes around him holding pressure. Capillary refill > four seconds and he appears diaphoretic.
  **Table:**
  Triage zone?	Red
  Next Steps?	Tourniquet
  Tests?	CTA
  Treatment?	OR with vascular

This patient with capillary refill greater than four seconds and active bleeding meets criteria for the red zone.^11^ His extremity is actively bleeding and is not controlled by pressure. The next step is to apply a tourniquet.^48^ The CTA allows for characterization of the vascular injury. This patient should be admitted to the vascular surgery team for repair of his arterial injury.

#### Case 19

**CTA**: Radial artery injury, no bony injury

20. Unknown age young male, severe burns and multiple bleeding wounds. Unable to palpate pulse. Has stridorous, agonal breathing.
  **Table:**
  Triage zone?	Black
  Next steps?	Reposition airway
  Event:	No change in airway
  Dispo?	Black zone/morgue

This patient is currently breathing but his agonal respirations will likely progress to apnea. Additionally, he has no pulse. The next step is to reposition his airway to attempt to improve his breathing. If this does not improve his respiratory status, then he should be placed in the black zone.^11^

21. 82-year-old female with large, bleeding head wound overlying the anterior forehead. She is able to ambulate but feels dizzy when she does so. She takes Coumadin.
  **Table:**
  Triage zone?	Green
  Tests?	CTH
  Treatment?	Laceration repair
  Dispo?	Discharge

The patient is ambulatory and should be assigned to the green zone.^11^ CTH should be ordered to rule out an intracranial bleed, especially in the setting of anticoagulation.^54^ Since the CTH is negative, after her laceration is repaired, the patient can be discharged home.

#### Case 21

22. 92-year-old male on Coumadin with large, bleeding posterior head wound. Wound measures 10cm. He seems a little confused but is answering questions.
  **Table:**
  Triage zone?	Yellow
  Tests?	CTH
  Treatment?	Call neurosurgery
  Dispo?	OR, admit

Because patient is confused, he should be placed in the yellow zone.^11^ CTH is ordered to assess for intracranial hemorrhage. CTH shows a subarachnoid hemorrhage, so patient should be admitted to neurosurgery for further management.^55^

#### Case 22

23. 19-year-old female with family, appears limp, has a slow pulse, not answering questions, squeezes your fingers when asked. Has labored, tachypneic breathing >30 breaths/minute.
  **Table:**
  Triage zone?	Red
  Next steps?	Jaw thrust
  Event:	No breathing/pulse
  Next steps?	Black zone
  Dispo?	Morgue

This patient with her labored breathing and tachypnea should be placed in the red zone. The first step, according to the START algorithm, is to reposition the patient’s airway to improve breathing.^11^ After repositioning the airway, the patient is not breathing and has no pulse, so she should be placed in the black zone.^11^

24. 33-year-old female with a deformity of her right thigh. She is not able to walk. She is screaming in pain.
  **Table:**
  Triage zone?	Yellow
  Tests?	XR femur
  Treatment?	Pain meds, reduce, splint, call ortho
  Dispo?	Dispo per ortho

This patient is not able to walk but otherwise appears to have normal respirations and capillary refill, so she should be placed in the yellow zone.^11^ The XR should be ordered to assess for fracture or foreign body. The XR shows a femoral shaft fracture which should be reduced and splinted and then admitted to orthopedics.^56^

#### Case 24

25. 82-year-old male, tachypneic to the 40s, complaining of SOB.
  **Table:**
  Triage zone?	Red
  Physical exam:	Decreased breath sounds on right
  Immediate action?	Needle thoracostomy
  Definitive treatment:	Chest tube
  Dispo?	Admit

This patient has a respiratory rate >30 and therefore meets criteria for the red zone per START triage guidelines.^11^ The patient’s tachypnea, shortness of breath and decreased breath sounds suggest the diagnosis of pneumothorax. Treatment of tension pneumothorax is immediate needle thoracostomy followed by chest tube placement.^57^

26. 55-year-old male with history of HTN, HLD, DM who presents with severe chest pain. His wife just died on scene. He is tachycardic to the 100s and tachypneic to the low 30s.
  **Table:**
  Triage zone?	Red
  Physical exam:	Diaphoretic, pale, ill appearing, minor abrasions
  Tests?	CBC, BMP, EKG, troponin.
  Treatment?	Aspirin, nitro, activate cath lab
  Dispo?	Admit

This patient is tachypneic in the 30s, meeting criteria for the red zone.^11^ The patient’s presentation is concerning for acute coronary syndrome. CBC and BMP evaluate for hematologic abnormality or electrolyte level that may contribute to an arrhythmia. Troponin assesses for cardiac myocyte damage, while the EKG can assess for ischemia, infarction, arrhythmia, or other cardiac abnormalities. Workup reveals an elevated troponin and ST elevations in the lateral leads, for a diagnosis of STEMI. Aspirin and nitroglycerin should be initiated and the catheterization laboratory activated.^58^

#### Case 26

**Basic Metabolic Panel**

Sodium 136
Potassium 4.1
Chloride 102
CO2 26
Electrolyte Balance 8
Glucose 120
BUN 16
Creat 0.7
GFR Non-African Am... >60
GFR African Am... >60
Calcium 9.1

**CBC w/ Differential**

White Blood Cell Count 8.5
RBC 5.12
Hgb 14.7
Hematocrit 45.1
MCV 88.1
MCH 28.7
MCHC 32.6
RDW-CV 14.0
PLT Count 182
MPV 8.2
Neutrophils % (A) 65.9
ANC automated 5.6
Lymphocytes % 21.3
Lymphocytes Absolute 1.8
Monocytes % 9.5
Monocytes Absolute 0.8
Eosinophils % 2.9
Eosinophils Absolute 0.2
Basophils % 0.4
Basophils Absolute 0.0

#### Case 26

**Table:**

**EKG:**	ST elevations
**Troponin:**	0.11

#### Case 26

27. 44-year-old female, who was found with her left leg and hip crushed under debris, presents after extraction. She is tachycardic and hypotensive. She appears pale and diaphoretic. Capillary refill is five seconds.
  **Table:**
  Triage zone?	Red
  Physical exam?	Abrasions and minor lacerations to left hip and thigh. Pelvis unstable.
  Immediate next step?	Pelvic binder
  Tests?	CBC, CMP, CK, XR pelvis
  Treatment?	Start IVF, transfuse blood, OR with trauma, ortho, or IR

With a capillary refill of five seconds, this patient should be placed in the red zone.^11^ The first step in a patient with unstable pelvis, especially in the setting of shock, is to place a pelvic binder (ref below). The binder should be placed around the greater trochanters. CBC is ordered to assess for acute blood loss anemia. CMP is ordered to assess for end organ damage and electrolyte levels. CK should be ordered to assess for rhabdomyolysis. XR is to assess for pelvic fracture. The CK of 3000 suggests rhabdomyolysis and XR shows open book pelvis. This patient is in hemorrhagic shock in the setting of trauma. Management includes, IVF, blood transfusion, and definitive management in the operating room.^59^

#### Case 27

**Basic Metabolic Panel**

Sodium 136
Potassium 4.1
Chloride 102
CO2 26
Electrolyte Balance 8
Glucose 120
BUN 16
Creat 0.7
GFR Non-African Am... >60
GFR African Am... >60
Calcium 9.1

**CBC w/ Differential**

White Blood Cell Count 8.5
RBC 5.12
Hgb 14.7
Hematocrit 45.1
MCV 88.1
MCH 28.7
MCHC 32.6
RDW-CV 14.0
PLT Count 182
MPV 8.2
Neutrophils % (A) 65.9
ANC automated 5.6
Lymphocytes % 21.3
Lymphocytes Absolute 1.8
Monocytes % 9.5
Monocytes Absolute 0.8
Eosinophils % 2.9
Eosinophils Absolute 0.2
Basophils % 0.4
Basophils Absolute 0.0

#### Case 27

**Table:**

**CK**:	3000
**XR**:	Open book pelvis

#### Case 27

28. 13-year-old female with a foreign body lodged in her buttock. She has good capillary refill, is mentating well, but not able to walk.
  **Table:**
  Triage zone?	Yellow
  Physical exam?	Wound hemostatic but large hematoma
  Tests?	XR pelvis, CTA pelvis
  Treatment?	OR with vascular/IR
  Dispo?	Admit

This patient is unable to walk but otherwise has good capillary refill and mental status and should be placed in the yellow zone.^11^ XR should be ordered to assess for fracture and foreign body. CTA is needed to evaluate for vascular injury. Active arterial bleeding and pseudoaneurysm should be managed by vascular surgery or interventional radiology.^60^

#### Case 28

**CTA:** No fracture. Arrow indicates arterial bleeding and pseudoaneurysm.

29. 39-year-old male with multiple superficial foreign bodies in thigh. Not able to walk. Distal pulses intact.
  **Table:**
  Triage zone?	Yellow
  Physical exam?	Foreign bodies appear to be superficial pieces of broken material
  Tests?	XR femur
  Treatment?	Remove foreign bodies, wash, repair lacerations as needed
  Dispo?	Discharge

This patient is unable to walk but has intact distal pulses so he should be placed in the yellow zone.^11^ XR is to further evaluate the foreign bodies, as well as assess for any fracture. The XR shows that the foreign bodies are superficial and there is no evidence of fracture. Not all foreign bodies need to be removed, but those which are impairing patient function should be removed. Superficial foreign bodies can be removed by the emergency physician, followed by copious irrigation. 51

#### Case 29

30. 42-year-old male brought in on a stretcher. EMS had not noticed he stopped breathing. You palpate an agonal pulse.
  **Table:**
  Triage zone?	Black
  Tests?	None
  Treatment?	None
  Dispo?	Morgue

This patient does not have spontaneous respirations. The first step is to reposition the airway.^11^ If the patient is still not breathing, then he should be placed in the black zone, and no further care should be provided.^11^

31. 30-year-old female with shortness of breath. Patient is breathing at 35 breaths/minute but has good capillary refill and is awake and alert.
  **Table:**
  Triage zone?	Red
  Physical exam?	Decreased breath sounds on right.
  Tests?	None (clinical diagnosis)
  Treatment?	Needle decompression → chest tube
  Dispo?	Admit

This patient has a respiratory rate >30 breaths/minute and should therefore be placed in the red zone.^11^ Unilateral decreased breath sounds in a patient with dyspnea, especially in the setting of trauma, is most likely pneumothorax, and possibly tension pneumothorax. Pneumothorax is a clinical diagnosis and, if suspected, treatment is needle decompression in the second intercostal space, midclavicular line, followed by chest tube placement.^51^

32. 36-year-old female with shortness of breath. She is awake, alert, with good capillary refill but in obvious respiratory distress.
  **Table:**
  Triage zone?	Red
  Physical exam?	Open sucking chest wound over right lateral chest wall, frothy air noted with expirations.
  Tests?	None
  Treatment?	Occlusive/3-sided dressing
  Dispo?	Trauma surgery/admit

This patient is in respiratory distress and is likely tachypneic at a rate greater than 30 breaths/minute. Therefore, she should be placed in the red zone.^11^ Clinically, this patient appears to have an open pneumothorax. The management of open pneumothorax involves application of an occlusive dressing and admission for surgical repair.^61^

33. 22-year-old male with right knee pain. He is hobbling with a limp. He has intact distal pulses.
  **Table:**
  Triage zone?	Green
  Physical exam?	Right knee tender to touch, right patella displaced laterally.
  Tests?	None (post-reduction knee x-ray)
  Treatment?	Closed reduction, splint
  Dispo?	Discharge

This patient can ambulate and should be placed in the green zone.^11^ His physical exam is consistent with patellar dislocation. This is a clinical diagnosis that does not require imaging. After reduction, however, an x-ray should be performed to ensure proper location of the patella and rule out associated fracture.^62^ After reduction, a splint should be placed and the patient can be discharged home with orthopedic follow up.

34. 75-year-old female with bilateral hand numbness. Distal pulses intact with capillary refill less than two seconds. She is awake, alert.
  **Table:**
  Triage zone?	Green
  Physical exam?	Midline cervical spine tenderness. BL upper extremities with numbness, tingling, 4/5 strength. BL lower extremities 5/5 strength.
  Tests?	C-spine CT
  Treatment?	C-collar, spine consultation
  Dispo?	Admit

This patient has normal capillary refill and otherwise appears stable, so she meets criteria for the green zone.^11^ Her midline spinal tenderness and upper extremity neurologic deficits are suggestive of central cord syndrome. When there is concern for a cervical spine injury, it should be evaluated by CT.^50^ The CT shows a teardrop fracture, which is an unstable c-spine fracture. The patient should be placed in a c-collar and admitted for surgical management.

#### Case 34

C2 teardrop fracture

35. 45-year-old male with headache after fall from 10 feet. He is awake, alert, but disoriented and not following simple commands. His capillary refill is less than two seconds.
  **Table:**
  Triage zone?	Red
  Physical exam?	Small hematoma over right forehead,
  Event:	Repeats, “Am I going to be okay?”
  Tests?	CTH / C-spine
  Treatment?	Counsel on concussion

This patient is not following simple commands and should be placed in the red zone.^11^ Given the patient’s mechanism of injury and altered mental status, CT head and c-spine should be ordered to evaluate for intracranial and/or cervical spine injury.^63^ With negative imaging, the patient likely has a concussion, should be educated on management of his concussion at home, and discharged in the care of a competent individual.

#### Case 35

**CT Head:**

**Table:**

**CT C-spine**:	Negative

36. 33-year-old male with “abdominal pain” after metal fragment punctured abdomen. He has a capillary refill of three seconds, but is still alert and oriented.
  **Table:**
  Triage zone?	Red
  Physical exam?	Tachycardic to 120. Puncture wound with large metal fragment, no exit wound.
  Tests?	FAST (go to imaging for result)
  Treatment?	To OR
  Dispo?	Admit

This patient has capillary refill greater than two seconds and therefore meets criteria for the red zone.^11^ The FAST exam should be done to evaluate for bleeding in this patient with abdominal trauma and signs of shock. An unstable patient with a positive FAST exam should go directly to the OR for exploratory laparotomy.^64^

#### Case 36

37. 20-year-old female with ankle pain. Awake, alert, but unable to walk. No distal pulses appreciated. Capillary refill is three seconds on distal foot.
  **Table:**
  Triage zone?	Red
  Physical exam?	Obvious ankle deformity with no pulses in DP/PT
  Tests?	XR
  Treatment?	Closed reduction → pulses back, splint
  Dispo?	Discharge

With capillary refill that is greater than two seconds, as well as absent pulses with no collateral perfusion, this patient should be placed in the red zone.^11^ The XR evaluates for fracture. Ankle dislocations without associated fracture can be reduced and splinted.^47^ It is important to assess for presence of pulses after reduction to ensure adequate perfusion distal to the injury.

#### Case 37

38. 45-year-old female with “seizure” after smoke inhalation. Patient is post-ictal, breathing at 14 respirations/minute, and is only oriented to herself.
  **Table:**
  Triage zone?	Yellow
  Physical exam?	Skin is “cherry red” color, patient smells of bitter almonds.
  Tests?	CO, CBC, BMP, lactic acid, CK
  Treatment?	Hydroxocobalamin (or cyanokit: amyl nitrate and sodium thiosulfate) Oxygen, hyperbaric if possible
  Dispo?	Admit

This patient is post-ictal but able to say her name and thus appears that she can follow commands. Therefore, she should be placed in the yellow zone.^11^ Carbon monoxide (CO) and cyanide toxicity can co-occur in structural fires, especially in industrial sites. Both cyanide and CO toxicity can cause the “cherry red” skin color, though it is not a common finding. A bitter almond smell is a classic finding in cyanide toxicity. BMP should be ordered to assess for an anion gap metabolic acidosis and potassium levels. CBC is to assess for anemia. Lactate can be elevated in both CO and cyanide toxicity. CK assesses for muscular damage from hypoxia and anaerobic metabolism. To treat cyanide toxicity, hydroxocobalamin or cyanokit should be administered.^65^ CO toxicity is treated with 100% oxygen and hyperbaric oxygen if available given the patient had a seizure.^66^ A carboxyhemoglobin level of >25% would also be an indication for hyperbaric oxygen. This patient should be admitted for further monitoring.

#### Case 38

##### Basic Metabolic Panel

Sodium 136
Potassium 2.9
Chloride 102
CO2 26
Electrolyte Balance 8
Glucose 180
BUN 16
Creat 0.7
GFR Non-African Am... >60
GFR African Am... >60
Calcium 9.1

##### CBC w/o Differential

White Blood Cell Count 12.3
RBC 5.12
Hgb 14.7
Hematocrit 45.1
MCV 88.1
MCH 28.7
MCHC 32.6
RDW-CV 14.0
PLT Count 148
MPV 8.2

**Carboxyhemoglobin level:** 0.15 or 15%

**CK:** 1500

**Lactic acid:** 3.7

39. 60-year-old male with altered mental status, was found down in a pool of water. He has agonal breathing.
  **Table:**
  Triage zone?	Red
  Physical exam?	Patient is rolled over and is GCS = 3, not following simple commands, slow respirations noted.
  Tests?	None
  Treatment?	Head tilt/chin lift → no improvement
  Dispo?	Black zone/morgue

With agonal breathing, this patient likely has a respiratory rate greater than 30 and should be placed in the red zone.^11^ On further evaluation, his respiratory status is very tenuous. Repositioning of the head should be attempted to improve his breathing. If this maneuver does not improve his respirations, he should be placed in the black zone.^11^

40. 16-year-old female with “left leg pain,” screaming and grabbing at her leg, which is bleeding. She has distal capillary refill of five seconds.
  **Table:**
  Triage zone?	Red
  Physical exam?	Partial amputation noted to left tibia/fibula with active bleeding.
  Next steps?	Tourniquet
  Tests?	None, consider POC Hgb if admit delay
  Dispo?	OR/admit

This patient has a capillary refill of greater than five seconds so she should be placed in the red zone.^11^ Active bleeding should be stopped with a tourniquet and the patient should be taken to the OR emergently for definitive surgical repair.^48^

41. 3-year-old male brought to you seizing. He has minor abrasions to his forehead and extremities. He is not breathing well. All of his extremities are shaking.
  **Table:**
  Triage zone?	Red
  Physical exam?	He is still shaking, now appears blue
  Next steps?	Versed IM
  Event:	Stops seizing
  Tests?	CBC, BMP, CTH
  Event:	Mental status improved, back at baseline
  Dispo?	Discharge

This patient has a respiratory rate greater than 30 breaths/min and should be placed in the red zone.^13^ The patient appears to be having a seizure, which should be treated with benzodiazepines. After the seizure is aborted, workup should look for reversible causes. A BMP should be ordered to assess for hypoglycemia and electrolyte abnormalities such as hyponatremia. The CBC evaluates for anemia or signs of infection, such as leukocytosis. In the setting of trauma, a CT head is important to evaluate for intracranial bleed or other lesion. Once at baseline with normal workup, the patient can be discharged. There is no need for anti-epileptics for a first-time seizure.^67^

#### Case 41

##### Basic Metabolic Panel

Sodium 136
Potassium 4.1
Chloride 102
CO2 26
Electrolyte Balance 8
Glucose 120
BUN 16
Creat 0.7
GFR Non-African Am... >60
GFR African Am... >60
Calcium 9.1

##### CBC w/ Differential

White Blood Cell Count 8.5
RBC 5.12
Hgb 14.7
Hematocrit 45.1
MCV 88.1
MCH 28.7
MCHC 32.6
RDW-CV 14.0
PLT Count 182
MPV 8.2
Neutrophils % (A) 65.9
ANC automated 5.6
Lymphocytes % 21.3
Lymphocytes Absolute 1.8
Monocytes % 9.5
Monocytes Absolute 0.8
Eosinophils % 2.9
Eosinophils Absolute 0.2
Basophils % 0.4
Basophils Absolute 0.0

#### Case 41

42. Patient is a 22-year-old male who was staring straight at the blast but was some distance away. He is complaining of eye pain. He is ambulatory and providing his own history, but his eyes are closed and he is tearful.
  **Table:**
  Triage zone?	Green
  Physical exam?	BL conjunctival injection, visual acuity 20/20 bilaterally when you get him to open his eyes
  Tests?	Fluorescein (must obtain image)
  Treatment?	Erythromycin ointment
  Dispo?	Discharge

This patient is ambulatory and in stable condition. He should be placed in the green zone.^11^ A thorough exam should be performed, including a fluorescein exam to look for corneal abrasion, foreign body, or globe rupture. The fluorescein stain shows diffuse punctate lesions suggestive of ultraviolet photokeratitis. He should be treated with pain medications and antibiotic ointment.^68^

#### Case 42

43. Patient is a 9-month-old male, crying inconsolably, no obvious trauma.
  **Table:**
  Triage zone?	Yellow
  Physical exam?	No obvious trauma, no abrasions. Mom is holding him and also has no signs of trauma.
  Next steps?	Observation
  Event:	After two hours, patient is calm, happy, laughing and interacting appropriately.
  Dispo?	Discharge to home

This young patient appears uninjured, but his inconsolable crying is worrisome. Given his age less than one year, he should be placed in the yellow zone and re-evaluated (all patients under age one year are immediately categorized to the yellow zone).^13^ Upon re-evaluation, the patient is appropriate and has returned to his baseline. He can be discharged home with his mother.

44. Patient is a 92-year-old female who looks age 70. She has skin tears on her upper extremities. She states, “I’m fine dear. Take care of these other people.” She is mentating well, has good capillary refill, is ambulatory.
  **Table:**
  Triage zone?	Green
  Physical exam?	One 10 cm skin tear to RUE, two 5 cm skin tears to LUE
  Treatment?	Wash out, steri-strip
  Dispo?	Discharge

This patient is stable and can ambulate under her own strength. She should be placed in the green zone.^11^ Her simple skin tears should be cleaned and approximated with steri-strips.^69^

45. Patient is a 32-year-old female, G6P5, 37 weeks pregnant, who was at the game. She states, “my water broke.” No sign of trauma. She is ambulatory with no bleeding and good capillary refill.
  **Table:**
  Triage zone?	Green
  Physical exam?	Pants soaked with clear fluid
  Pelvic exam?	Patient’s cervix is 10 cm and you feel a baby’s head
  Treatment?	Call OB, prepare to deliver baby
  Event:	Congratulations! You delivered a healthy baby. OB delivered the placenta
  Dispo?	Admit to OB

This patient, who can ambulate and has good capillary refill, should be placed in the green zone.^11^ On secondary triage, it is clear that this woman is in labor. It is important to alert OB immediately in case it is a complicated delivery.^51^ After delivery, the patient should be admitted to the OB team for further care both of the patient and the new baby.

46. Patient is a 22-year-old female, G1P0, 10 weeks pregnant, here with her husband. She states she has vaginal bleeding but otherwise looks okay.
  **Table:**
  Triage zone?	Green
  Event:	Patient waited one hour between triage and seeing you. She now has a capillary refill of three seconds, appears diaphoretic.
  Physical exam?	You can see bleeding through the patient’s pants. She is tachycardic and hypotensive
  Pelvic exam?	Large clots the size of a hand and significant bleeding of bright red blood obscures the cervix.
  Labs?	H&H, Type/Rh
  Treatment?	Call OB. Patient likely needs emergency D&C
  Dispo?	Admit to OB

This patient is ambulatory and appears to have a normal respiratory rate and capillary refill. She should be placed in the green zone.^11^ On re-examination, the patient has clinically deteriorated, and should receive immediate intervention. The physical exam should include a pelvic exam. Her deterioration is consistent with hemorrhagic shock, likely from an incomplete abortion. H&H assists with quantifying the patient’s blood loss. Type and Rh should be ordered in case the patient requires blood transfusion and to determine if RhoGam is needed. The patient should be resuscitated with IVF and blood. The patient is Rh+ and does not require RhoGam. OB should be called because this patient likely needs definitive management with dilation and curettage.^70^

#### Case 46

##### CBC w/o Differential

White Blood Cell Count 8.5
RBC 2.99
Hgb 8.1
Hematocrit 28.2
MCV 88.1
MCH 28.7
MCHC 32.6
RDW-CV 14.0
PLT Count 182
MPV 8.2

**Blood Type:** AB+

47. Patient is a 32-year-old male with history of schizophrenia. He is shouting, “I am the devil and I am coming for you!!!!!!!!!!!!” He lunges at one of the triage nurses with a broken bit of plastic. Security is nowhere to be seen.
  **Table:**
  What do you do?	Attempt to de-escalate the situation while calling for help
  Next steps?	Security arrives and restrains the patient
  Treatment?	Sedative medications. Restraints. Isolation. Police if necessary
  Dispo?	Admit to psychiatry

This scenario is not specifically in the START algorithm but the patient is ambulatory and presumably could be categorized as green.^11^ In this situation it is crucial to call for help immediately, while attempting to calm the patient without injuring yourself or anyone else in the area. Options for chemical sedation include benzodiazepines, antipsychotics, or a combination. There is some evidence that a combination may be safer and more effective than either class on its own.^71^ Once calmed and restrained, the patient should be admitted for inpatient psychiatric therapy.

48. A man wearing a large puffy coat approaches the triage desk. When he arrives, he opens his coat to reveal explosives.
  **Table:**
  What do you do?	Attempt to de-escalate the situation.
  Next steps?	Isolate patients as much as possible
  Event:	“I bombed the stadium, now I’m going to finish the job!”
  Next steps?	Police arrive and call bomb squad.
  Conclusion?	Patient is isolated by bomb squad. Disarmed.
  Dispo?	Jail.

This man is uninjured and poses a safety threat to everyone in the vicinity. The first step is to attempt to provide safety for yourself, the patients, and the staff. Security and the police should be called, and the man should be isolated as much as possible and de-escalation could be attempted by the appropriate authorities. Once the police arrive, the management of the situation is per their protocol.

49. Patient is a 32-year-old male screaming he wants to see his son. He is covered in blood. He tries to push through the barriers to get through security. You can’t tell where the blood is coming from.
  **Table:**
  Triage zone?	Green
  Next steps?	De-escalate the situation. Evaluate the patient
  Physical exam?	Patient has multiple abrasions on extremities. Has a large 10 cm piece of shrapnel in his right flank/lateral abdomen. Appears to have penetrated the peritoneum
  Consults?	Trauma surgery
  Dispo?	OR, admit

This patient is ambulatory and should be placed in the green zone.^11^ Additionally, since he is agitated, attempts should be made to calm him verbally, if possible.^73^ On secondary triage, his injuries are noted to be more severe than previously noted. Because there is concern for intra-abdominal injury, the trauma service should be consulted. If the patient is hemodynamically unstable and/or has evidence of peritonitis, the patient should be taken directly to the OR. If stable without signs of peritonitis, CT imaging is ordered to assess for hollow viscous injury.^73^

50. Patient is a 25-year-old female who presents with minor abrasions to her upper extremities.
  **Table:**
  Triage zone?	Green
  Event:	Patient is stung by a bee. Her face swells and she cannot breathe.
  Next steps?	Epinephrine, Benadryl, cetirizine/famotidine, steroids
  Event:	She still can’t breathe. She looks blue.
  Next steps?	Try to intubate
  Event:	Can’t intubate.
  Next steps?	Cricothyrotomy
  Event:	Patient ventilated appropriately. You saved her!
  Dispo?	Admit to MICU

Initially, this patient has minor injuries and should be placed in the green zone.^11^ After being stung, she is experiencing anaphylaxis and should be treated with epinephrine, first- and second-generation antihistamines, and steroids. If pharmacologic treatment is not effective, her airway must be secured and she should be intubated.^74^ In this case, she cannot be intubated due to her airway edema; therefore; an emergency cricothyrotomy is indicated to maintain her airway. An epinephrine drip should be started. After the patient is stabilized, she should be admitted to the ICU for further management.

51. Patient is a 27-year-old female who states she can’t hear. She is ambulatory. She has good capillary refill, no respiratory distress.
  **Table:**
  Triage zone?	Green
  Physical exam?	Otoscope examination reveals a perforated tympanic membrane.
  Treatment?	Clear foreign debris, otic drops, no swimming, keep dry
  Dispo?	Home

This patient is ambulatory with good capillary refill, meeting criteria for the green zone.^11^ This patient has a tympanic membrane rupture, classified as a primary blast injury. Most tympanic membrane perforations heal spontaneously. It is important to keep the middle ear dry. Topical antibiotic drops (eg, ciprofloxacin) should be administered.^51^

52. Patient is a 45-year-old male who is complaining of SOB. He is breathing at a rate of 45/minute.
  **Table:**
  Triage zone?	Red
  Tests?	CXR
  Treatment?	Supplemental O2, intubate if needed
  Dispo?	Admit

With a respiratory rate greater than 30, this patient should be placed in the red zone.^11^ CXR examines for potential causes of SOB including pneumothorax, pleural effusion, fracture, etc. This CXR shows a pulmonary contusion (“blast lung”), an example of a primary blast injury.^75^ Management is supportive with pain control and ventilatory assistance, if needed. There should be a low threshold to intubate these patients.

#### Case 52

53. Patient is a 56-year-old male with history of COPD who presents with respiratory distress. He states the smoke is really bothering him. He is breathing 25 breaths/minute. He is ambulatory.
  **Table:**
  Triage?	Yellow
  Tests?	CXR
  Treatment?	DuoNeb, supplemental respiratory support as needed, steroids
  Event:	His breathing improves. He feels much better
  Dispo?	Home

This patient is ambulatory, but given his respiratory distress and medical history, he should be placed in the yellow zone because he is at risk of decompensating rapidly.^11^ CXR is to evaluate for causes of SOB such as pneumothorax, pleural effusion, rib fracture, or pneumonia. His CXR shows hyperinflation, as expected in a COPD patient. He is experiencing a COPD exacerbation and should be treated with bronchodilators, steroids and respiratory support as needed.^76^ After treatment, he improves and can be discharged on his home medications.

#### Case 53

54. Patient is a 20-something female with obvious evisceration injury to her abdomen. Her eyes are open. Her capillary refill is > four seconds. Her respirations are 8/min.
  **Table:**
  Triage zone?	Red
  Next Steps?	FAST, emergent OR
  Dispo?	Admit after OR

This patient has a capillary refill greater than three seconds, meeting criteria for the red zone.^11^ This patient has an evisceration injury and should go to the OR for an emergent exploratory laparotomy.^77^ FAST can be performed to further characterize the patient’s injury, but should not delay patient’s transport to the OR.

#### Case 54

55. Patient is a 76-year-old male who was found face down in a pool of water. The right side of his body has mixed thickness burns. He is not breathing spontaneously. EMS tried to intubate him prior to arrival.
  **Table:**
  Triage zone?	Red
  Physical exam?	Listen for breath sounds → no breath sounds or chest rise
  Next steps?	Check tube position → not in the trachea
  Next steps?	Reposition tube
  Event:	Patient does not have pulses
  Next steps?	Black zone
  Tests?	None
  Dispo?	Morgue

This patient was intubated prior to arrival, but is not breathing spontaneously. He should initially be placed in the red zone for immediate evaluation.^11^ The first step should be to ensure proper placement of the tube. After repositioning the tube, if the patient’s condition improves, he should remain in the red zone. However, in this case, the patient continues to be apneic and does not have pulses, so he should be placed in the black zone.^11^

#### Brief Wrap Up (Optional)

We completed a debrief with the residents and proctors and asked the participants to think about their various roles, what went well, and what was difficult to manage. We asked them to consider how these problems could be solved in a real MCI scenario. We discussed how we would prepare for events such as this in the future and changes we would implement based on our mock scenario. We also asked for feedback on what went well and what needed improvement specifically for the future use of the activity. We received very useful suggestions which we have incorporated into this publication. Example slides for the wrap up are included in the PowerPoint slide deck in Appendix A.

#### Pre and Post Exercise Assessment

**Name: _____________________________**

1. Name the basic START patient categories:
  - ________________________________
  - ________________________________
  - ________________________________
  - ________________________________
2. List the physical exam/vital signs associated with each category:
  - ________________________________
  - ________________________________
  - ________________________________
  - ________________________________
3. What role does the incident commander fulfill in a mass casualty incident? Name at least one responsibility of the incident commander.
  __________________________________________________________________________
  __________________________________________________________________________
  __________________________________________________________________________

**In which triage zone should each of the following patients be categorized?**

4. 82-year-old male, tachypneic to the 40s, complaining of SOB.
  ________________________________________
5. 13-year-old female with a foreign body lodged in her buttock. She has good capillary refill, is mentating well, but not able to walk.
  ________________________________________
6. 42-year-old male brought in on a stretcher. EMS had not noticed he stopped breathing. You palpate an agonal pulse.
  ________________________________________
7. 22-year-old male with right knee pain. He is hobbling with a limp. He has intact distal pulses.
  ________________________________________
8. 45-year-old male with headache after fall from 10 feet. He is awake, alert, but disoriented and not following simple commands. His capillary refill is less than two seconds.
  ________________________________________
9. Patient is a 22-year-old male who was staring straight at the blast but was some distance away. He is complaining of eye pain. He is ambulatory and providing his own history, but his eyes are closed and he is tearful.
  ________________________________________
10. Patient is a 9-month-old male, crying inconsolably, no obvious trauma.
  ________________________________________

#### Pre and Post Exercise Assessment Key

1. Name the basic START patient categories:
  - Green: ambulatory, minor injuries
  - Yellow: delayed, serious
  - Red: immediate, critical
  - Black: deceased, expectant
2. List the physical exam/vital signs associated with each category:
  - Green: ambulatory, follows commands
  - Yellow: respirations <30/min, cap refill <2 seconds, follows commands
  - Red: respirations <10/min or >30/min, cap refill >2 sec, does not follow commands, begins breathing after airway repositioning
  - Black: pulseless/not breathing/agonal breathing
3. What role does the incident commander fulfill in a mass casualty incident? Name at least one responsibility of the incident commander.
  Assign roles
  Give instructions to all staff
  Monitor situation
  Obtain/provide resources
  Reallocate resources as needed
  Patient and staff safety

**In which triage zone should each of the following patients be categorized?**

4. 82-year-old male, tachypneic to the 40s, complaining of SOB.
  red
5. 13-year-old female with a foreign body lodged in her buttock. She has good capillary refill, is mentating well, but not able to walk.
  yellow
6. 42-year-old male brought in on a stretcher. EMS had not noticed he stopped breathing. You palpate an agonal pulse.
  black
7. 22-year-old male with right knee pain. He is hobbling with a limp. He has intact distal pulses.
  green
8. 45-year-old male with headache after fall from 10 feet. He is awake, alert, but disoriented and not following simple commands. His capillary refill is less than two seconds.
  red
9. Patient is a 22-year-old male who was staring straight at the blast but was some distance away. He is complaining of eye pain. He is ambulatory and providing his own history, but his eyes are closed and he is tearful.
  green
10. Patient is a 9-month-old male, crying inconsolably, no obvious trauma.
  yellow
