# Supplementary material for: Simulated Mass Casualty Incident Triage Exercise for Training Medical Personnel
Source: J Educ Teach Emerg Med. 2020 Oct 15;5(4):SG1–SG231. doi: 10.21980/J82H1R (PMC10334446; doi:10.21980/J82H1R)
Supplement: Supplementary file 1 [file jetem-5-4-sg1-supp1.pptx]

## Slide 1
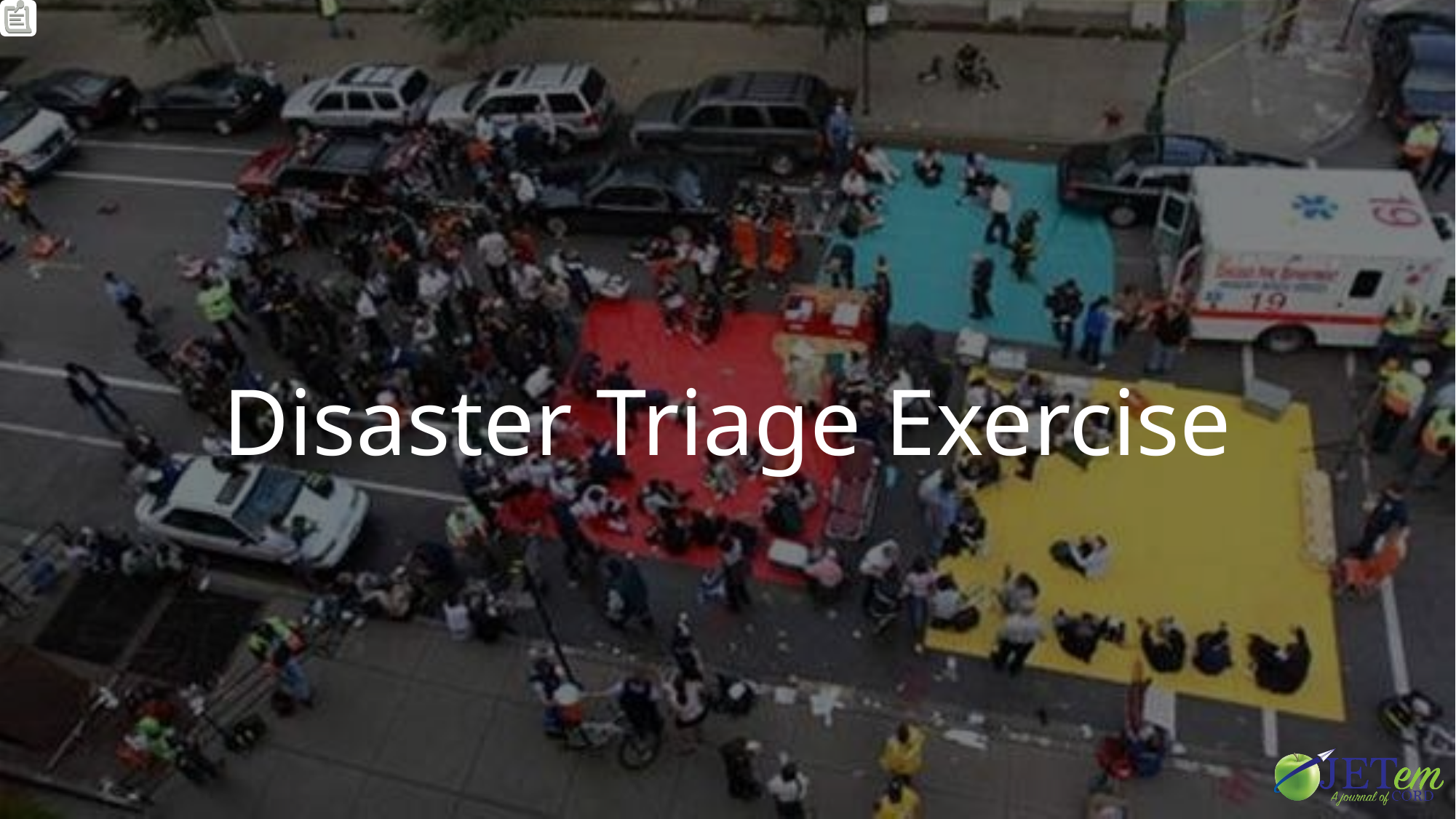

# Disaster Triage Exercise

## Slide 2
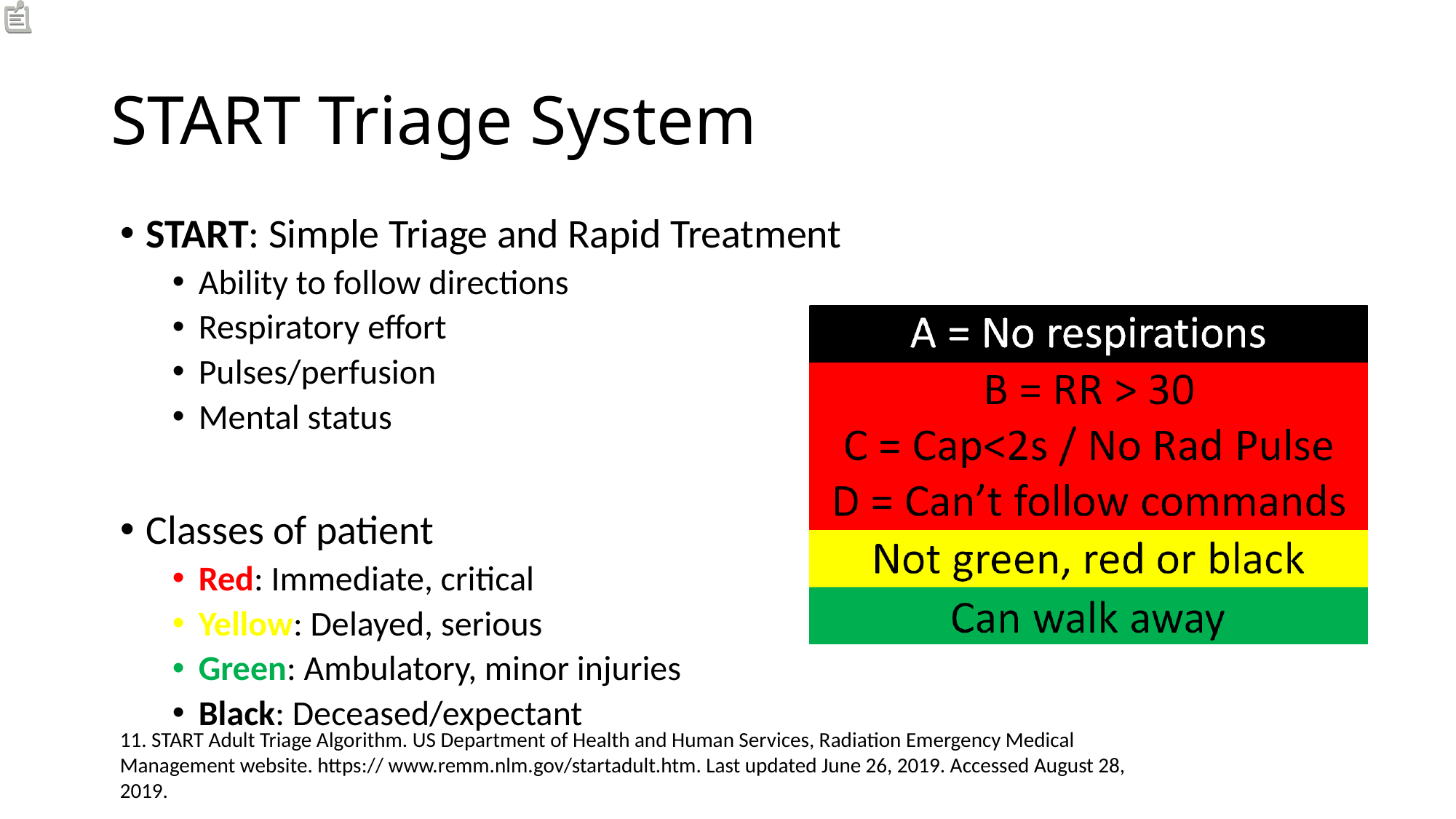

# START Triage System
START: Simple Triage and Rapid Treatment
Ability to follow directions
Respiratory effort
Pulses/perfusion
Mental status
Classes of patient
Red: Immediate, critical
Yellow: Delayed, serious
Green: Ambulatory, minor injuries
Black: Deceased/expectant
11. START Adult Triage Algorithm. US Department of Health and Human Services, Radiation Emergency Medical Management website. https:// www.remm.nlm.gov/startadult.htm. Last updated June 26, 2019. Accessed August 28, 2019.

## Slide 3
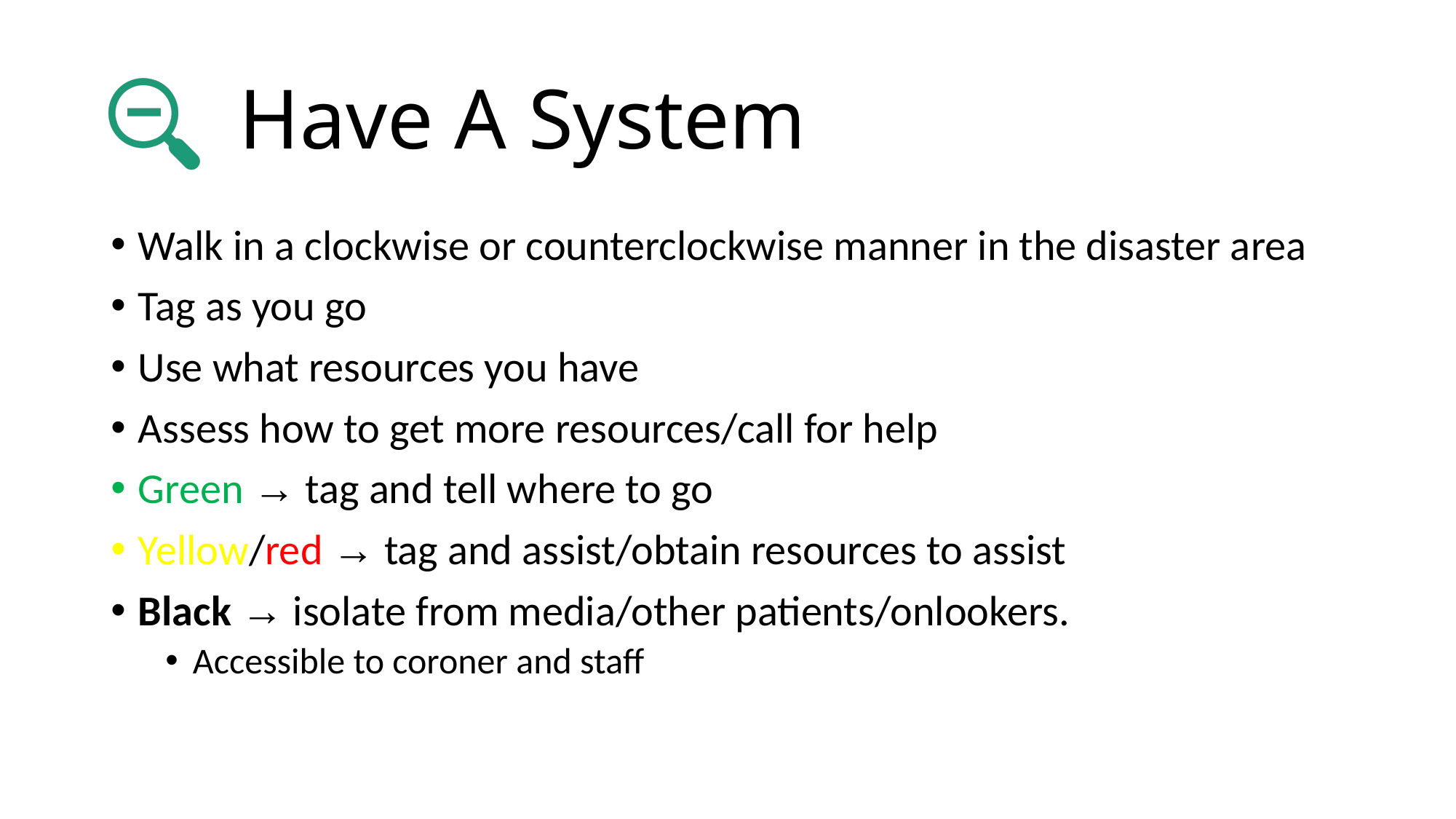

# Have A System
Walk in a clockwise or counterclockwise manner in the disaster area
Tag as you go
Use what resources you have
Assess how to get more resources/call for help
Green → tag and tell where to go
Yellow/red → tag and assist/obtain resources to assist
Black → isolate from media/other patients/onlookers.
Accessible to coroner and staff

## Slide 4
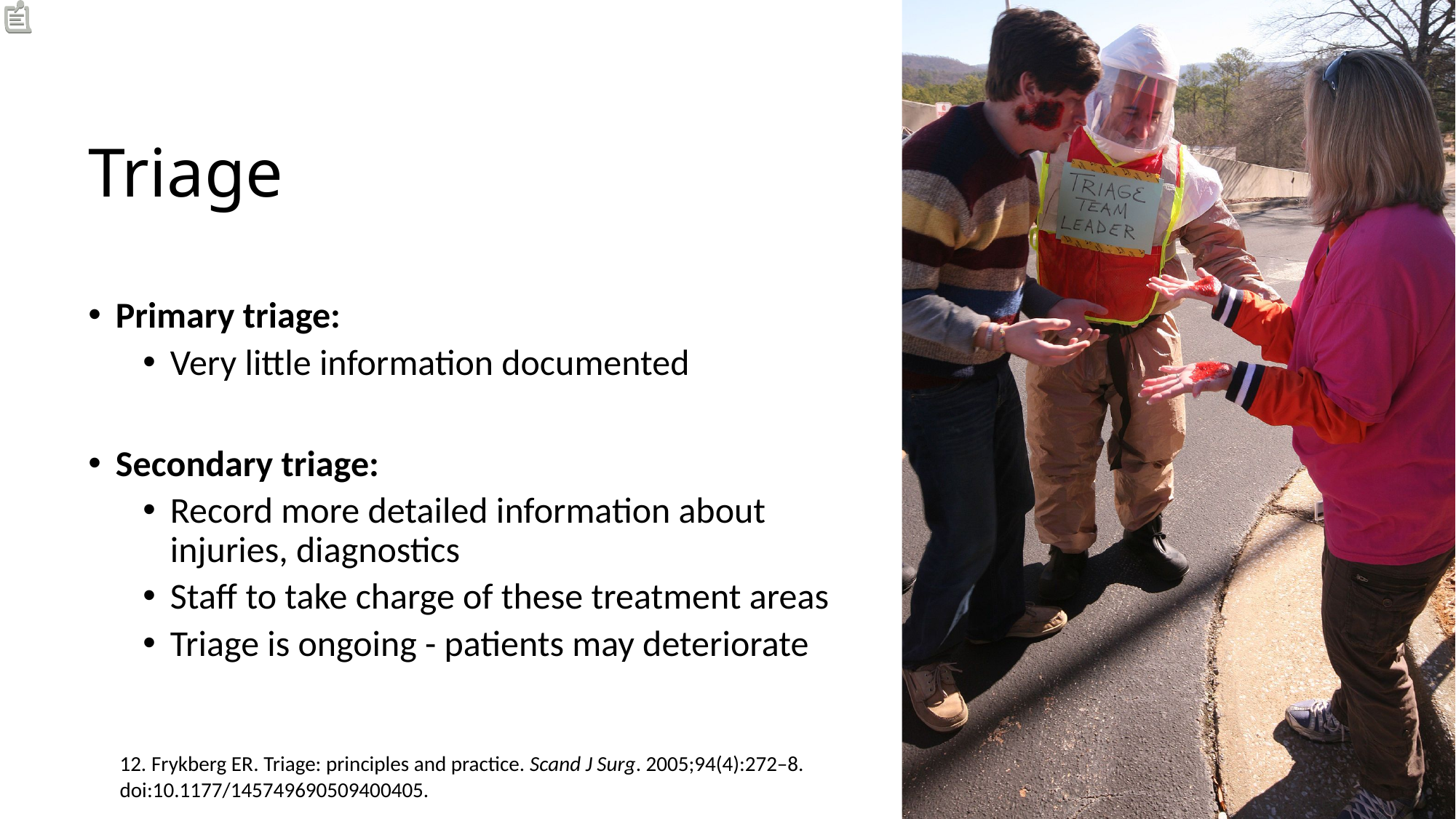

# Triage
Primary triage:
Very little information documented
Secondary triage:
Record more detailed information about injuries, diagnostics
Staff to take charge of these treatment areas
Triage is ongoing - patients may deteriorate
12. Frykberg ER. Triage: principles and practice. Scand J Surg. 2005;94(4):272–8. doi:10.1177/145749690509400405.

## Slide 5
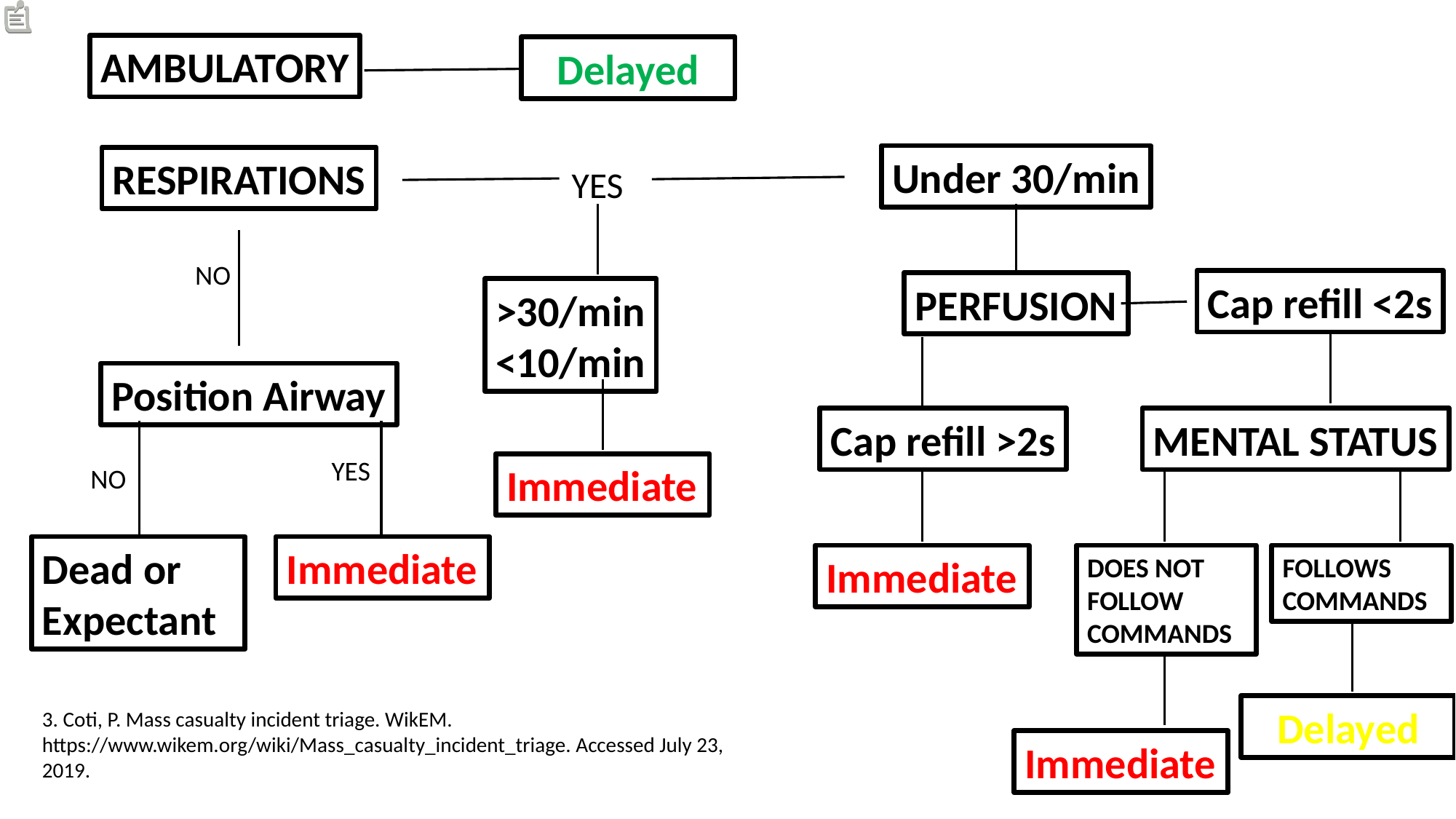

AMBULATORY
Delayed
RESPIRATIONS
NO
Position Airway
YES
NO
Immediate
Dead or Expectant
Under 30/min
Cap refill <2s
PERFUSION
Cap refill >2s
MENTAL STATUS
Immediate
DOES NOT FOLLOW
COMMANDS
FOLLOWS
COMMANDS
Delayed
Immediate
YES
>30/min
<10/min
Immediate
3. Coti, P. Mass casualty incident triage. WikEM. https://www.wikem.org/wiki/Mass_casualty_incident_triage. Accessed July 23, 2019.

## Slide 6
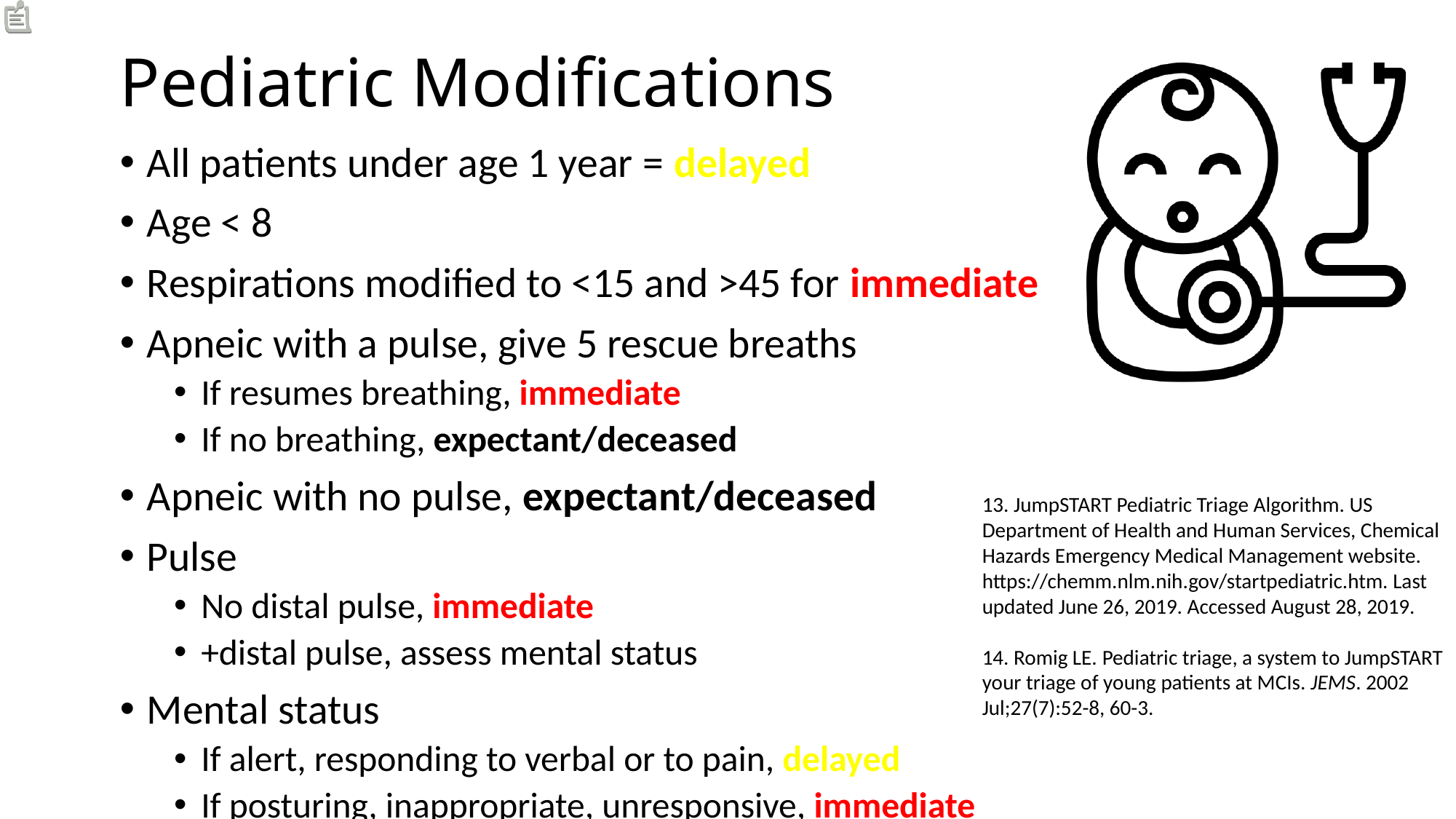

# Pediatric Modifications
All patients under age 1 year = delayed
Age < 8
Respirations modified to <15 and >45 for immediate
Apneic with a pulse, give 5 rescue breaths
If resumes breathing, immediate
If no breathing, expectant/deceased
Apneic with no pulse, expectant/deceased
Pulse
No distal pulse, immediate
+distal pulse, assess mental status
Mental status
If alert, responding to verbal or to pain, delayed
If posturing, inappropriate, unresponsive, immediate
13. JumpSTART Pediatric Triage Algorithm. US Department of Health and Human Services, Chemical Hazards Emergency Medical Management website. https://chemm.nlm.nih.gov/startpediatric.htm. Last updated June 26, 2019. Accessed August 28, 2019.
14. Romig LE. Pediatric triage, a system to JumpSTART your triage of young patients at MCIs. JEMS. 2002 Jul;27(7):52-8, 60-3.

## Slide 7
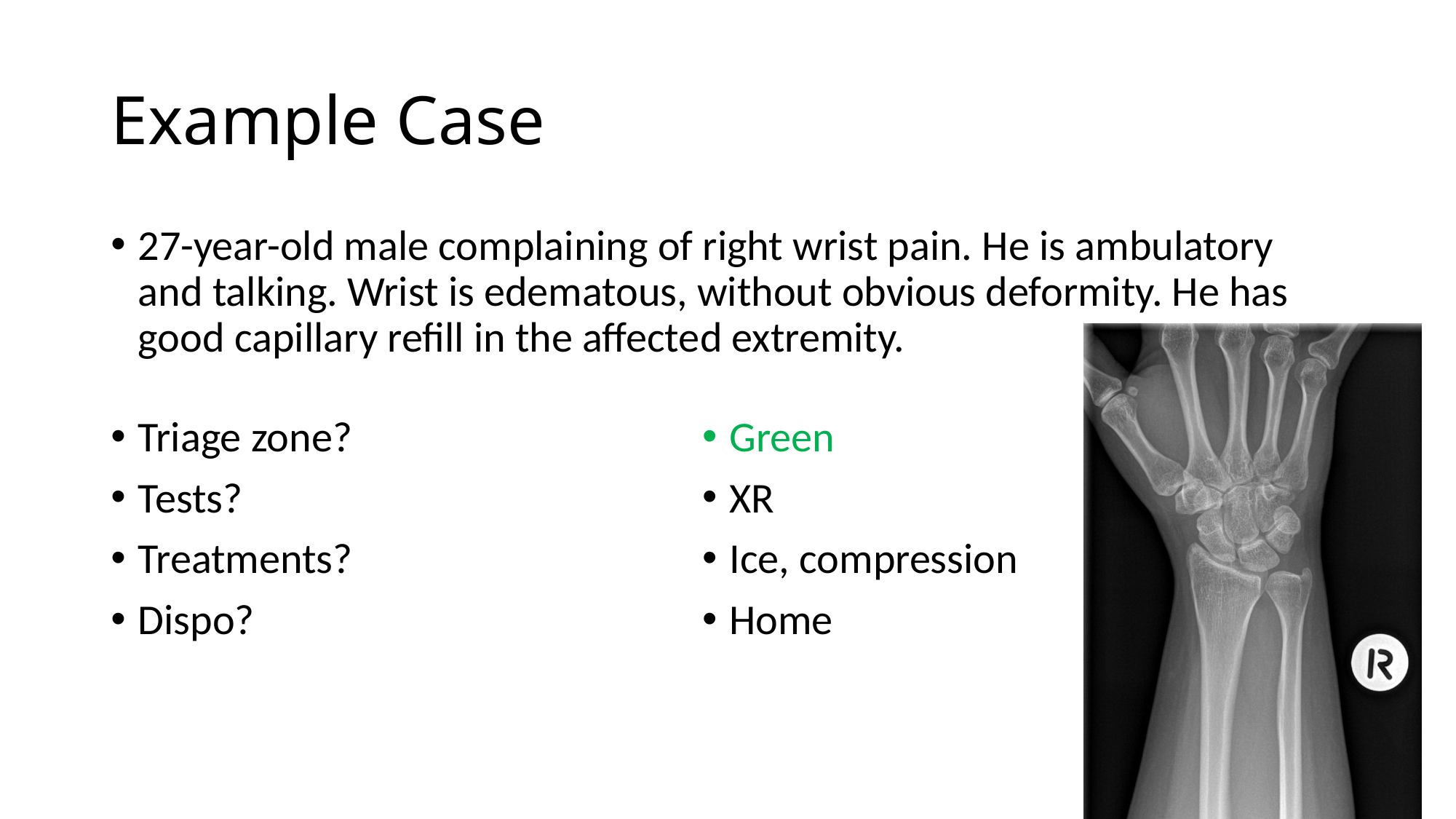

# Example Case
27-year-old male complaining of right wrist pain. He is ambulatory and talking. Wrist is edematous, without obvious deformity. He has good capillary refill in the affected extremity.
Triage zone?
Tests?
Treatments?
Dispo?
Green
XR
Ice, compression
Home

## Slide 8
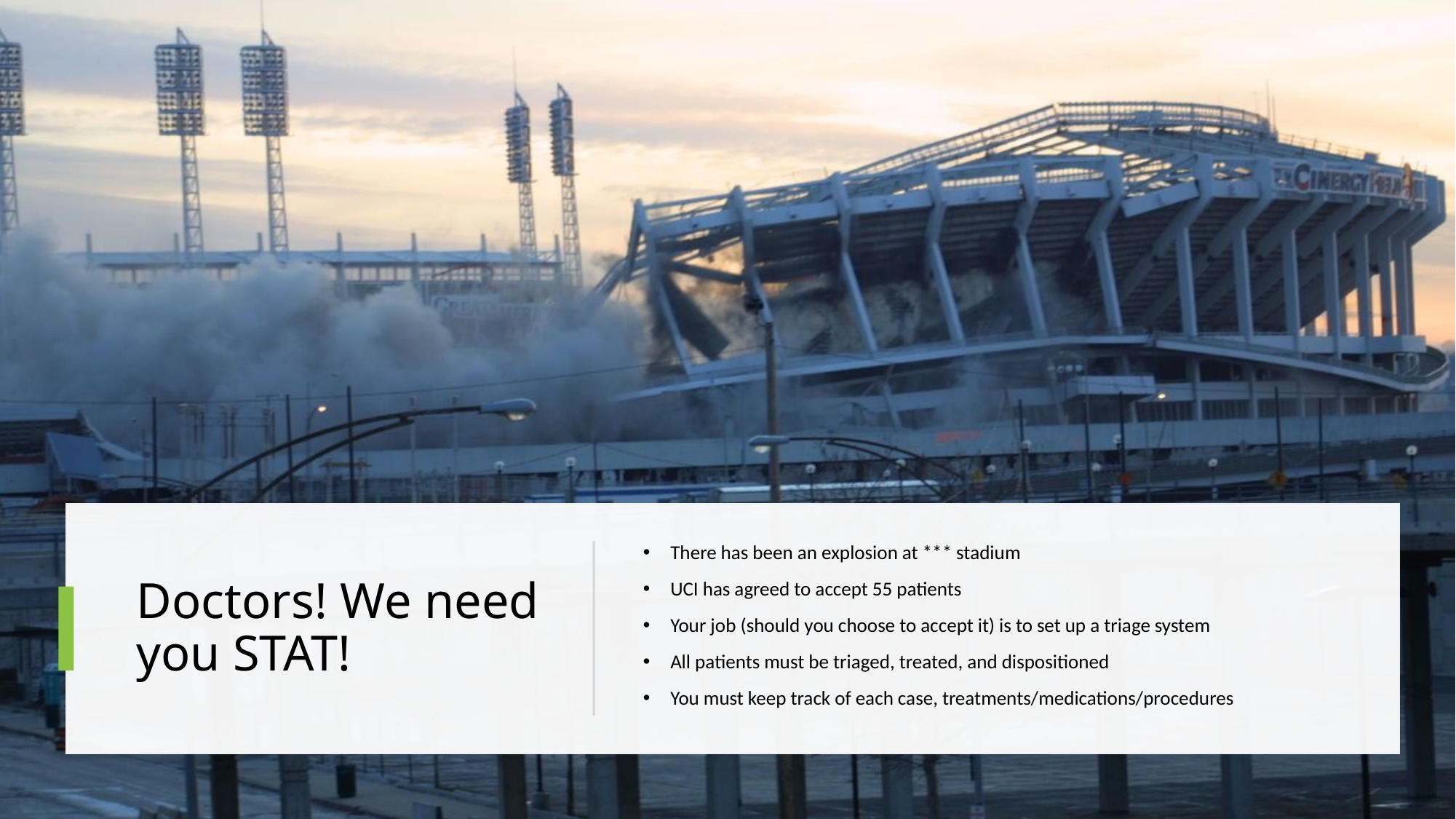

# Doctors! We need you STAT!
There has been an explosion at *** stadium
UCI has agreed to accept 55 patients
Your job (should you choose to accept it) is to set up a triage system
All patients must be triaged, treated, and dispositioned
You must keep track of each case, treatments/medications/procedures

## Slide 9
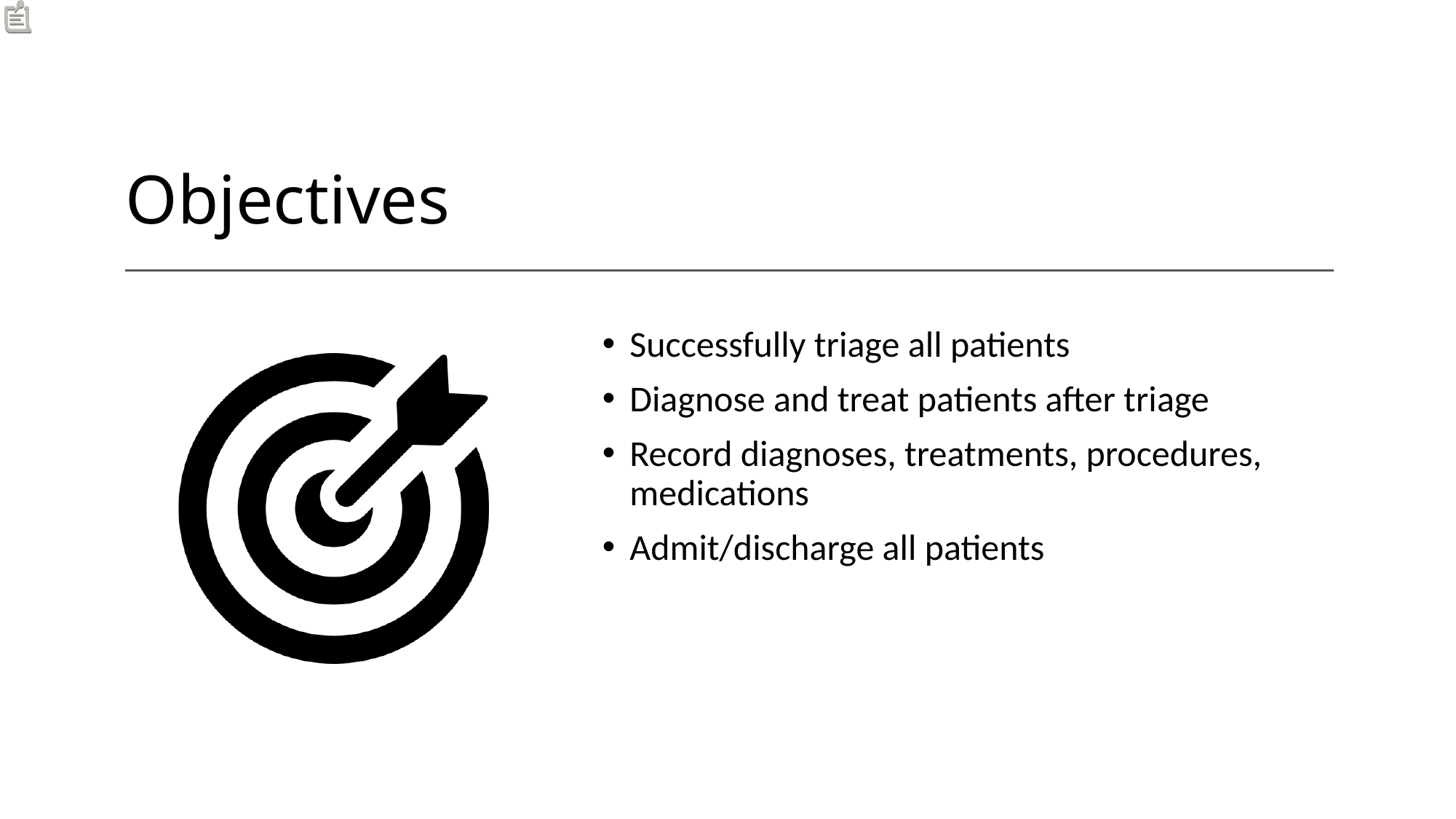

# Objectives
Successfully triage all patients
Diagnose and treat patients after triage
Record diagnoses, treatments, procedures, medications
Admit/discharge all patients

## Slide 10
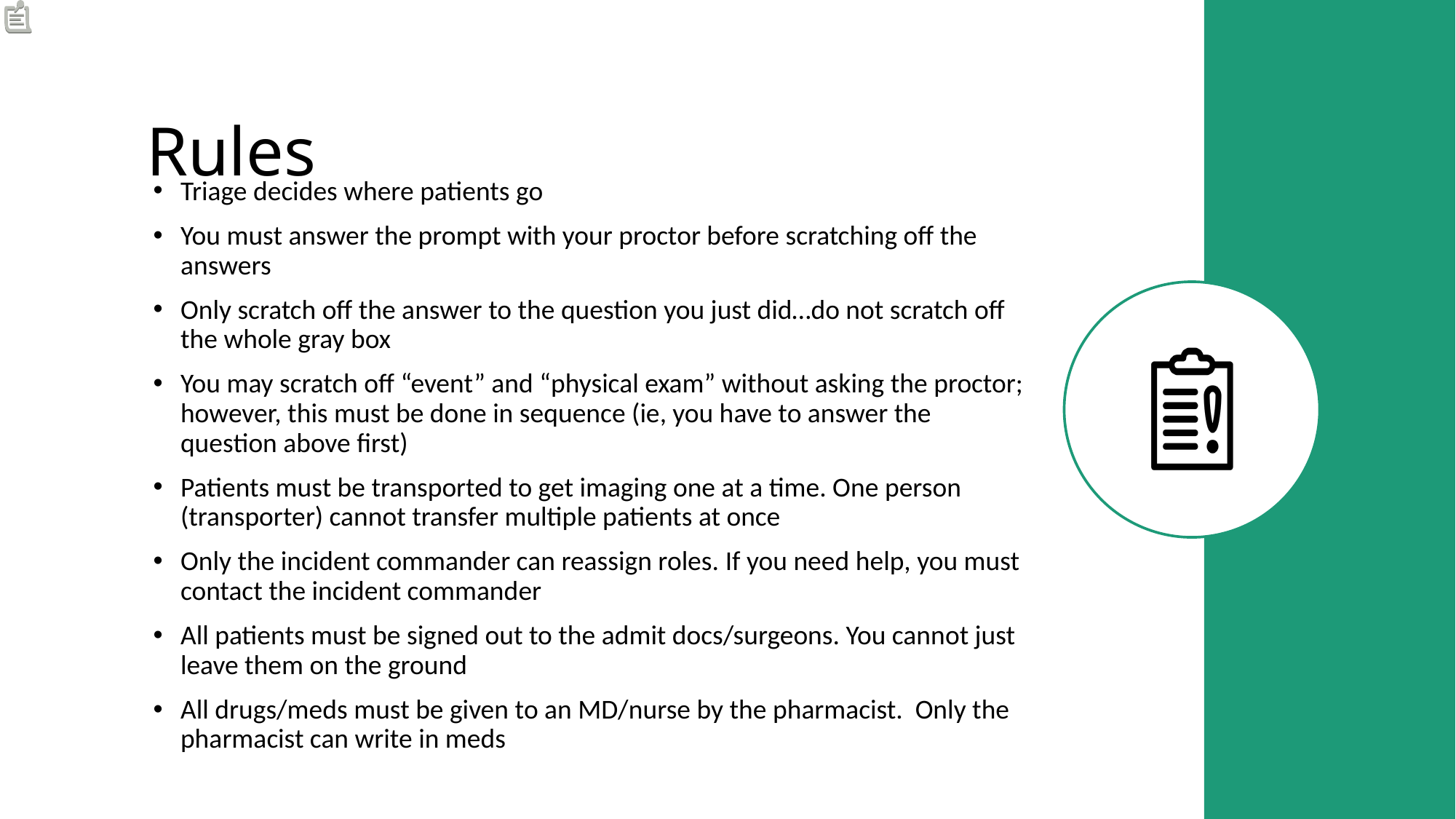

# Rules
Triage decides where patients go
You must answer the prompt with your proctor before scratching off the answers
Only scratch off the answer to the question you just did…do not scratch off the whole gray box
You may scratch off “event” and “physical exam” without asking the proctor; however, this must be done in sequence (ie, you have to answer the question above first)
Patients must be transported to get imaging one at a time. One person (transporter) cannot transfer multiple patients at once
Only the incident commander can reassign roles. If you need help, you must contact the incident commander
All patients must be signed out to the admit docs/surgeons. You cannot just leave them on the ground
All drugs/meds must be given to an MD/nurse by the pharmacist. Only the pharmacist can write in meds

## Slide 11
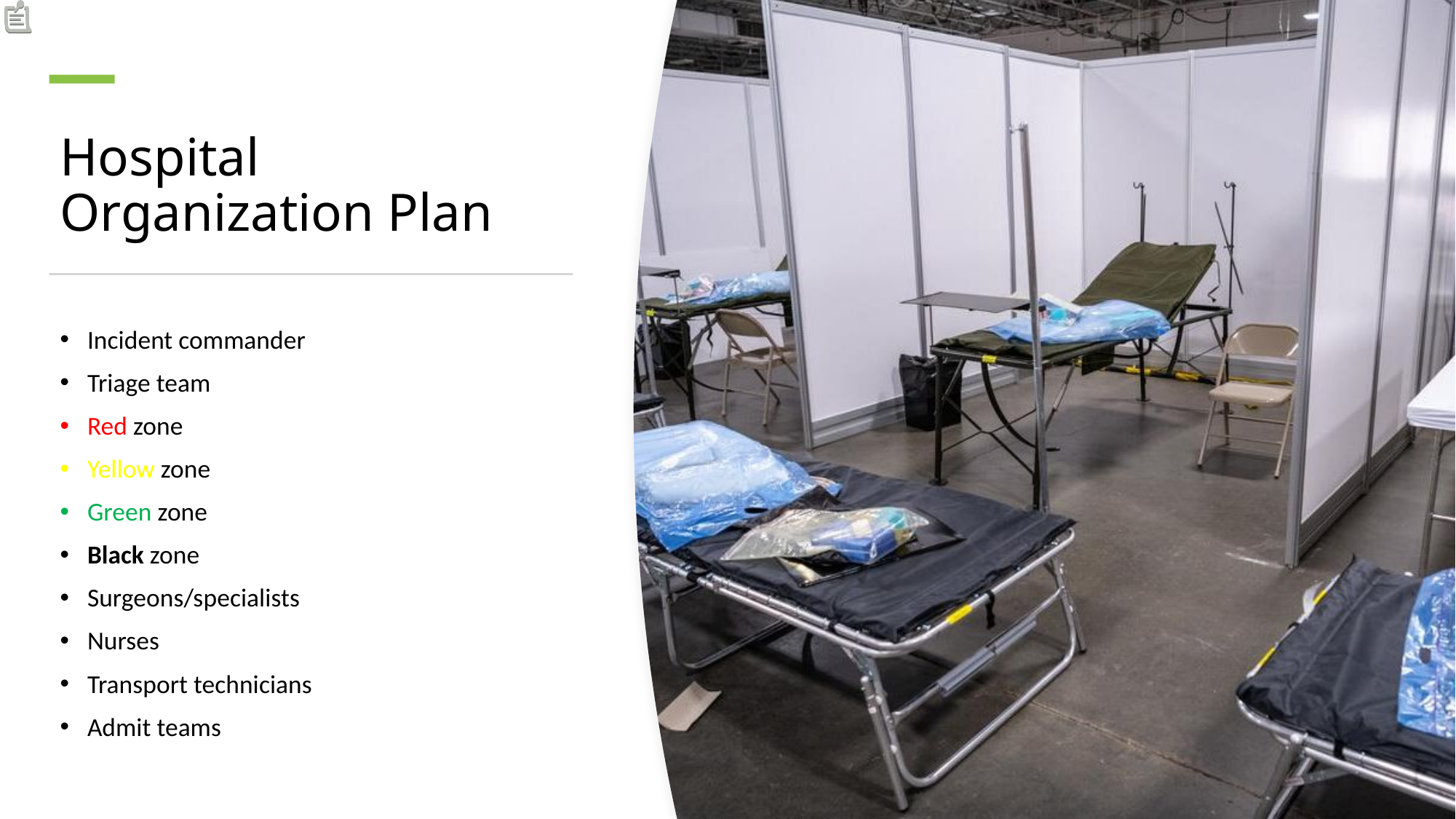

# Hospital Organization Plan
Incident commander
Triage team
Red zone
Yellow zone
Green zone
Black zone
Surgeons/specialists
Nurses
Transport technicians
Admit teams

## Slide 12
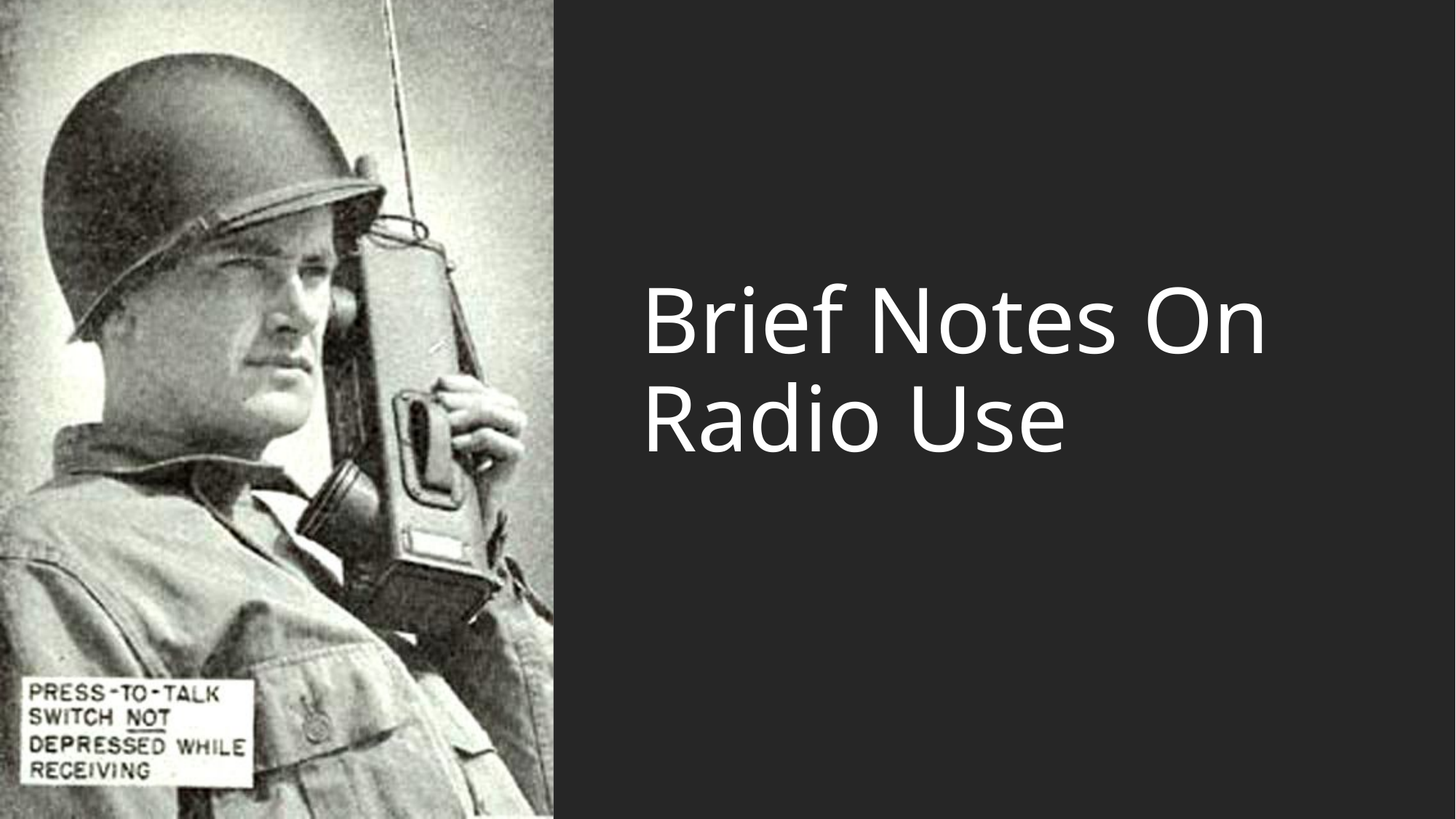

# Brief Notes On Radio Use

## Slide 13
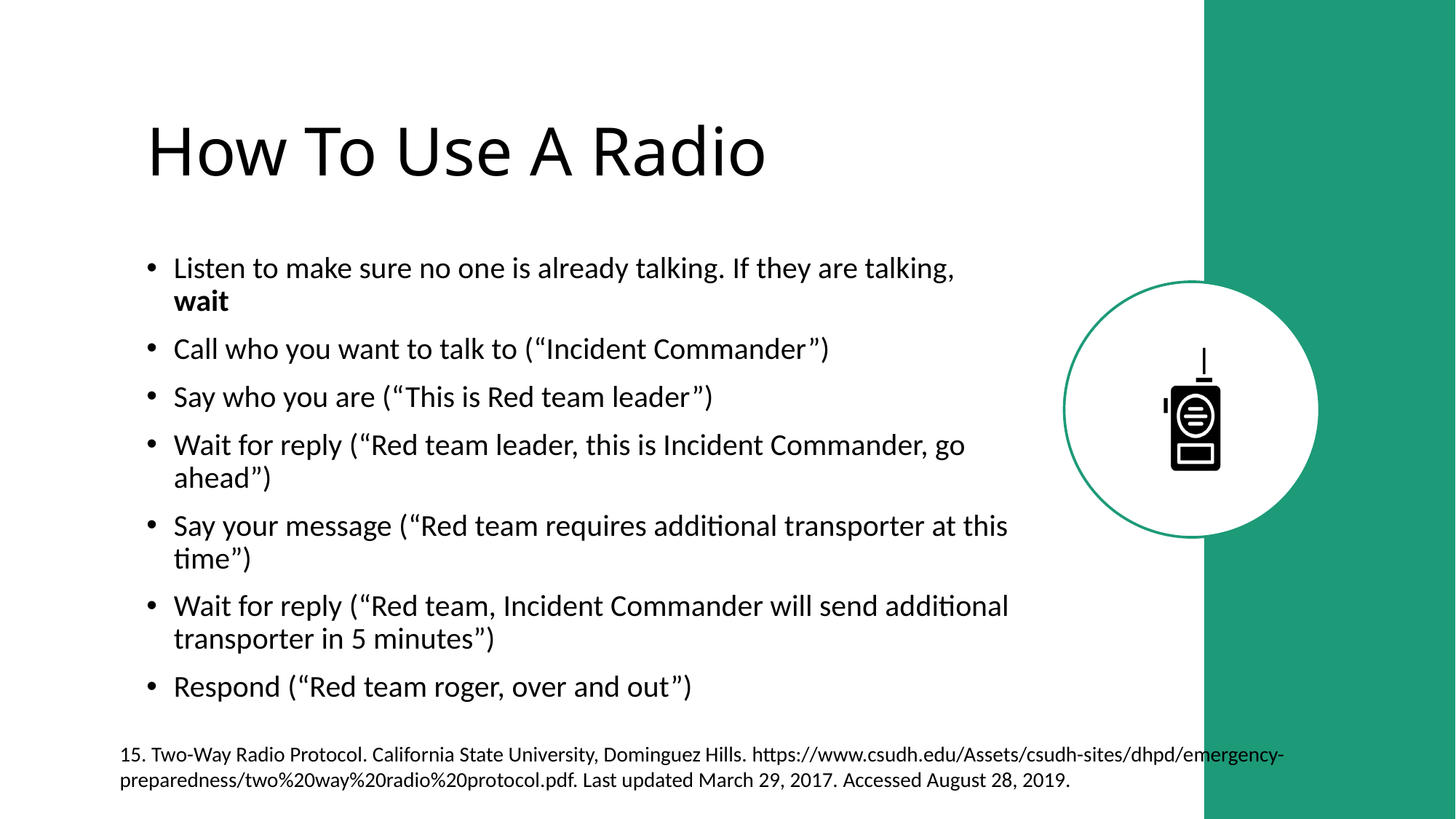

# How To Use A Radio
Listen to make sure no one is already talking. If they are talking, wait
Call who you want to talk to (“Incident Commander”)
Say who you are (“This is Red team leader”)
Wait for reply (“Red team leader, this is Incident Commander, go ahead”)
Say your message (“Red team requires additional transporter at this time”)
Wait for reply (“Red team, Incident Commander will send additional transporter in 5 minutes”)
Respond (“Red team roger, over and out”)
15. Two-Way Radio Protocol. California State University, Dominguez Hills. https://www.csudh.edu/Assets/csudh-sites/dhpd/emergency-preparedness/two%20way%20radio%20protocol.pdf. Last updated March 29, 2017. Accessed August 28, 2019.

## Slide 14
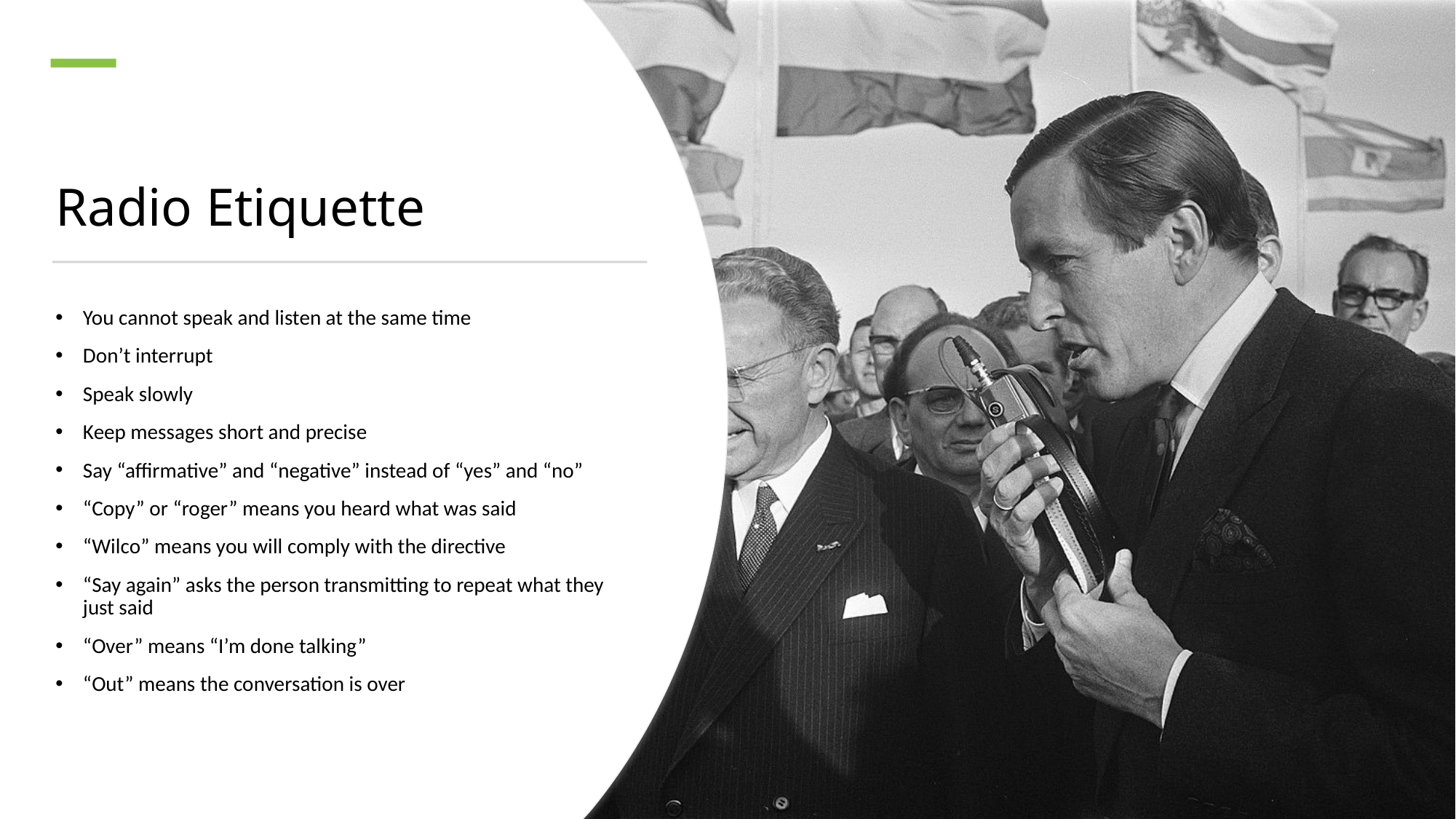

# Radio Etiquette
You cannot speak and listen at the same time
Don’t interrupt
Speak slowly
Keep messages short and precise
Say “affirmative” and “negative” instead of “yes” and “no”
“Copy” or “roger” means you heard what was said
“Wilco” means you will comply with the directive
“Say again” asks the person transmitting to repeat what they just said
“Over” means “I’m done talking”
“Out” means the conversation is over

## Slide 15
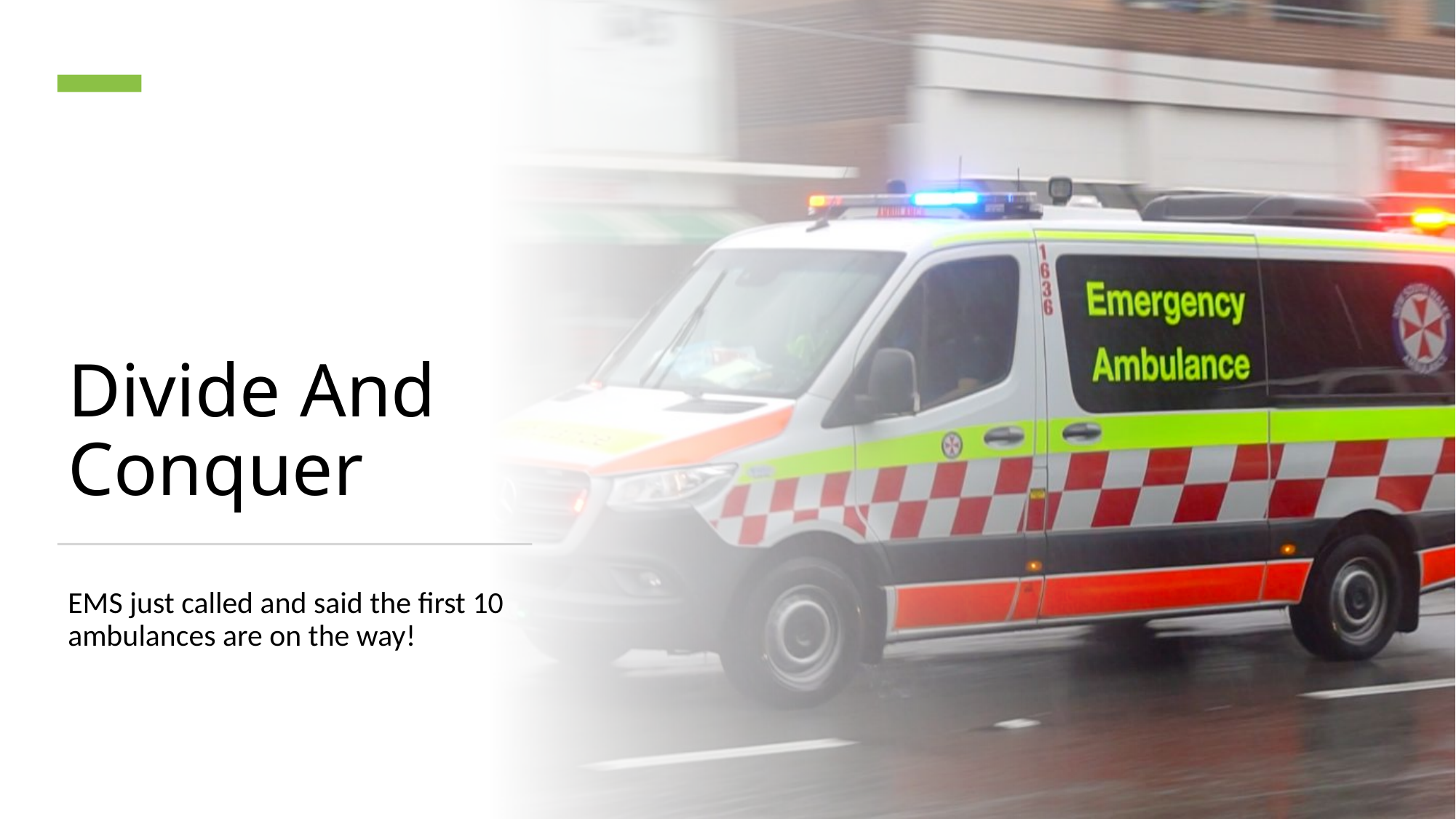

# Divide And Conquer
EMS just called and said the first 10 ambulances are on the way!

## Slide 16
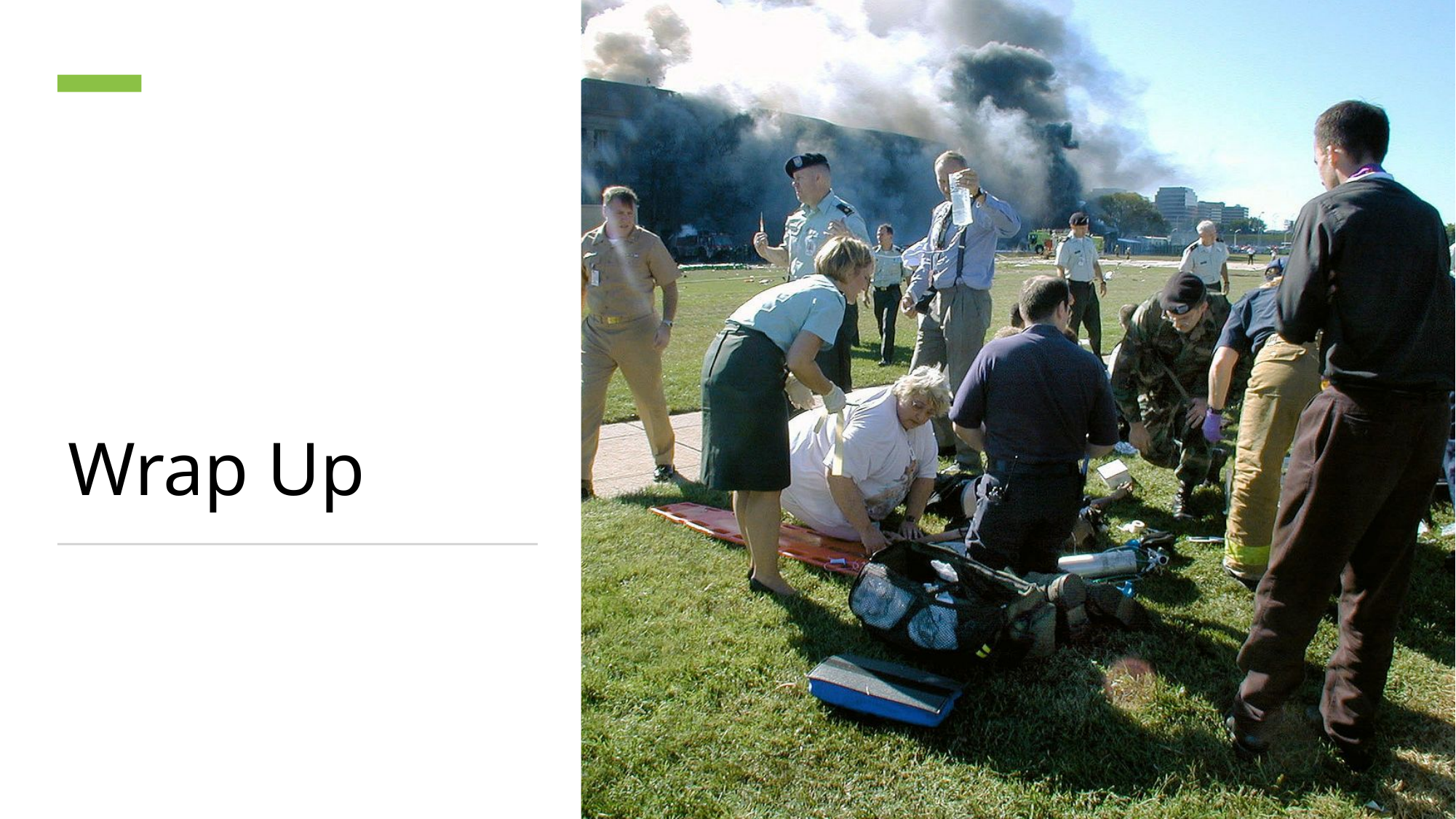

# Wrap Up

## Slide 17
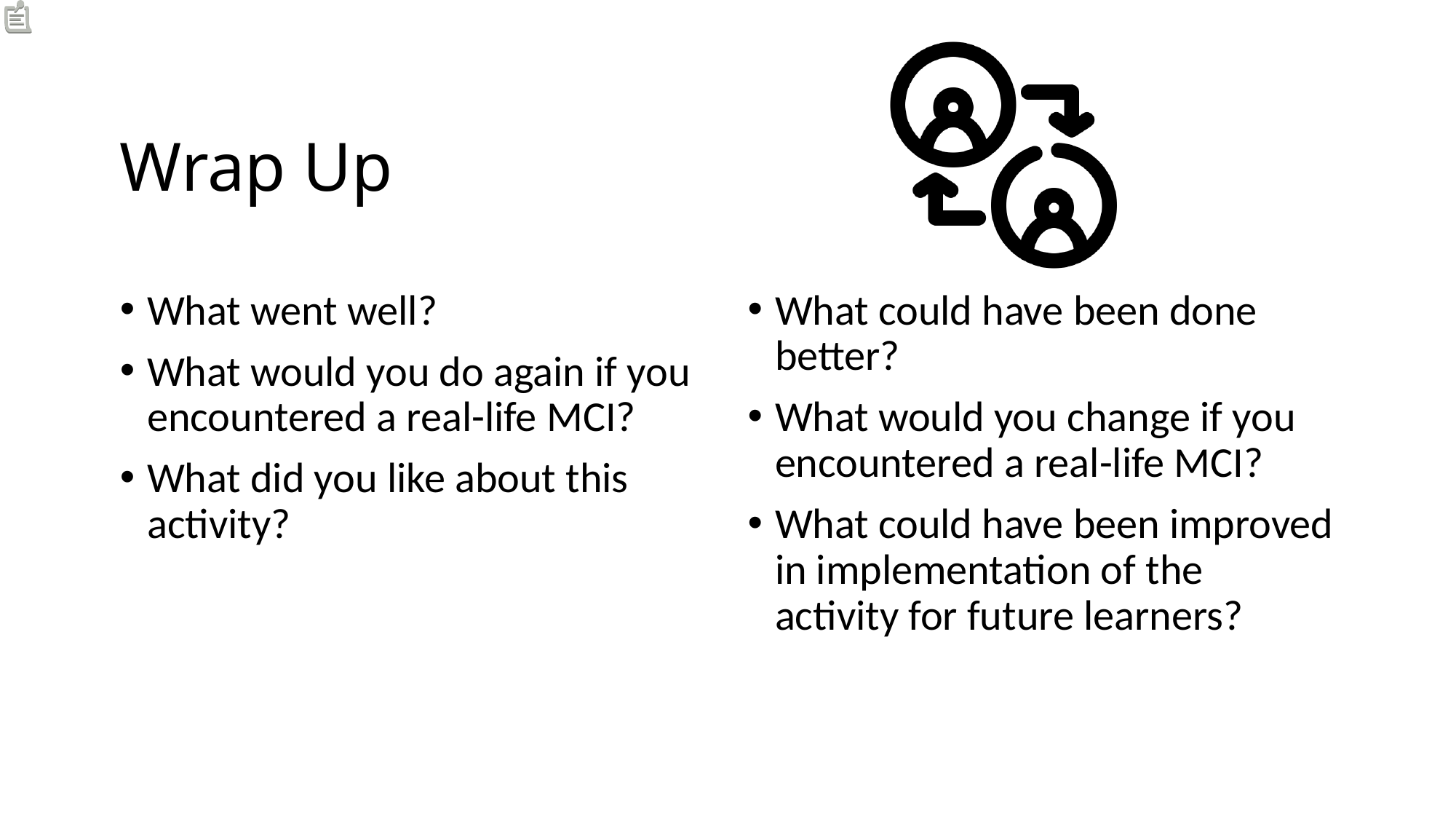

# Wrap Up
What went well?
What would you do again if you encountered a real-life MCI?
What did you like about this activity?
What could have been done better?
What would you change if you encountered a real-life MCI?
What could have been improved in implementation of the activity for future learners?
